# Supplementary material for: Evolution of the data and methods in real-world COVID-19 vaccine effectiveness studies on mortality: a scoping review protocol
Source: BMJ Open. 2024 Mar 19;14(3):e079071. doi: 10.1136/bmjopen-2023-079071 (PMC10952922; doi:10.1136/bmjopen-2023-079071)
Supplement: Supplementary data [file bmjopen-2023-079071supp001.pdf]

Results of COVID-19 Vaccine Effectiveness & Impact Studies: An Ongoing Systematic Review

Methods

Updated September 10, 2022

Prepared by:

International Vaccine Access Center,
Johns Hopkins Bloomberg School of Public Health

and

World Health Organization

and

Coalition for Epidemic Preparedness Innovations

For comments or questions, please contact: Melissa Higdon at mhigdon@jhu.edu.

Methods for Vaccine Effectiveness (VE) Literature Presented on VIEW-hub and in the Weekly Summary Tables, Visualizations, and Summaries of Policy Gaps

Literature Search

A search of the preprint, and published literature for COVID-19 Vaccine Effectiveness studies is conducted weekly. See Appendix for literature search criteria.

Inclusion Criteria for data abstraction

Title and abstract review are conducted to identify relevant studies for full-text review. During full-text review, a study must contain at least one vaccine effectiveness estimate that meets all the following criteria to be included. This is done to ensure a baseline level of quality and/or comparability of VE estimates, though this does not imply that all studies are Grade A/have minimal risk of bias nor that all excluded studies are of poor quality.

- Published or preprint studies or reports with adequate scientific details. The information cannot come just from a press release, presentations, nor media.
- VE estimates must have confidence intervals around the estimate, except in those cases where it is unable to be calculated.
- All studies must include persons with and without the clinical outcome under investigation and with and without vaccination. Thus, this excludes case only studies, such as impact studies, or those evaluating risk of progression are excluded. This criterion does not apply to transmission studies which evaluate vaccine effectiveness against secondary infection from vaccinated and unvaccinated SARS-CoV-2 cases only, or to booster dose VE studies in which the reference group is persons having completed primary series vaccination.
- The study cannot have a modeled comparison group nor compare to a historical cohort.
- Due to the effect of confounders, the study design should account for confounding and/or the VE estimate should be adjusted or state adjustment made no difference.
- All outcomes must be lab confirmed. As COVID-19 does not have a specific syndrome, studies with syndromic outcomes are excluded.
- At least 90% of participants must have a confirmed vaccination status, rather than relying on recall.
- The study must provide a VE estimate for one vaccine, not for multiple vaccines combined. The exceptions are for 1) studies assessing the combined VE of BNT162b2 (Pfizer) and mRNA-1273 (Moderna) vaccines, 2) studies of heterologous schedules but all participants included in a VE estimate should receive the same brands of vaccines in the same order, and 3) studies of vaccine effectiveness against transmission (due to the scarcity of transmission studies).
- No significant bias that likely affects results
- Cannot include day 0-12 in unvaccinated definition
- Cannot compare to early post vaccination to calculate VE (e.g. day 0-12 vs day 12-21)

A summary table of the main results of studies meeting inclusion criteria can be found on the VIEW-hub Resources page (https://view-hub.org/resources).

Inclusion Criteria for Forest Plots Posed on VIEW-Hub

The VE estimates from eligible studies are plotted in figures. The estimates plotted are a subset of the estimates abstracted from the systematic literature review of those studies meeting additional eligibility criteria. Because a single study can include many VE estimates where the same data appear in more than one VE estimate (e.g., all ages and also separately by age group), criteria are applied to prioritize which to plot in an effort to not overrepresent the amount of evidence that exists for each vaccine. The following criteria are used to determine which VE estimates are displayed in the summary forest plots located on the VIEW-hub resources page (https://view-hub.org/resources):

- Complete vaccination is defined as ≥7 days post final dose; partial vaccination is defined as ≥14 days post first dose of a 2-dose vaccine (current forest plots display VE estimates for complete primary series, first booster dose, and second booster dose; partial vaccination is no longer shown).
- If a study reports results for the same outcome for both combined and individual vaccines, only individual vaccine VE estimates are displayed. This criterion only apples to studies evaluating VE of BNT162b2 (Pfizer) mRNA-1273 (Moderna) vaccines.
- If a study reports results from 2 different evaluation designs (e.g. test-negative design and cohort design) on the same population, VE estimates from the primary analysis only are displayed.
- If a study reports VE estimates for the same disease outcome for different populations, the general population VE estimate is displayed when available. If a general population estimate is not available, the VE from each population is displayed (exception is if there are estimates for similar age groups in which case the more stable VE estimate will be displayed).
- If a study reports VE estimates on more than one 'severe' disease outcome (e.g. 'severe disease', 'hospitalization', and 'ICU admission'), the more inclusive disease outcome including a larger population is displayed. These different types of severe outcomes are labeled as 'severe disease' in the plots, however it is important to keep in mind that the definition of severe disease varies and may explain some differences in VE estimates for severe disease outcomes.
- If a study reports VE estimates for a specific regimen and population at different time intervals since vaccination, the earliest interval of peak VE is selected for the vaccine-specific forest plots (with an exception for the plots on duration of vaccine effectiveness in which multiple time points are plotted). Studies that report only one VE estimate for a specific regimen and population (i.e. at ≥ 14 days post final dose) are included in the vaccine-specific plots and denoted with a '+' after the reference id if the time interval post-vaccination over which VE is measured extends beyond 4 months.

Additional notes

Estimates from mutually exclusive populations in a study can be displayed in the same plot resulting in instances when more than one estimate from a study is plotted (e.g., a study includes VE estimates from two distinct age groups or estimates for different variants).

For studies that report adjusted odds ratios, risk ratios, or rate ratios instead of vaccine effectiveness estimates, VE is calculated as 1 minus the reported effect estimate and multiplying by 100.

INTERNATIONAL VACCINE ACCESS CENTER World Health Organization CEPI

Reference numbers are included for each VE estimate displayed so users can identify when a study is represented more than once within a plot. More information on each reference can be found in the weekly literature review summary table located on VIEW-HUB (https://view-hub.org/resources).

Vaccine Effectiveness Studies Database

See accompanying PDF ('CEPI_COVID19VaccineEffectiveness.pdf') of detailed data collection forms for COVID-19 vaccine effectiveness studies. The complete vaccine effectiveness studies database will be made available to CEPI at anytime upon request. In addition, a summary PDF file of all abstracted VE estimates is available on the VIEW-hub Resources page. The same information is also available in a downloadable filterable Excel file ('COVID-19 Vaccine Effectiveness Results Dataset'). These materials are available to the public and updated weekly.

Planned and Ongoing Studies presented on VIEW-hub and summaries of policy gaps

In order to gather information on planned and ongoing studies, a survey was shared with persons conducting and/or funding studies. Data was requested specifically on studies that have completed protocol development to help obtain higher quality data as studies that are still in protocol development are subject to more changes. This data has been compiled by WHO and some key information and summaries of what is planned/ongoing are provided on View Hub and WHO's website.

Appendix: Literature Search Terms

PubMed:

("COVID-19"[tw] OR "COVID 19"[tw] OR "COVID19"[tw] OR "COVID2019"[tw] OR "COVID 2019"[tw] OR "COVID-2019"[tw] OR "novel coronavirus"[tw] OR "new coronavirus"[tw] OR "novel corona virus"[tw] OR "new corona virus"[tw] OR "SARS-CoV-2"[tw] OR "SARSCoV2"[tw] OR "SARS-CoV2"[tw] OR "2019nCoV"[tw] OR "2019-nCoV"[tw] OR "2019 coronavirus"[tw] OR "2019 corona virus"[tw] OR "coronavirus disease 2019"[tw] OR "severe acute respiratory syndrome coronavirus 2"[nm] OR "severe acute respiratory syndrome coronavirus 2"[tw] OR "sars-coronavirus-2"[tw] OR "coronavirus disease 2019"[tw] OR "corona virus disease 2019"[tw])

AND

("COVID-19 Vaccines"[Mesh] OR “COVID-19 vaccine”[tiab] OR "mRNA-1273 vaccine" [Supplementary Concept] OR “mRNA-1273 vaccine”[tiab] OR “mRNA vaccine”[tiab] OR “mRNA COVID-19 vaccines”[tiab] OR "ChAdOx1 COVID-19 vaccine" [Supplementary Concept] OR "Ad5-nCoV vaccine" [Supplementary Concept] OR “Ad5-nCoV”[tiab] OR "Covid-19 aAPC vaccine" [Supplementary Concept] OR "Ad26.COV2.S vaccine" [Supplementary Concept] OR "Ad26.COV2.S vaccine"[tiab] OR “adenoviral vector vaccine”[tiab] OR "BNT162 vaccine" [Supplementary Concept] OR “BNT162b2”[tiab] OR “BNT162”[tiab] OR “CoronaVac” [tiab] OR “vaccin*”[tiab])

AND

("Clinical Trial, Phase IV" [Publication Type] OR "Controlled Clinical Trial" [Publication Type] OR "Randomized Controlled Trial" [Publication Type] OR "Case-Control Studies"[Mesh] OR "Retrospective Studies"[Mesh] OR “Retrospective”[tiab] OR "Cohort Studies"[Mesh] OR "Prospective Studies"[Mesh] OR “Prospective”[tiab] OR "Longitudinal Studies"[Mesh] OR "Follow-Up Studies"[Mesh] OR “Follow-up studies”[tiab] OR “cohort”[tiab] OR "test negative"[tiab] OR “Observational cohort”[tiab] OR “Test-negative design”[tiab] OR"RCT"[tiab] OR "randomized"[tiab] OR "randomised"[tiab] OR "randomly allocated"[tiab] OR “case-control”[tiab] OR “real-world effectiveness”[tiab] OR “effectiveness”[tiab] OR “association”[tiab] OR “impact”[tiab] OR “vaccine impact”[tiab]) NOT ("Clinical Trial, Phase I" [Publication Type] OR "Clinical Trial, Phase II" [Publication Type])
NOT (“animals”[mesh] NOT (“animals”[mesh] AND “humans”[mesh]))

Embase:

('COVID-19' OR 'COVID 19' OR 'COVID19' OR 'COVID2019' OR 'COVID 2019' OR 'COVID-2019' OR 'novel coronavirus' OR 'new coronavirus' OR 'novel corona virus' OR 'new corona virus' OR 'SARS-CoV-2' OR 'SARSCoV2' OR 'SARS-CoV2' OR '2019nCoV' OR '2019-nCoV' OR '2019 coronavirus' OR '2019 corona virus' OR 'coronavirus disease 2019' OR 'severe acute respiratory

syndrome coronavirus 2'/exp OR 'severe acute respiratory syndrome coronavirus 2' OR 'sars-coronavirus-2' OR 'coronavirus disease 2019'/exp OR 'coronavirus disease 2019' OR 'corona virus disease 2019')

AND

('SARS-CoV-2 vaccine'/exp OR 'COVID-19 vaccine':ti,ab OR 'mRNA-1273 vaccine'/exp OR 'mRNA-1273 vaccine':ti,ab OR 'mRNA vaccine':ti,ab OR 'mRNA COVID-19 vaccines':ti,ab OR 'ChAdOx1 ncov 19'/exp OR 'Ad5 nCoV vaccine'/exp OR 'Ad5-nCoV':ti,ab OR 'Covid-19 aAPC vaccine':ti,ab OR 'Ad26.COV2.S vaccine'/exp OR 'Ad26.COV2.S vaccine':ti,ab OR 'adenoviral vector vaccine':ti,ab OR 'BNT 162 vaccine'/exp OR 'BNT162b2':ti,ab OR 'BNT162':ti,ab OR 'CoronaVac'/exp OR 'coronavac':ti,ab OR 'vaccin*':ti,ab)

AND

('phase 4 clinical trial'/exp OR 'Controlled Clinical Trial'/exp OR 'Randomized Controlled Trial'/exp OR 'Case Control Study'/exp OR 'Retrospective Study'/exp OR 'Retrospective':ti,ab OR 'Cohort analysis'/exp OR 'Prospective Study'/exp OR 'Prospective':ti,ab OR 'Longitudinal Study'/exp OR 'Follow Up'/exp OR 'Follow-up study':ti,ab OR 'cohort':ti,ab OR 'test negative':ti,ab OR 'Observational cohort':ti,ab OR 'postmarketing surveillance'/exp OR 'postmarketing surveillance':ti,ab OR 'Test-negative design':ti,ab OR 'RCT':ti,ab OR 'randomized':ti,ab OR 'randomised':ti,ab OR 'randomly allocated':ti,ab OR 'case-control':ti,ab OR 'real-world effectiveness':ti,ab OR 'effectiveness':ti,ab OR 'association':ti,ab) NOT ('phase 1 clinical trial'/exp OR 'phase 2 clinical trial'/exp)
NOT ('animal'/exp NOT ('animal'/exp AND 'human'/exp))
NOT 'conference abstract'/it

<u>WHO COVID database:</u>

("COVID-19 Vaccines" OR "COVID-19 vaccine" OR "mRNA-1273 vaccine" OR "mRNA vaccine" OR "mRNA COVID-19 vaccines" OR "ChAdOx1 COVID-19 vaccine" OR "Ad5-nCoV" OR "Covid-19 aAPC vaccine" OR "Ad26.COV2.S vaccine" OR "adenoviral vector vaccine" OR "BNT162b2" OR "BNT162" OR "CoronaVac" OR vaccin*)

AND

("Phase IV" OR "Controlled Clinical Trial" OR "Randomized Controlled Trial" OR "Case-Control Studies" OR "Retrospective" OR "Cohort Studies" OR "Prospective" OR "Longitudinal Studies" OR "Follow-Up Studies" OR "Follow-up study" OR "cohort" OR "test negative" OR "Observational cohort" OR "Test-negative design" OR "RCT" OR "randomized" OR "randomised" OR "randomly allocated" OR "case-control" OR "real-world effectiveness" OR "effectiveness" OR "association") AND NOT ("Phase I" OR "Phase II")

<u>SCOPUS:</u>

TITLE-ABS-KEY(“novel coronavir*” OR “novel corona virus*” OR “2019 coronavirus” OR betacoronavir* OR covid19 OR "covid 19" OR ncov OR "CoV 2" OR cov2 OR sarscov2 OR sars-cov OR sarscov OR 2019ncov OR 2019-nCoV OR "novel CoV" OR “coronavirus infections”) AND TITLE-ABS-KEY(Vaccin* AND (effectiveness OR efficacy OR protection*) AND (postmarketing OR approved OR (post* W/5 approval) OR "real world" OR "phase IV" OR "phase 4" OR observational OR longitudinal OR spread OR transmission OR (rate* W/5 infection*) OR (reduc* W/5 infection*) OR "general population"))

Web of Science:

(TI=(covid-19 vaccine effectiveness)) OR AB=(covid-19 vaccine effectiveness)

medRxiv, bioRxiv, SSRN, Europe PMC, Research Square, Knowledge Hub:

“COVID-19 vaccine effectiveness” OR “COVID-19 vaccine efficacy”

In addition to the above databases, MMWR, and Eurosurveillance are hand-searched weekly for new studies meeting eligibility criteria.
