# Supplementary material for: Evolution of the data and methods in real-world COVID-19 vaccine effectiveness studies on mortality: a scoping review protocol
Source: BMJ Open. 2024 Mar 19;14(3):e079071. doi: 10.1136/bmjopen-2023-079071 (PMC10952922; doi:10.1136/bmjopen-2023-079071)
Supplement: Author's manuscript [file bmjopen-2023-079071.draft_revisions.pdf]

BMJ Open

BMJ Open is committed to open peer review. As part of this commitment we make the peer review history of every article we publish publicly available.

When an article is published we post the peer reviewers' comments and the authors' responses online. We also post the versions of the paper that were used during peer review. These are the versions that the peer review comments apply to.

The versions of the paper that follow are the versions that were submitted during the peer review process. They are not the versions of record or the final published versions. They should not be cited or distributed as the published version of this manuscript.

BMJ Open is an open access journal and the full, final, typeset and author-corrected version of record of the manuscript is available on our site with no access controls, subscription charges or pay-per-view fees (http://bmjopen.bmj.com).

If you have any questions on BMJ Open's open peer review process please email info.bmjopen@bmj.com

BMJ Open

EVOLution of the data and methods in real world COVID-19 Vaccine Effectiveness studies on mortality: A Scoping Review Protocol

Journal:	*BMJ Open*
Manuscript ID	bmjopen-2023-079071
Article Type:	Protocol
Date Submitted by the Author:	21-Aug-2023

Complete List of Authors:	Stehlik, Paulina; Griffith University Faculty of Health, School of Pharmacy and Medical Sciences; Bond University Faculty of Health Sciences and Medicine, Institute for Evidence-Based Healthcare<br>Dowsett, Caroline; Griffith University Faculty of Health, School of Pharmacy and Medical Sciences<br>Camacho, Ximena; University of New South Wales Medicine & Health, School of Population Health<br>Falster, Michael; University of New South Wales Medicine & Health, School of Population Health<br>Lim, Renly; University of South Australia Division of Health Sciences, Quality Use of Medicines and Pharmacy Research Centre<br>Nasreen, Sharifa; University of Toronto, Dalla Lana School of Public Health<br>Pratt, Nicole; University of South Australia Clinical & Health Sciences Academic Unit, Quality Use of Medicines and Pharmacy Research Centre<br>Pearson, Sallie-Anne; University of New South Wales, School of Population Health<br>Henry, David; Bond University Faculty of Health Sciences and Medicine, Institute for Evidence-Based Healthcare; University of New South Wales, School of Population Health

Keywords:	COVID-19, STATISTICS & RESEARCH METHODS, Protocols & guidelines < HEALTH SERVICES ADMINISTRATION & MANAGEMENT, INFECTIOUS DISEASES, Mortality, PUBLIC HEALTH

SCHOLARONE™ Manuscripts

EVOLution of the data and methods in real world COVID-19 Vaccine Effectiveness studies on mortality: A Scoping Review Protocol

Short title: EVOLVE ScR

FORWARD

AUTHORS

Paulina Stehlik, School of Pharmacy and Medical Sciences, Griffith University; Adjunct Faculty of Health Sciences and Medicine, Bond University

Caroline Dowsett, School of Pharmacy and Medical Sciences, Griffith University.

Ximena Camacho, School of Population Health, Faculty of Medicine and Health, University of New South Wales

Michael Falster, School of Population Health, Faculty of Medicine and Health, University of New South Wales

Renly Lim, Quality Use of Medicines and Pharmacy Research Centre, UniSA Clinical and Health Sciences, University of South Australia

Sharifa Nasreen, Dalla Lana School of Public Health, University of Toronto; ICES, Toronto

Nicole Pratt, Quality Use of Medicines and Pharmacy Research Centre, UniSA Clinical and Health Sciences, University of South Australia

Sallie-Anne Pearson, School of Population Health, Faculty of Medicine and Health, University of New South Wales

David Henry, Institute for Evidence-Based Healthcare, Bond University and School of Population Health, Faculty of Medicine and Health, University of New South Wales

ADVISORY GROUP

Bette Liu, UNSW National Centre for Immunisation Research and Surveillance

Andrew Wilson, PBAC advisory committee chair; Director of the Menzies Centre for Health Policy, Menzies Centre for Health Policy, School of Public Health

For peer review only

Kylie Alcorn, A/Director Infectious Diseases, Infectious Disease Department, Gold Coast Health

Alexandra Bannach-Brown, Charité - Universitätsmedizin Berlin

CONTRIBUTION (CREDIT AUTHOR STATEMENT)

- Conceptualization: DH, PS
- Methodology: All authors
- Validation: PS
- Data synthesis: PS, XC, MF
- Investigation: All authors
- Resources: PS
- Data Curation: PS – DB design and management; PS, DH, XC – Piloting; CD – data extraction
- Writing - Original Draft: PS, DH
- Writing - Review & Editing: All authors (acknowledgement to advisory group and Oyungerel Byambasuren)
- Visualization: PS
- Supervision: DH, PS
- Project administration: PS
- Funding acquisition: PS, DH

FUNDING

Medicines Intelligence Centre for Research Excellence (MI-CRE) 2022 Project Incubator Grant; The MI-CRE is supported by the National Health and Medical Research Council's Centres of Research Excellence (CRE) scheme (ID 1196900)

RL is supported by a National Health and Medical Research Council (NHMRC) Early Career Fellowship (APP1156368).

MF is supported by a Future Leader Fellowship from the National Heart Foundation of Australia (ID: 105609).

XC is supported by a NHMRC Postgraduate Scholarship (ID: 2005259).

CONFLICTS OF INTEREST

No additional conflicts of interest to declare.

For peer review only

ABSTRACT

Background: Early evidence on COVID-19 vaccine efficacy came from randomised trials. However, many important questions about vaccine effectiveness (VE) in vulnerable groups and evolving viral variants have been addressed using real-world data. The results of these studies have informed most vaccination policies globally. As the questions about VE have evolved during the pandemic so have data, study design and analytical choices. This scoping review aims to characterise this evolution and provide insights for future pandemic planning – specifically, what kinds of questions are asked at which stages of a pandemic, and what data infrastructure and methods are used to answer these questions?

Methods and analysis: We will identify relevant studies in the Johns Hopkins Bloomberg School of Public Health VIEW-hub database. We will include real-world studies of COVID-19 VE that reported COVID-19-specific and all-cause mortality. We will extract information on study characteristics; study context; data sources; design and analytic methods that deal with confounding.

A single reviewer will extract data after achieving 80% agreement on a validation set. Variables such as data sources will be categorised using an inductive approach and each study will be discussed in a small group setting. A timeline mapping approach will be used to capture the evolution of this body of literature. Within-country activities will be documented to discern the development of data design and analytic strategies.

Dissemination: This review will provide important information on how study questions, data availability and resulting design choices of VE studies evolved through the COVID-19 pandemic. This review will help identify options for planning and VE studies and inform policy makers on the minimal data and analytic infrastructure needed to support rapid real-world evaluation of VE in future pandemics. The findings will also be relevant to initiatives to rapidly evaluate effectiveness of health care strategies more broadly.

Registration: https://doi.org/10.17605/OSF.IO/ZHDKR

STRENGTHS AND LIMITATIONS

- This will be the first comprehensive mapping of the evolution of the literature on real world effectiveness studies of the impacts of COVID-19 vaccines on mortality.
- Findings will provide valuable information on how questions, data, design and analytic choices change during a pandemic.

For peer review only

- We will use a curated database (Johns Hopkins Bloomberg School of Public Health VIEW-hub) to identify relevant studies of the impact of Covid-19 vaccines on mortality. While this may mean we miss relevant studies, studies in this database are required to meet a minimal set of quality criteria meaning that findings are more likely to be aligned with best research practices.
- We will categorise our data in meaningful ways to facilitate data synthesis and mapping. However, to allow for detailed exploration we will produce interactive visuals so that researchers and policy makers are able to explore the data independently and make our data open access at the conclusion of our study.
- Data from our scoping review synthesis will provide information that can be used to plan a program of work to rapidly evaluate VE in the event of future pandemics and other major healthcare challenges.

INTRODUCTION

The COVID-19 pandemic has been unprecedented in terms of its direct health impacts and disruption of many aspects of modern society. It has also been remarkable in the speed with which scientists and industry collaborated in the production and testing of a range of vaccines.

It became apparent quickly that the COVID-19 vaccines did not stimulate sterilising immunity but provided protection against severe illness and death, most importantly in those with underlying risk factors.(1, 2) The randomised trials that formed the evidence base for the initial deployment of vaccines included few subjects who were elderly, pregnant, had immunodeficiency or severe co-morbidity states.(3) Although quite large, the randomised trials documented few deaths and could not provide precise estimates of the effectiveness of the vaccines in reducing COVID-related and all-cause mortality.

The subsequent evaluation of vaccine effectiveness (VE) using controlled observational studies has been complicated by changes in the infectiousness and virulence of the SARS-CoV-2 virus, and rising background levels of vaccine-induced or naturally acquired immunity. Case fatality rates have fallen substantially, particularly in highly vaccinated countries.(4) Deaths are now concentrated in a group of older patients, those with obesity and those who have serious comorbidities or are immunocompromised.(5) This rapidly changing landscape created a need for continuous ‘real-world’ studies (RWS) of vaccine effectiveness in susceptible groups, against emerging viral variants and after repeated vaccine doses. These studies use routinely collected data to define exposures, endpoints and relevant covariates, analysing data from electronic medical records, administrative records, death registries and registries such as those set up specifically to record infection status and vaccine receipt.

Most VE studies of COVID-19 vaccines have employed large population-scale linked routinely collected datasets. However, countries have varied in the timeliness of their response to this major challenge. In some countries, for instance Israel and UK, collaborations between researchers, health service providers and government agencies enabled rapid analyses of large datasets using sophisticated techniques to adjust for confounding and other sources of bias. In contrast, other countries, for instance Australia and Aotearoa New Zealand, were slow to conduct effectiveness studies, in part because of low infection rates early in the pandemic, and also due to difficulties in accessing linked datasets.(6, 7)

Systematic reviews of VE studies have concentrated, appropriately, on the vaccines' ability to prevent serious illness and death.(8-11) They have been consistent in confirming that multiple doses of the available vaccines have been associated with large reductions in mortality, with quite rapid waning (over months) in protection, mandating a need for continued booster doses.

The COVID-19 pandemic has been a historic event that we must learn from. The rapid deployment of vaccines, followed by studies of their effectiveness, represents the largest and most important evaluated healthcare intervention in recent history. The sense of pandemic urgency led to rapid development of strategies to establish datasets, designs, and analytic approaches. This evolution of questions asked, data and resulting methods through the course of the pandemic used provides a unique learning opportunity for policy makers and researchers alike.

Therefore, we plan on conducting a scoping review of the evidence base on real world Covid-19 vaccine effectiveness to document this evolution; specifically, how policy-relevant questions changed over the course of the pandemic, how these affected the choices of data sources, designs, and analytical methods. By analysing these we hope to provide information that is useful to the following stakeholders:

1) Policy makers and health system managers: by indicating what datasets will have to be created de novo and the need for linkage to existing routinely collected data in responding to future pandemics.
2) Clinical and laboratory scientists: by identifying the disease manifestations and clinical and demographic vulnerability factors that will inform the designs and analyses of linked datasets needed to evaluate the effectiveness of vaccines and other interventions and how these may change over the course of a future pandemic and can advocate for the appropriate datasets to be linked and made available to researchers.

For peer review only

3) Data scientists and methodologists: to provide guidance as to study designs, analytical and adjustment techniques that are most used in providing rapid estimates of VE early in a future pandemic; to advocate for the data elements required to deal with confounding to be collected and available in a linked analysable form.
4) Vaccine manufacturers: to understand better the post licensing requirements for vaccines and pharmaceutical products under pandemic conditions and contribute appropriately to the necessary evaluations.
5) The pharmacoepidemiology community generally: the rapid evaluation of vaccine effectiveness during the COVID-19 pandemic provides lessons for the investigation of a range of pharmaceutical treatments for emerging health threats.

METHODS

We will conduct a scoping review, following the methods published by the Joanna Briggs Institute(12) and report the results according to the Preferred Reporting Items for Systematic Reviews and Meta-Analyses statement for scoping review (PRISMA-ScR).(13) This scoping review is registered with the Open Science Framework (OSF; https://doi.org/10.17605/OSF.IO/ZHDKR). As this scoping review will only include data in the public domain ethics review is not required.

INFORMATION SOURCES AND DATA SELECTION

We will use the Johns Hopkins Bloomberg School of Public Health VIEW-hub database (VIEW-hub) on vaccine COVID-19 effectiveness as our data source.(14) The search strategy and inclusion criteria have been described in detail by VIEW-hub curators and the database is updated weekly.(15) Broadly speaking the database includes both published and pre-print studies of vaccine effectiveness identified from PubMed, Embase, WHO COVID Database, Scopus, Web of Science, MMWR, Eurosurveillance, and medRxiv, bioRxiv, SSRN, Europe PMC, Research Square, and Knowledge Hub. To be included in the database studies must contain at least one vaccine effectiveness estimate and meet a minimal set of quality criteria (e.g. reports with adequate scientific detail, outcomes are laboratory confirmed, confirmed exposure to vaccine as opposed to use of recall, and use of contemporaneous control etc).(15) In addition, the database contains some pre-extracted data including study author, title, date published, link to paper, country of origin, vaccine studied, variant studied, population, study start and end date, and outcomes of interest. The VIEW-hub database has been used by researchers, regulators and policy makers.(16, 17)

The impacts of vaccines on infection and transmission have been limited and transient,(18) and diminishes the value of infection as the principal study endpoint. The decline in PCR

For peer review only

testing and registration of antigen test results have reduced the value of test results as the basis for test negative designs.(19, 20) The nature of COVID-19 related hospitalisations has changed during the pandemic with an increase in incidental findings of infection through routine testing of patients admitted for other reasons.(20) On the other hand, there has been an increasing focus on excess all-cause mortality as a measure of the success of countries in controlling the spread of the virus and mitigating its negative impacts on healthcare systems.(21, 22) We will therefore restrict our scoping review to all studies from the VIEW-hub database that examine mortality (coded as "death" in the database) as an outcome, either all cause or cause specific.

At the time of writing this protocol (1 Aug 2023) the VIEW-hub database(14) lists 495 observational studies of vaccine effectiveness from 50 countries, and 92 (~19%) list mortality as an endpoint.

DATA EXTRACTION

1) We will extract data on:
   Study characteristics: country, study design, publication status, protocol available, funding sources including whether the study was funded by an independent source or manufacturer, study ethics approval (or waiver), consent requirements (or waiver).Study context: reported vaccine policies in place, reported dominant viral variant at time of study.PICO-T: inclusion and exclusion criteria, exposure (i.e. vaccine(s)) and definition of exposed, control group, outcome definitions, outcomes collection period, time period of follow up and number of events in the dataset.
2) Data sources and additional variables: the types of data sources used (e.g., survey, electronic medical records, administrative data), which were linked at an individual level and which were not, baseline confounders collected, and for adjusted outcomes which variables they were adjusted for;
3) Analytic strategies to minimise bias: methods for minimising baseline confounding (e.g., propensity score analysis, instrumental variable analysis, covariate adjustment, self-controlled design etc) and further details of how the methods were implemented as appropriate, such as how the propensity score was implemented (matching, stratification or IPTW) which variables were included in the propensity score model. Additionally, sensitivity analysis conducted, subgroups analysed, methods used for dealing with missing data, and methods used for dealing with time varying environmental risk will be extracted.

We anticipate there will be a few data points where it will be difficult to provide an exhaustive list of potential categories for some of the variables of interest *a priori*. We will therefore take

For peer review only

an inductive approach to categorising variables such as “data sources”, “inclusion criteria”, and “adjustment techniques” by entering in free text and then developing categories through group discussion.

The lead author (PS) will develop a purpose-built data-extraction form developed in Microsoft Excel and a blank copy of the form will be provided on our OSF site. PS will also develop a validation set using a random sample of 7 papers and verified by experts in pharmacoepidemiology (DH) and analysis (XC). A single author (CD) will independently extract data on the validation set until 80% agreement is achieved, at which stage they will continue with data extraction. Where the data have already been pre-extracted by VIEW-hub database, entries will be checked for accuracy. A core team (DH, CD, PS, XC) will meet regularly to discuss each study and the main messages that it provides. The broader study team will meet less frequently to address issues arising and ensure data is categorised in a meaningful way that helps to inform decision making.

All data will be made publicly available via OSF.

ASSESSMENT OF RISK OF BIAS

We aim to describe the evolution of the literature and will therefore not conduct a formal assessment of the risk of bias (RoB) in the included studies. However, all included studies in the VIEW-hub database must meet a minimal set of quality criteria, and while this does not mean that they are free of bias, the process aims to ensure a baseline level of quality.

DATA SYNTHESIS

To describe the evolution of RWS of COVID-19 VE over the course of the pandemic, we will use descriptive statistics to quantify study characteristics – including evolution of study design (e.g. test-negative designs, cohorts, regression discontinuity), research questions asked (e.g.: comparisons done, effectiveness and waning effect, etc), data sources (e.g. regularly collected population data, registry data), analytic approaches (e.g. by design or form of adjustment), populations included, countries studied, how outcomes were defined, and event rates.

We will provide a temporal sequence of these characteristics overall, and where there are sufficient data within countries, and present them visually (e.g. as annotated stacked area graphs) to establish a template that enables anticipation of study questions and therefore plan for data availability in future pandemics.

For peer review only

We plan to develop interactive visuals as outputs so that stakeholders can interrogate the data further. All data manipulation, analysis and visualisation will occur using Python and R and we will share all code via OSF.

REVIEW TEAM AND CONSULTATION

Our review team consists of content experts in review methodology, vaccine and drug effectiveness studies, biostatistics and data science. Most of the team members are actively involved in the NHMRC-funded Medicines Intelligence Centre for Research Excellence that aims to accelerate real-world evidence development to inform medicines policy decision making.(23) Our reference group comprises end users in infectious diseases and pandemic management, vaccine epidemiology, and medicines and vaccine policy.

All authors and advisory group members have provided comment on this protocol, and the appropriateness of the research questions and data elements. The advisory group will be consulted on how best to present the data so that it is usable and helps with decision making in their respective areas.

In addition, we anticipate that the data we collect could be used for future review automation work that could improve the efficiency of research. Our advisory group also includes an expert in review methodology and automation who will provide advice on future-proofing our dataset.

DISSEMINATION

This scoping review results will be disseminated in five ways: 1) working papers for dissemination to policy makers in Australia; 2) open access publication of findings in peer reviewed journals; 3) presentation of findings at local and international infectious disease, vaccine, health systems and health management conferences. 4) online interactive visual to allow interrogation of the data; 5) open access to our data, code, and preprints via OSF.

For peer review only

REFERENCES

1. Polack FP, Thomas SJ, Kitchin N, Absalon J, Gurtman A, Lockhart S, et al. Safety and Efficacy of the BNT162b2 mRNA Covid-19 Vaccine. New England Journal of Medicine. 2020;383(27):2603-15.

2. Voysey M, Clemens SAC, Madhi SA, Weckx LY, Folegatti PM, Aley PK, et al. Safety and efficacy of the ChAdOx1 nCoV-19 vaccine (AZD1222) against SARS-CoV-2: an interim analysis of four randomised controlled trials in Brazil, South Africa, and the UK. The Lancet. 2021;397(10269):99-111.

3. Xiao H, Vaidya R, Liu F, Chang X, Xia X, Unger JM. Sex, Racial, and Ethnic Representation in COVID-19 Clinical Trials: A Systematic Review and Meta-analysis. JAMA Internal Medicine. 2023;183(1):50-60.

4. Haider N, Hasan MN, Guitian J, Khan RA, McCoy D, Ntoumi F, et al. The disproportionate case–fatality ratio of COVID-19 between countries with the highest vaccination rates and the rest of the world. IJID Regions. 2023;6:159-66.

5. Booth A, Reed AB, Ponzo S, Yassaee A, Aral M, Plans D, et al. Population risk factors for severe disease and mortality in COVID-19: A global systematic review and meta-analysis. PLOS ONE. 2021;16(3):e0247461.

6. Henry D, Stehlik P, Camacho X, Pearson SA. Access to routinely collected data for population health research: experiences in Canada and Australia. Australian and New Zealand Journal of Public Health. 2018;42(5):430-3.

7. Henry DA, Jones MA, Stehlik P, Glasziou PP. Effectiveness of COVID-19 vaccines: findings from real world studies. Medical Journal of Australia. 2021;215(4):149-51.e1.

8. Huang Z, Xu S, Liu J, Wu L, Qiu J, Wang N, et al. Effectiveness of inactivated and Ad5-nCoV COVID-19 vaccines against SARS-CoV-2 Omicron BA. 2 variant infection, severe illness, and death. BMC Medicine. 2022;20(1):400.

9. Prasad S, Kalafat E, Blakeway H, Townsend R, O'Brien P, Morris E, et al. Systematic review and meta-analysis of the effectiveness and perinatal outcomes of COVID-19 vaccination in pregnancy. Nature Communications. 2022;13(1):2414.

10. Rahmani K, Shavaleh R, Forouhi M, Disfani HF, Kamandi M, Oskooi RK, et al. The effectiveness of COVID-19 vaccines in reducing the incidence, hospitalization, and mortality from COVID-19: A systematic review and meta-analysis. Frontiers in Public Health. 2022;10.

11. Kow CS, Hasan SS. Real-world effectiveness of BNT162b2 mRNA vaccine: a meta-analysis of large observational studies. Inflammopharmacology. 2021;29(4):1075-90.

12. DJ Peters M, Godfrey C, McInerney P, Munn Z, Tricco AC, Khalil H. Chapter 11: Scoping Reviews. 2020. In: JBI Manual for Evidence Synthesis [Internet]. https://synthesismanual.jbi.global: JBI.

13. Tricco AC, Lillie E, Zarin W, O'Brien KK, Colquhoun H, Levac D, et al. PRISMA Extension for Scoping Reviews (PRISMA-ScR): Checklist and Explanation. Annals of Internal Medicine. 2018;169(7):467-73.

14. International Vaccine Access Center (IVAC), Johns Hopkins Bloomberg School of Public Health. VIEW-hub. 2023.

15. International Vaccine Access Center, Johns Hopkins Bloomberg School of Public Health, World Health Organization, Coalition for Epidemic Preparedness Innovation. Results of COVID-19 Vaccine Effectiveness & Impact Studies: An Ongoing Systematic Review. Methods. https://view-hub.org/sites/default/files/2022-

For peer review only

09/COVID19_VE_Lit_Review_Methods.pdf: International Vaccine Access Center (IVAC), Johns Hopkins Bloomberg School of Public Health. VIEW-hub.,; 2022 September 2022.

16. National Center for Immunization and Respiratory Diseases (NCIRD) - Division of Viral Diseases. Science Brief: COVID-19 Vaccines and Vaccination USA: Centers for Disease Control and Prevention; 2021 [updated 15 Sept 2021. Available from: https://www.cdc.gov/coronavirus/2019-ncov/science/science-briefs/fully-vaccinated-people.html.

17. Feikin DR, Higdon MM, Abu-Raddad LJ, Andrews N, Araos R, Goldberg Y, et al. Duration of effectiveness of vaccines against SARS-CoV-2 infection and COVID-19 disease: results of a systematic review and meta-regression. The Lancet. 2022;399(10328):924-44.

18. Tan ST, Kwan AT, Rodríguez-Barraquer I, Singer BJ, Park HJ, Lewnard JA, et al. Infectiousness of SARS-CoV-2 breakthrough infections and reinfections during the Omicron wave. Nature Medicine. 2023;29(2):358-65.

19. Shi X, Li KQ, Mukherjee B. Current Challenges With the Use of Test-Negative Designs for Modeling COVID-19 Vaccination and Outcomes. American Journal of Epidemiology. 2023;192(3):328-33.

20. UK Health Security Agency working with the Medicines & Healthcare products Regulatory Agency. COVID-19 vaccine surveillance report (2 March 2023). In: Care DoHaS, editor. gov.uk2023.

21. Mukherjee B. Being a Public Health Statistician During a Global Pandemic. Statistical Science. 2022;37(2):270-7, 8.

22. The true death toll of COVID-19: estimating global excess mortality: World Health Organization; [cited 2023 March 7]. Available from: https://www.who.int/data/stories/the-true-death-toll-of-covid-19-estimating-global-excess-mortality.

23. Pratt N, Camacho X, Vajdic C, Degenhardt L, Laba T-L, Hillen J, et al. The Medicines Intelligence Centre of Research Excellence: Co-creating real-world evidence to support the evidentiary needs of Australian medicines regulators and payers. International Journal of Population Data Science. 2021;6(3).

For peer review only

Preferred Reporting Items for Systematic reviews and Meta-Analyses extension for Scoping Reviews (PRISMA-ScR) Checklist

SECTION	ITEM	PRISMA-ScR CHECKLIST ITEM	REPORTED ON PAGE #
**TITLE**			
Title	1	Identify the report as a scoping review.	1
**ABSTRACT**			
Structured summary	2	Provide a structured summary that includes (as applicable): background, objectives, eligibility criteria, sources of evidence, charting methods, results, and conclusions that relate to the review questions and objectives.	3
**INTRODUCTION**			
Rationale	3	Describe the rationale for the review in the context of what is already known. Explain why the review questions/objectives lend themselves to a scoping review approach.	5
Objectives	4	Provide an explicit statement of the questions and objectives being addressed with reference to their key elements (e.g., population or participants, concepts, and context) or other relevant key elements used to conceptualize the review questions and/or objectives.	5
**METHODS**			
Protocol and registration	5	Indicate whether a review protocol exists; state if and where it can be accessed (e.g., a Web address); and if available, provide registration information, including the registration number.	6
Eligibility criteria	6	Specify characteristics of the sources of evidence used as eligibility criteria (e.g., years considered, language, and publication status), and provide a rationale.	6
Information sources*	7	Describe all information sources in the search (e.g., databases with dates of coverage and contact with authors to identify additional sources), as well as the date the most recent search was executed.	6
Search	8	Present the full electronic search strategy for at least 1 database, including any limits used, such that it could be repeated.	NA
Selection of sources of evidence†	9	State the process for selecting sources of evidence (i.e., screening and eligibility) included in the scoping review.	6-7
Data charting process‡	10	Describe the methods of charting data from the included sources of evidence (e.g., calibrated forms or forms that have been tested by the team before their use, and whether data charting was done independently or in duplicate) and any processes for obtaining and confirming data from investigators.	7-8
Data items	11	List and define all variables for which data were sought and any assumptions and simplifications made.	7-8
Critical appraisal of individual sources of evidence§	12	If done, provide a rationale for conducting a critical appraisal of included sources of evidence; describe the methods used and how this information was used in any data synthesis (if appropriate).	8
Synthesis of results	13	Describe the methods of handling and summarizing the data that were charted.	8

St. Michael's
Inspired Care.
Inspiring Science.

For peer review only - http://bmjopen.bmj.com/site/about/guidelines.xhtml

SECTION	ITEM	PRISMA-ScR CHECKLIST ITEM	REPORTED ON PAGE #
**RESULTS**			
Selection of sources of evidence	14	Give numbers of sources of evidence screened, assessed for eligibility, and included in the review, with reasons for exclusions at each stage, ideally using a flow diagram.	TBC
Characteristics of sources of evidence	15	For each source of evidence, present characteristics for which data were charted and provide the citations.	TBC
Critical appraisal within sources of evidence	16	If done, present data on critical appraisal of included sources of evidence (see item 12).	TBC
Results of individual sources of evidence	17	For each included source of evidence, present the relevant data that were charted that relate to the review questions and objectives.	TBC
Synthesis of results	18	Summarize and/or present the charting results as they relate to the review questions and objectives.	TBC
**DISCUSSION**			
Summary of evidence	19	Summarize the main results (including an overview of concepts, themes, and types of evidence available), link to the review questions and objectives, and consider the relevance to key groups.	TBC
Limitations	20	Discuss the limitations of the scoping review process.	TBC
Conclusions	21	Provide a general interpretation of the results with respect to the review questions and objectives, as well as potential implications and/or next steps.	TBC
**FUNDING**			
Funding	22	Describe sources of funding for the included sources of evidence, as well as sources of funding for the scoping review. Describe the role of the funders of the scoping review.	2 (research members only)

JBI = Joanna Briggs Institute; PRISMA-ScR = Preferred Reporting Items for Systematic reviews and Meta-Analyses extension for Scoping Reviews.
* Where *sources of evidence* (see second footnote) are compiled from, such as bibliographic databases, social media platforms, and Web sites.
† A more inclusive/heterogeneous term used to account for the different types of evidence or data sources (e.g., quantitative and/or qualitative research, expert opinion, and policy documents) that may be eligible in a scoping review as opposed to only studies. This is not to be confused with *information sources* (see first footnote).
‡ The frameworks by Arksey and O'Malley (6) and Levac and colleagues (7) and the JBI guidance (4, 5) refer to the process of data extraction in a scoping review as data charting.
§ The process of systematically examining research evidence to assess its validity, results, and relevance before using it to inform a decision. This term is used for items 12 and 19 instead of "risk of bias" (which is more applicable to systematic reviews of interventions) to include and acknowledge the various sources of evidence that may be used in a scoping review (e.g., quantitative and/or qualitative research, expert opinion, and policy document).

From: Tricco AC, Lillie E, Zarin W, O'Brien KK, Colquhoun H, Levac D, et al. PRISMA Extension for Scoping Reviews (PRISMAScR): Checklist and Explanation. Ann Intern Med. 2018;169:467–473. doi: 10.7326/M18-0850.

For peer review only - http://bmjopen.bmj.com/site/about/guidelines.xhtml

BMJ Open

EVOLution of the data and methods in real world COVID-19 Vaccine Effectiveness studies on mortality: A Scoping Review Protocol

Journal:	*BMJ Open*
Manuscript ID	bmjopen-2023-079071.R1
Article Type:	Protocol
Date Submitted by the Author:	16-Nov-2023
Complete List of Authors:	Stehlik, Paulina; Griffith University Faculty of Health, School of Pharmacy and Medical Sciences; Bond University Faculty of Health Sciences and Medicine, Institute for Evidence-Based Healthcare<br>Dowsett, Caroline; Griffith University Faculty of Health, School of Pharmacy and Medical Sciences<br>Camacho, Ximena; University of New South Wales Medicine & Health, School of Population Health<br>Falster, Michael; University of New South Wales Medicine & Health, School of Population Health<br>Lim, Renly; University of South Australia Division of Health Sciences, Quality Use of Medicines and Pharmacy Research Centre<br>Nasreen, Sharifa; University of Toronto,<br>Pratt, Nicole; University of South Australia Clinical & Health Sciences Academic Unit, Quality Use of Medicines and Pharmacy Research Centre<br>Pearson, Sallie-Anne; University of New South Wales, School of Population Health<br>Henry, David; Bond University Faculty of Health Sciences and Medicine, Institute for Evidence-Based Healthcare; University of New South Wales, School of Population Health
**Primary Subject Heading**:	Infectious diseases
Secondary Subject Heading:	Epidemiology, Research methods, Public health
Keywords:	COVID-19, STATISTICS & RESEARCH METHODS, Protocols & guidelines < HEALTH SERVICES ADMINISTRATION & MANAGEMENT, INFECTIOUS DISEASES, Mortality, PUBLIC HEALTH

SCHOLARONE™
Manuscripts

EVOLution of the data and methods in real world COVID-19 Vaccine Effectiveness studies on mortality: A Scoping Review Protocol

Short title: EVOLVE ScR

FORWARD

AUTHORS

Paulina Stehlik, School of Pharmacy and Medical Sciences, Griffith University; Adjunct Faculty of Health Sciences and Medicine, Bond University*

Caroline Dowsett, School of Pharmacy and Medical Sciences, Griffith University.

Ximena Camacho, School of Population Health, Faculty of Medicine and Health, University of New South Wales

Michael Falster, School of Population Health, Faculty of Medicine and Health, University of New South Wales

Renly Lim, Quality Use of Medicines and Pharmacy Research Centre, UniSA Clinical and Health Sciences, University of South Australia

Sharifa Nasreen, Epidemiology and Biostatistics, School of Public Health, SUNY Downstate Health Sciences University

Nicole Pratt, Quality Use of Medicines and Pharmacy Research Centre, UniSA Clinical and Health Sciences, University of South Australia

Sallie-Anne Pearson, School of Population Health, Faculty of Medicine and Health, University of New South Wales

David Henry, Institute for Evidence-Based Healthcare, Bond University and School of Population Health, Faculty of Medicine and Health, University of New South Wales

CORRESPONDING AUTHOR

For peer review only

Dr Paulina Stehlik, School of Pharmacy and Medical Sciences, Griffith University; Adjunct Faculty of Health Sciences and Medicine, Bond University

Email: p.stehlik@griffith.edu.au

ADVISORY GROUP

Bette Liu, UNSW National Centre for Immunisation Research and Surveillance

Andrew Wilson, PBAC advisory committee chair; Director of the Menzies Centre for Health Policy, Menzies Centre for Health Policy, School of Public Health

Kylie Alcorn, A/Director Infectious Diseases, Infectious Disease Department, Gold Coast Health

Alexandra Bannach-Brown, Charité - Universitätsmedizin Berlin

ABSTRACT

Background: Early evidence on COVID-19 vaccine efficacy came from randomised trials. However, many important questions about vaccine effectiveness (VE) in vulnerable groups and evolving viral variants have been addressed using real-world data. The results of these studies have informed most vaccination policies globally. As the questions about VE have evolved during the pandemic so have data, study design and analytical choices. This scoping review aims to characterise this evolution and provide insights for future pandemic planning – specifically, what kinds of questions are asked at different stages of a pandemic, and what data infrastructure and methods are used to answer these questions?

Methods and analysis: We will identify relevant studies in the Johns Hopkins Bloomberg School of Public Health VIEW-hub database. We will include real-world studies of COVID-19 VE that reported COVID-19-specific and all-cause mortality. We will extract information on study characteristics; study context; data sources; design and analytic methods that deal with confounding.

A single reviewer will extract data after achieving 80% agreement on a validation set. Variables such as data sources will be categorised using an inductive approach and each study will be discussed in a small group setting. A timeline mapping approach will be used to capture the evolution of this body of literature. Within-country activities will be documented to discern the development of data design and analytic strategies.

Dissemination: This review will provide important information on how study questions, data availability and resulting design choices of VE studies evolved through the COVID-19 pandemic. This review will help identify options for planning and VE studies and inform policy makers on the minimal data and analytic infrastructure needed to support rapid real-

For peer review only

world evaluation of VE in future pandemics. The findings will also be relevant to initiatives to rapidly evaluate effectiveness of health care strategies more broadly.

Registration: https://doi.org/10.17605/OSF.IO/ZHDKR

For peer review only

STRENGTHS AND LIMITATIONS

- We will use a curated database (Johns Hopkins Bloomberg School of Public Health VIEW-hub) to identify studies for inclusion in a scoping review of the design methods and data choices used to conduct real world studies of the effectiveness of COVID-19 vaccines in preventing mortality.
- VIEW-hub is a comprehensive database compiled from weekly searches of the literature across multiple databases, preprint servers and the grey literature and defines relevant studies based on a set of quality criteria.
- While use of a curated database may lead to some studies being missed, this is unlikely to change the overall findings of this scoping review
- Data will be categorised to facilitate data synthesis and mapping. To allow for data exploration we will produce interactive visuals that enable researchers and policy makers to explore the data independently, and we will make our data open access at the conclusion of our study.

For peer review only

INTRODUCTION

The COVID-19 pandemic has been unprecedented in terms of its direct health impacts and disruption of many aspects of modern society. It has also been remarkable in the speed with which scientists and industry collaborated in the production and testing of a range of vaccines.

It became apparent quickly that the COVID-19 vaccines did not stimulate sterilising immunity but provided protection against severe illness and death, most importantly in those with underlying risk factors.(1, 2) The randomised trials that formed the evidence base for the initial deployment of vaccines included few subjects who were elderly, pregnant, had immunodeficiency or severe co-morbidity states.(3) Although quite large, the randomised trials documented few deaths and could not provide precise estimates of the effectiveness of the vaccines in reducing COVID-related and all-cause mortality.

The subsequent evaluation of vaccine effectiveness (VE) using controlled observational studies has been complicated by changes in the infectiousness and virulence of the SARS-CoV-2 virus, and rising background levels of vaccine-induced or naturally acquired immunity. Case fatality rates have fallen substantially, particularly in highly vaccinated countries.(4) Deaths are now concentrated in a group of older patients, those with obesity and those who have serious comorbidities or are immunocompromised.(5) This rapidly changing landscape created a need for continuous 'real-world' studies (RWS) of vaccine effectiveness in susceptible groups, against emerging viral variants and after repeated vaccine doses.(6) These studies use data collected outside of a clinical trials setting to define exposures, endpoints and relevant covariates. This is achieved by analysing data from electronic medical records, administrative records, death registries and registries established specifically to record infection status and vaccine receipt.(6)

Most VE studies of COVID-19 vaccines have employed large population-scale linked routinely collected datasets. However, countries have varied in the timeliness of their response to this major challenge. In some countries, for instance Israel and UK, collaborations between researchers, health service providers and government agencies enabled rapid analyses of large datasets using sophisticated techniques to adjust for confounding and other sources of bias. In contrast, other countries, for instance Australia and Aotearoa New Zealand, were slow to conduct effectiveness studies, in part because of low infection rates early in the pandemic, and in Australia because of difficulties in accessing the necessary linked datasets.(7, 8)

Systematic reviews of VE studies have concentrated, appropriately, on the vaccines' ability to prevent serious illness and death.(9-12) They have been consistent in confirming that multiple doses of the available vaccines have been associated with large reductions in mortality, with quite rapid waning (over months) in protection, mandating a need for continued booster doses.

For peer review only

The COVID-19 pandemic has been a historic event that we must learn from. The rapid deployment of vaccines, followed by studies of their effectiveness, represents the largest and most important evaluated healthcare intervention in recent history. The sense of pandemic urgency led to rapid development of strategies to establish datasets, designs, and analytic approaches. This evolution of questions asked, data and resulting methods through the course of the pandemic used provides a unique learning opportunity for policy makers and researchers alike.

We plan to conduct a scoping review of the evidence base on real world Covid-19 vaccine effectiveness to document this evolution; specifically, how policy-relevant questions changed over the course of the pandemic, how these affected the choices of data sources, designs, and analytical methods. By analysing these we hope to provide information that is useful to the following stakeholders:

1. Policy makers and health system managers: by indicating what datasets will have to be created de novo and the need for linkage to existing routinely collected data in responding to future pandemics.
2. Clinicians and laboratory scientists: by identifying the disease manifestations and clinical and demographic vulnerability factors that will inform the designs and analyses of linked datasets needed to evaluate the effectiveness of vaccines and other interventions and how these may change over the course of a future pandemic and can advocate for the appropriate datasets to be linked and made available to researchers.
3. Data scientists and methodologists: to provide guidance as to study designs, analytical and adjustment techniques that are most often used in providing rapid estimates of VE early in a future pandemic; to advocate for the data elements required to deal with confounding to be collected and available in a linked analysable form.
4. Vaccine manufacturers: to understand better the post licensing requirements for vaccines and pharmaceutical products under pandemic conditions and contribute appropriately to the necessary evaluations.
5. The pharmacoepidemiology community generally: the rapid evaluation of vaccine effectiveness during the COVID-19 pandemic provides lessons for the timely investigation of a range of pharmaceutical treatments for emerging health threats.

METHODS

We will conduct a scoping review, following the methods published by the Joanna Briggs Institute(13) and report the results according to the Preferred Reporting Items for Systematic Reviews and Meta-Analyses statement for scoping review (PRISMA-ScR).(14) This scoping review is registered with the Open Science Framework (OSF; https://doi.org/10.17605/OSF.IO/ZHDKR). Data extraction has begun (25th September 2023, after protocol registration, and will continue for approximately 6 months).

For peer review only

INFORMATION SOURCES AND DATA SELECTION

To be included in our review studies must provide outcome measures of COVID-19 vaccine effectiveness on mortality, and a study design that has a contemporaneous control group and uses RWD. Studies can be published or pre-prints. As our aim is to describe the evolution of vaccine effectiveness studies using RWD we will exclude randomised trials, modelling studies, reviews, and studies without a contemporaneous control such as case series or self-controlled design. We will use the Johns Hopkins Bloomberg School of Public Health VIEW-hub database (VIEW-hub) on vaccine COVID-19 effectiveness studies as our data source as the search strategy and inclusion criteria meet the requirements of our review.(15)

The VIEW-hub was established in 2016 as a go-to resource for researchers, decision makers and funders, policy makers and advocates for reliable vaccine information. Since early 2021 it conducted systematic searches of studies of COVID-19 vaccine effectiveness on a weekly basis, and has been used by researchers, regulators and policy makers to evaluate COVID-19 vaccine effectiveness previously.(16, 17)

The VIEW-hub search strategy and inclusion criteria have been described in detail by VIEW-hub curators and the database is updated weekly.(18) Broadly speaking the database includes both published and pre-print studies of vaccine effectiveness identified from PubMed, Embase, Scopus, Web of Science, the WHO COVID Database, MMWR, Eurosurveillance, and medRxiv, bioRxiv, SSRN, Europe PMC, Research Square, and Knowledge Hub, as well as Google alerts for COVID-19 vaccine effectiveness studies. A detailed description of the search strategy and inclusion criteria are provided in the VEIW-hub methods paper.(18) To be included in the VIEW-Hub database studies must include at least one vaccine effectiveness estimate and meet a minimal set of quality criteria (e.g. studies must have a contemporaneous control, COVID-19 must be confirmed through PCR or antigen test, vaccination status cannot be established via recall, and studies must have no significant bias that likely affects results).(18) Studies are screened weekly by the same two epidemiologists, who also extract some data about included studies. These include study author, title, date published, link to paper, country of origin, vaccine studied, variant studied, population, study start and end date, and outcomes of interest.

The impacts of vaccines on infection and transmission have been limited and transient,(19) and diminishes the value of infection as the principal study endpoint. The decline in PCR testing and registration of antigen test results have reduced the value of test results as the basis for test negative designs.(20, 21) The nature of COVID-19 related hospitalisations has changed during the pandemic with an increase in incidental findings of infection through routine testing of patients admitted for other reasons.(21) On the other hand, there has been an increasing focus on excess all-cause mortality as a measure of the success of countries in controlling the spread of the virus and mitigating its negative impacts on healthcare systems.(22, 23) We will therefore restrict our scoping review to all studies from

For peer review only

the VIEW-hub database that examine mortality (coded as “death” in the database) as an outcome, either all cause or cause specific.

At the time of writing this protocol (1 Aug 2023) the VIEW-hub database(15) lists 495 observational studies of vaccine effectiveness from 50 countries, and 92 (~19%) list mortality as an endpoint.

DATA EXTRACTION

We will extract data on:

1. *Study characteristics*: country, study design, publication status, protocol available, funding sources including whether the study was funded by an independent source or manufacturer, study ethics approval (or waiver), consent requirements (or waiver).
2. *Study context*: reported vaccine policies in place, reported dominant viral variant at time of study.
3. *PICO-T*: inclusion and exclusion criteria, exposure (i.e. vaccine(s)) and definition of exposed, control group, outcome definitions, outcomes collection period, time period of follow up and number of events in the dataset.
4. *Data sources and additional variables*: the types of data sources used (e.g., survey, electronic medical records, administrative data), which were linked at an individual level and which were not, baseline confounders collected, and for adjusted outcomes which variables they were adjusted for;
5. *Analytic strategies to minimise bias*: methods for minimising baseline confounding (e.g., propensity score analysis, instrumental variable analysis, covariate adjustment, self-controlled design etc) and further details of how the methods were implemented as appropriate, such as how the propensity score was implemented (matching, stratification or IPTW) which variables were included in the propensity score model. Additionally, sensitivity analysis conducted, subgroups analysed, methods used for dealing with missing data, and methods used for dealing with time varying environmental risk will be extracted.

We anticipate there will be a few data points where it will be difficult to provide an exhaustive list of potential categories for some of the variables of interest *a priori*. We will therefore take an inductive approach to categorising variables such as “data sources”, “inclusion criteria”, and “adjustment techniques” by entering in free text and then developing categories through group discussion.

The lead author (PS) will develop a purpose-built data-extraction form developed in SharePoint Lists and a blank copy of the form and data dictionary will be provided on our OSF site. PS will also develop a validation set using a random sample of 7 papers and verified by experts in pharmacoepidemiology (DH) and analysis (XC). A single author (CD) will independently extract data on the validation set until 80% agreement is achieved, at which

For peer review only

stage they will continue with data extraction. A second reviewer (PS) will check the accuracy of all data extractions, and a core team (DH, CD, PS, XC) will meet regularly to discuss each study, whether it meets the inclusion criteria, and the main messages that it provides. The broader study team will meet less frequently to address issues arising and ensure data is categorised in a meaningful way that helps to inform decision making.

All data will be made publicly available via our study's OSF page (https://osf.io/m4cbf/).

ASSESSMENT OF RISK OF BIAS

We aim to describe the evolution of the literature and will therefore not conduct a formal assessment of the risk of bias in the included studies. However, all included studies in the VIEW-hub database must meet a minimal set of quality criteria, and while this does not mean that they are free of bias, the process aims to ensure a baseline level of quality.

DATA SYNTHESIS

To describe the evolution of RWS of COVID-19 VE over the course of the pandemic, we will use descriptive statistics to quantify study characteristics – including evolution of study design (e.g. test-negative designs, cohorts, regression discontinuity), research questions asked (e.g.: comparisons done, effectiveness and waning effect, etc), data sources (e.g. regularly collected population data, registry data), analytic approaches (e.g. by design or form of adjustment), populations included, countries studied, how outcomes were defined, and event rates.

We will provide a temporal sequence of these characteristics overall, and where there are sufficient data within countries, and present them visually (e.g. as annotated stacked area graphs) to establish a template that enables anticipation of study questions and therefore plan for data availability in future pandemics.

We plan to develop interactive visuals as outputs so that stakeholders can interrogate the data further. All data manipulation, analysis and visualisation will occur using Python and R and we will share all code via OSF.

REVIEW TEAM AND CONSULTATION

Our review team consists of content experts in review methodology, vaccine and drug effectiveness studies, biostatistics and data science. Most of the team members are actively involved in the NHMRC-funded Medicines Intelligence Centre for Research Excellence that aims to accelerate real-world evidence development to inform medicines policy decision making.(24) Our reference group comprises end users in infectious diseases and pandemic management, vaccine epidemiology, and medicines and vaccine policy.

All authors and advisory group members have provided comment on this protocol, and the appropriateness of the research questions and data elements. The advisory group will be

For peer review only

consulted on how best to present the data so that it is usable and helps with decision making in their respective areas.

In addition, we anticipate that the data we collect could be used for future review automation work that could improve the efficiency of research. Our advisory group also includes an expert in review methodology and automation who will provide advice on future-proofing our dataset.

ETHICS AND DISSEMINATION

As this scoping review will only include data in the public domain ethics review is not required.

Findings of this review will be relevant to several stakeholders, including those involved in pandemic response, data infrastructure and health technology evaluation. As such we will disseminate our findings in five ways: 1) working papers for dissemination to policy makers in Australia; 2) open access publication of findings in peer reviewed journals; 3) presentation of findings at local and international infectious disease, vaccine, health systems and health management conferences. 4) online interactive visual to allow interrogation of the data; 5) open access to our data, code, and preprints via OSF.

PATIENT AND PUBLIC INVOLVEMENT

None.

CONTRIBUTION (CREDIT AUTHOR STATEMENT)

Paulina Stehlik and David Henry conceptualised the project, acquired the funding, and are acting as project supervisors. Paulina Stehlik, Caroline Dowsett, Ximena Camacho, Michael Falster, Renly Lim, Sharifa Nasreen, Nicole Pratt, Sallie-Anne Pearson, and David Henry contributed to the methodology. Paulina Stehlik developed the resources and database, and oversees database and project management. Paulina Stehlik, David Henry and Ximena Camacho piloted the database and extraction tool and developed the validation set. Caroline Dowsette is conducting the data extraction while will be checked by Paulina Stehlik. Paulina Stehlik, Ximena Camacho and Michael Falster developed the data synthesis plan. Paulina Stehlik and David Henry wrote the original draft of this manuscript, and Paulina Stehlik, Caroline Dowsett, Ximena Camacho, Michael Falster, Renly Lim, Sharifa Nasreen, Nicole Pratt, Sallie-Anne Pearson, and David Henry all edited and reviewed the draft and final revisions.

AKNOWLEDGEMENTS

We would like to acknowledge the advisory group and Oyungerel Byambasuren to their feedback and comments on draft versions of this document, particularly on the

For peer review only - http://bmjopen.bmj.com/site/about/guidelines.xhtml

methodology and which variables to collect to provide meaningful information to decision makers.

FUNDING

Medicines Intelligence Centre for Research Excellence (MI-CRE) 2022 Project Incubator Grant; The MI-CRE is supported by the National Health and Medical Research Council's Centres of Research Excellence (CRE) scheme (ID 1196900)

Renly Lim is supported by a National Health and Medical Research Council (NHMRC) Early Career Fellowship (APP1156368).

Michael Falster is supported by a Future Leader Fellowship from the National Heart Foundation of Australia (ID: 105609).

Ximena Camacho is supported by a NHMRC Postgraduate Scholarship (ID: 2005259).

CONFLICTS OF INTEREST

No additional conflicts of interest to declare.

For peer review only

REFERENCES

1. Polack FP, Thomas SJ, Kitchin N, Absalon J, Gurtman A, Lockhart S, et al. Safety and Efficacy of the BNT162b2 mRNA Covid-19 Vaccine. New England Journal of Medicine. 2020;383(27):2603-15.

2. Voysey M, Clemens SAC, Madhi SA, Weckx LY, Folegatti PM, Aley PK, et al. Safety and efficacy of the ChAdOx1 nCoV-19 vaccine (AZD1222) against SARS-CoV-2: an interim analysis of four randomised controlled trials in Brazil, South Africa, and the UK. The Lancet. 2021;397(10269):99-111.

3. Xiao H, Vaidya R, Liu F, Chang X, Xia X, Unger JM. Sex, Racial, and Ethnic Representation in COVID-19 Clinical Trials: A Systematic Review and Meta-analysis. JAMA Internal Medicine. 2023;183(1):50-60.

4. Haider N, Hasan MN, Guitian J, Khan RA, McCoy D, Ntoumi F, et al. The disproportionate case–fatality ratio of COVID-19 between countries with the highest vaccination rates and the rest of the world. IJID Regions. 2023;6:159-66.

5. Booth A, Reed AB, Ponzo S, Yassaee A, Aral M, Plans D, et al. Population risk factors for severe disease and mortality in COVID-19: A global systematic review and meta-analysis. PLOS ONE. 2021;16(3):e0247461.

6. Swift B, Jain L, White C, Chandrasekaran V, Bhandari A, Hughes DA, et al. Innovation at the Intersection of Clinical Trials and Real-World Data Science to Advance Patient Care. Clinical and Translational Science. 2018;11(5):450-60.

7. Henry D, Stehlik P, Camacho X, Pearson SA. Access to routinely collected data for population health research: experiences in Canada and Australia. Australian and New Zealand Journal of Public Health. 2018;42(5):430-3.

8. Henry DA, Jones MA, Stehlik P, Glasziou PP. Effectiveness of COVID-19 vaccines: findings from real world studies. Medical Journal of Australia. 2021;215(4):149-51.e1.

9. Huang Z, Xu S, Liu J, Wu L, Qiu J, Wang N, et al. Effectiveness of inactivated and Ad5-nCoV COVID-19 vaccines against SARS-CoV-2 Omicron BA. 2 variant infection, severe illness, and death. BMC Medicine. 2022;20(1):400.

10. Prasad S, Kalafat E, Blakeway H, Townsend R, O'Brien P, Morris E, et al. Systematic review and meta-analysis of the effectiveness and perinatal outcomes of COVID-19 vaccination in pregnancy. Nature Communications. 2022;13(1):2414.

11. Rahmani K, Shavaleh R, Forouhi M, Disfani HF, Kamandi M, Oskooi RK, et al. The effectiveness of COVID-19 vaccines in reducing the incidence, hospitalization, and mortality from COVID-19: A systematic review and meta-analysis. Frontiers in Public Health. 2022;10.

For peer review only

12. Kow CS, Hasan SS. Real-world effectiveness of BNT162b2 mRNA vaccine: a meta-analysis of large observational studies. Inflammopharmacology. 2021;29(4):1075-90.

13. DJ Peters M, Godfrey C, McInerney P, Munn Z, Tricco AC, Khalil H. Chapter 11: Scoping Reviews. 2020. In: JBI Manual for Evidence Synthesis [Internet]. https://synthesismanual.jbi.global: JBI.

14. Tricco AC, Lillie E, Zarin W, O'Brien KK, Colquhoun H, Levac D, et al. PRISMA Extension for Scoping Reviews (PRISMA-ScR): Checklist and Explanation. Annals of Internal Medicine. 2018;169(7):467-73.

15. International Vaccine Access Center (IVAC), Johns Hopkins Bloomberg School of Public Health. VIEW-hub. 2023.

16. National Center for Immunization and Respiratory Diseases (NCIRD) - Division of Viral Diseases. Science Brief: COVID-19 Vaccines and Vaccination USA: Centers for Disease Control and Prevention; 2021 [updated 15 Sept 2021. Available from: https://www.cdc.gov/coronavirus/2019-ncov/science/science-briefs/fully-vaccinated-people.html.

17. Feikin DR, Higdon MM, Abu-Raddad LJ, Andrews N, Araos R, Goldberg Y, et al. Duration of effectiveness of vaccines against SARS-CoV-2 infection and COVID-19 disease: results of a systematic review and meta-regression. The Lancet. 2022;399(10328):924-44.

18. International Vaccine Access Center, Johns Hopkins Bloomberg School of Public Health, World Health Organization, Coalition for Epidemic Preparedness Innovation. Results of COVID-19 Vaccine Effectiveness & Impact Studies: An Ongoing Systematic Review. Methods. https://view-hub.org/sites/default/files/2022-09/COVID19_VE_Lit_Review_Methods.pdf: International Vaccine Access Center (IVAC), Johns Hopkins Bloomberg School of Public Health. VIEW-hub.,; 2022 September 2022.

19. Tan ST, Kwan AT, Rodríguez-Barraquer I, Singer BJ, Park HJ, Lewnard JA, et al. Infectiousness of SARS-CoV-2 breakthrough infections and reinfections during the Omicron wave. Nature Medicine. 2023;29(2):358-65.

20. Shi X, Li KQ, Mukherjee B. Current Challenges With the Use of Test-Negative Designs for Modeling COVID-19 Vaccination and Outcomes. American Journal of Epidemiology. 2023;192(3):328-33.

21. UK Health Security Agency working with the Medicines & Healthcare products Regulatory Agency. COVID-19 vaccine surveillance report (2 March 2023). In: Care DoHaS, editor. gov.uk2023.

22. Mukherjee B. Being a Public Health Statistician During a Global Pandemic. Statistical Science. 2022;37(2):270-7, 8.

For peer review only

23. The true death toll of COVID-19: estimating global excess mortality: World Health Organization; [cited 2023 March 7]. Available from: https://www.who.int/data/stories/the-true-death-toll-of-covid-19-estimating-global-excess-mortality.

24. Pratt N, Camacho X, Vajdic C, Degenhardt L, Laba T-L, Hillen J, et al. The Medicines Intelligence Centre of Research Excellence: Co-creating real-world evidence to support the evidentiary needs of Australian medicines regulators and payers. International Journal of Population Data Science. 2021;6(3).

For peer review only

Preferred Reporting Items for Systematic reviews and Meta-Analyses extension for Scoping Reviews (PRISMA-ScR) Checklist

SECTION	ITEM	PRISMA-ScR CHECKLIST ITEM	REPORTED ON PAGE #
**TITLE**			
Title	1	Identify the report as a scoping review.	1
**ABSTRACT**			
Structured summary	2	Provide a structured summary that includes (as applicable): background, objectives, eligibility criteria, sources of evidence, charting methods, results, and conclusions that relate to the review questions and objectives.	3
**INTRODUCTION**			
Rationale	3	Describe the rationale for the review in the context of what is already known. Explain why the review questions/objectives lend themselves to a scoping review approach.	5
Objectives	4	Provide an explicit statement of the questions and objectives being addressed with reference to their key elements (e.g., population or participants, concepts, and context) or other relevant key elements used to conceptualize the review questions and/or objectives.	5
**METHODS**			
Protocol and registration	5	Indicate whether a review protocol exists; state if and where it can be accessed (e.g., a Web address); and if available, provide registration information, including the registration number.	6
Eligibility criteria	6	Specify characteristics of the sources of evidence used as eligibility criteria (e.g., years considered, language, and publication status), and provide a rationale.	6
Information sources*	7	Describe all information sources in the search (e.g., databases with dates of coverage and contact with authors to identify additional sources), as well as the date the most recent search was executed.	6
Search	8	Present the full electronic search strategy for at least 1 database, including any limits used, such that it could be repeated.	NA
Selection of sources of evidence†	9	State the process for selecting sources of evidence (i.e., screening and eligibility) included in the scoping review.	6-7
Data charting process‡	10	Describe the methods of charting data from the included sources of evidence (e.g., calibrated forms or forms that have been tested by the team before their use, and whether data charting was done independently or in duplicate) and any processes for obtaining and confirming data from investigators.	7-8
Data items	11	List and define all variables for which data were sought and any assumptions and simplifications made.	7-8
Critical appraisal of individual sources of evidence§	12	If done, provide a rationale for conducting a critical appraisal of included sources of evidence; describe the methods used and how this information was used in any data synthesis (if appropriate).	8
Synthesis of results	13	Describe the methods of handling and summarizing the data that were charted.	8

For peer review only - http://bmjopen.bmj.com/site/about/guidelines.xhtml

SECTION	ITEM	PRISMA-ScR CHECKLIST ITEM	REPORTED ON PAGE #
**RESULTS**			
Selection of sources of evidence	14	Give numbers of sources of evidence screened, assessed for eligibility, and included in the review, with reasons for exclusions at each stage, ideally using a flow diagram.	TBC
Characteristics of sources of evidence	15	For each source of evidence, present characteristics for which data were charted and provide the citations.	TBC
Critical appraisal within sources of evidence	16	If done, present data on critical appraisal of included sources of evidence (see item 12).	TBC
Results of individual sources of evidence	17	For each included source of evidence, present the relevant data that were charted that relate to the review questions and objectives.	TBC
Synthesis of results	18	Summarize and/or present the charting results as they relate to the review questions and objectives.	TBC
**DISCUSSION**			
Summary of evidence	19	Summarize the main results (including an overview of concepts, themes, and types of evidence available), link to the review questions and objectives, and consider the relevance to key groups.	TBC
Limitations	20	Discuss the limitations of the scoping review process.	TBC
Conclusions	21	Provide a general interpretation of the results with respect to the review questions and objectives, as well as potential implications and/or next steps.	TBC
**FUNDING**			
Funding	22	Describe sources of funding for the included sources of evidence, as well as sources of funding for the scoping review. Describe the role of the funders of the scoping review.	2 (research members only)

JBI = Joanna Briggs Institute; PRISMA-ScR = Preferred Reporting Items for Systematic reviews and Meta-Analyses extension for Scoping Reviews.
* Where *sources of evidence* (see second footnote) are compiled from, such as bibliographic databases, social media platforms, and Web sites.
† A more inclusive/heterogeneous term used to account for the different types of evidence or data sources (e.g., quantitative and/or qualitative research, expert opinion, and policy documents) that may be eligible in a scoping review as opposed to only studies. This is not to be confused with *information sources* (see first footnote).
‡ The frameworks by Arksey and O'Malley (6) and Levac and colleagues (7) and the JBI guidance (4, 5) refer to the process of data extraction in a scoping review as data charting.
§ The process of systematically examining research evidence to assess its validity, results, and relevance before using it to inform a decision. This term is used for items 12 and 19 instead of "risk of bias" (which is more applicable to systematic reviews of interventions) to include and acknowledge the various sources of evidence that may be used in a scoping review (e.g., quantitative and/or qualitative research, expert opinion, and policy document).

From: Tricco AC, Lillie E, Zarin W, O'Brien KK, Colquhoun H, Levac D, et al. PRISMA Extension for Scoping Reviews (PRISMAScR): Checklist and Explanation. Ann Intern Med. 2018;169:467–473. doi: 10.7326/M18-0850.

For peer review only - http://bmjopen.bmj.com/site/about/guidelines.xhtml

EVOLution of the data and methods in real world COVID-19 Vaccine Effectiveness studies on mortality: A Scoping Review Protocol

Journal:	*BMJ Open*
Manuscript ID	bmjopen-2023-079071.R2
Article Type:	Protocol
Date Submitted by the Author:	07-Dec-2023

Complete List of Authors:	Stehlik, Paulina; Griffith University Faculty of Health, School of Pharmacy and Medical Sciences; Bond University Faculty of Health Sciences and Medicine, Institute for Evidence-Based Healthcare<br>Dowsett, Caroline; Griffith University Faculty of Health, School of Pharmacy and Medical Sciences<br>Camacho, Ximena; University of New South Wales Medicine & Health, School of Population Health<br>Falster, Michael; University of New South Wales Medicine & Health, School of Population Health<br>Lim, Renly; University of South Australia Division of Health Sciences, Quality Use of Medicines and Pharmacy Research Centre<br>Nasreen, Sharifa; University of Toronto,<br>Pratt, Nicole; University of South Australia Clinical & Health Sciences Academic Unit, Quality Use of Medicines and Pharmacy Research Centre<br>Pearson, Sallie-Anne; University of New South Wales, School of Population Health<br>Henry, David; Bond University Faculty of Health Sciences and Medicine, Institute for Evidence-Based Healthcare; University of New South Wales, School of Population Health

**Primary Subject Heading**:	Infectious diseases
Secondary Subject Heading:	Epidemiology, Research methods, Public health
Keywords:	COVID-19, STATISTICS & RESEARCH METHODS, Protocols & guidelines < HEALTH SERVICES ADMINISTRATION & MANAGEMENT, INFECTIOUS DISEASES, Mortality, PUBLIC HEALTH

SCHOLARONE™
Manuscripts

EVOLution of the data and methods in real world COVID-19 Vaccine Effectiveness studies on mortality: A Scoping Review Protocol

Short title: EVOLVE ScR

FORWARD

AUTHORS

Paulina Stehlik, School of Pharmacy and Medical Sciences, Griffith University; Adjunct Faculty of Health Sciences and Medicine, Bond University*

Caroline Dowsett, School of Pharmacy and Medical Sciences, Griffith University.

Ximena Camacho, School of Population Health, Faculty of Medicine and Health, University of New South Wales

Michael Falster, School of Population Health, Faculty of Medicine and Health, University of New South Wales

Renly Lim, Quality Use of Medicines and Pharmacy Research Centre, UniSA Clinical and Health Sciences, University of South Australia

Sharifa Nasreen, Epidemiology and Biostatistics, School of Public Health, SUNY Downstate Health Sciences University

Nicole Pratt, Quality Use of Medicines and Pharmacy Research Centre, UniSA Clinical and Health Sciences, University of South Australia

Sallie-Anne Pearson, School of Population Health, Faculty of Medicine and Health, University of New South Wales

David Henry, Honorary/ Adjunct, Bond University and University of New South Wales

CORRESPONDING AUTHOR

For peer review only

Dr Paulina Stehlik, School of Pharmacy and Medical Sciences, Griffith University; Adjunct Faculty of Health Sciences and Medicine, Bond University

Email: p.stehlik@griffith.edu.au

ADVISORY GROUP

Bette Liu, UNSW National Centre for Immunisation Research and Surveillance

Andrew Wilson, PBAC advisory committee chair; Director of the Menzies Centre for Health Policy, Menzies Centre for Health Policy, School of Public Health

Kylie Alcorn, A/Director Infectious Diseases, Infectious Disease Department, Gold Coast Health

Alexandra Bannach-Brown, Charité - Universitätsmedizin Berlin

ABSTRACT

Background: Early evidence on COVID-19 vaccine efficacy came from randomised trials. However, many important questions about vaccine effectiveness (VE) in vulnerable groups and against evolving viral variants have been addressed using real-world data. The results of these studies have informed most vaccination policies globally. As the questions about VE have evolved during the pandemic so have data, study design and analytical choices. This scoping review aims to characterise this evolution and provide insights for future pandemic planning – specifically, what kinds of questions are asked at different stages of a pandemic, and what data infrastructure and methods are used to answer these questions?

Methods and analysis: We will identify relevant studies in the Johns Hopkins Bloomberg School of Public Health VIEW-hub database. We will include real-world studies of COVID-19 VE that reported COVID-19-specific or all-cause mortality. We will extract information on study characteristics; study context; data sources; design and analytic methods that address confounding.

A single reviewer will extract data after achieving 80% agreement on a validation set. Variables such as data sources will be categorised using an inductive approach and each study will be discussed in a small group setting. A timeline mapping approach will be used to capture the evolution of this body of literature. Within-country activities will be documented to discern the development of data design and analytic strategies.

Dissemination: This review will provide important information on how study questions, data availability and resulting design choices of VE studies evolved through the COVID-19 pandemic. This review will help identify options for planning and VE studies and inform policy makers on the minimal data and analytic infrastructure needed to support rapid real-

For peer review only

world evaluation of VE in future pandemics. The findings will also be relevant to initiatives to rapidly evaluate effectiveness of health care strategies more broadly.

Registration: https://doi.org/10.17605/OSF.IO/ZHDKR

STRENGTHS AND LIMITATIONS

– We will use a comprehensive curated database (Johns Hopkins Bloomberg School of Public Health VIEW-hub) to identify studies for inclusion in a scoping review of the design methods and data choices used to conduct real world studies of the effectiveness of COVID-19 vaccines in preventing mortality.
– VIEW-hub is a comprehensive database compiled from weekly searches of the literature across multiple databases, preprint servers and the grey literature and defines relevant studies based on a set of quality criteria. It contains details of more than 500 vaccine effectiveness studies.
– Data will be categorised to facilitate data synthesis and mapping. To allow for data exploration we will produce interactive visuals that enable researchers and policy makers to explore the data independently, and we will make our data open access at the conclusion of our study.

For peer review only

INTRODUCTION

The COVID-19 pandemic has been unprecedented in terms of its direct health impacts and disruption of many aspects of modern society. It has also been remarkable in the speed with which scientists and industry collaborated in the production and testing of a range of vaccines.

It became apparent quickly that the COVID-19 vaccines did not stimulate sterilising immunity but provided protection against severe illness and death, most importantly in those with underlying risk factors.(1, 2) The randomised trials that formed the evidence base for the initial deployment of vaccines included few subjects who were elderly, very young, pregnant, had immunodeficiency or severe co-morbidity states.(3) Although quite large, the randomised trials documented few deaths and could not provide precise estimates of the effectiveness of the vaccines in reducing COVID-related and all-cause mortality.

The subsequent evaluation of vaccine effectiveness (VE) using controlled observational studies has been complicated by changes in the infectiousness and virulence of the SARS-CoV-2 virus, and rising background levels of vaccine-induced or naturally acquired immunity. Case fatality rates have fallen substantially, particularly in highly vaccinated countries.(4) Deaths are now concentrated in a group of older patients, those with obesity and those who have serious comorbidities or are immunocompromised.(5) This rapidly changing landscape created a need for continuous 'real-world' studies (RWS) of vaccine effectiveness in susceptible groups, against emerging viral variants and after repeated vaccine doses.(6) These studies use data collected outside of a clinical trials setting to define exposures, endpoints and relevant covariates. This is achieved by analysing data from electronic medical records, administrative records, death registries and registries established specifically to record infection status and vaccine receipt.(6)

Most VE studies of COVID-19 vaccines have employed large population-scale linked routinely collected datasets. However, countries have varied in the timeliness of their response to this major challenge. In some countries, for instance Israel and UK, collaborations between researchers, health service providers and government agencies enabled rapid analyses of large datasets using sophisticated techniques to adjust for confounding and other sources of bias. In contrast, other countries, for instance Australia and Aotearoa/New Zealand, were slow to conduct effectiveness studies, in part because of low infection rates early in the pandemic, and in Australia because of difficulties in accessing the necessary linked datasets.(7, 8)

Systematic reviews of VE studies have concentrated, appropriately, on the vaccines' ability to prevent serious illness and death.(9-12) They have been consistent in confirming that multiple doses of the available vaccines have been associated with large reductions in

For peer review only

mortality, with quite rapid waning (over months) in protection, mandating a need for repeated booster doses.

The COVID-19 pandemic has been a historic event that we must learn from. The rapid deployment of vaccines, followed by studies of their effectiveness, represents the largest and most important healthcare intervention in recent history and one that was evaluated largely using non-randomised studies. The sense of pandemic urgency led to rapid development of strategies to establish datasets, designs, and analytic approaches. This evolution of study questions, data designs and methods through the course of the pandemic provides a unique learning opportunity for policy makers and researchers alike.

We plan to conduct a scoping review of the evidence base on real world Covid-19 vaccine effectiveness to document this evolution; specifically, how policy-relevant questions changed over the course of the pandemic, how these affected the choices of data sources, designs, and analytical methods. By analysing these we hope to provide information that is useful to the following stakeholders:

1. Policy makers and health system managers: by indicating what datasets will have to be created de novo and the need for linkage to existing routinely collected data in responding to future pandemics.
2. Clinicians and laboratory scientists: by identifying the disease manifestations and clinical and demographic vulnerability factors that will inform the designs and analyses of linked datasets needed to evaluate the effectiveness of vaccines and other interventions and how these may change over the course of a future pandemic and can advocate for the appropriate datasets to be linked and made available to researchers.
3. Data scientists and methodologists: to provide guidance as to study designs, analytical and adjustment techniques that are most often used in providing rapid estimates of VE early in a future pandemic; to advocate for the data elements required to deal with confounding to be collected and available in a linked analysable form.
4. Vaccine manufacturers: to understand better the post licensing requirements for vaccines and pharmaceutical products under pandemic conditions and contribute appropriately to the necessary evaluations.
5. The pharmacoepidemiology community generally: the rapid evaluation of vaccine effectiveness during the COVID-19 pandemic provides lessons for the timely investigation of a range of pharmaceutical treatments for emerging health threats.

METHODS

We will conduct a scoping review, following the methods published by the Joanna Briggs Institute(13) and report the results according to the Preferred Reporting Items for Systematic Reviews and Meta-Analyses statement for scoping review (PRISMA-ScR).(14) This

For peer review only

scoping review is registered with the Open Science Framework (OSF; https://doi.org/10.17605/OSF.IO/ZHDKR). Data extraction has begun (25th September 2023, after protocol registration, and will continue for approximately 6 months).

INFORMATION SOURCES AND DATA SELECTION

We will retrieve relevant studies from the VIEW-hub database, maintained by Johns Hopkins Bloomberg School of Public Health. This database includes a wide range of study types including vaccine efficacy trials, vaccine effectiveness studies, impact studies and safety studies. At the time of writing there are more than 500 vaccine effectiveness studies in the database, of which more than 90 reported on Covid-19 related or all-cause mortality, or both. These will be the focus of this review. The VIEW-hub search strategy and inclusion criteria have been described in detail elsewhere.(15) The database includes both published and pre-print studies of vaccine effectiveness identified from PubMed, Embase, Scopus, Web of Science, the WHO COVID Database, MMWR, Eurosurveillance, medRxiv, bioRxiv, SSRN, Europe PMC, Research Square, and Knowledge Hub, as well as Google alerts for COVID-19 vaccine effectiveness studies. A detailed description of the search strategy and inclusion criteria are provided in the VEIW-hub methods paper.(15) Studies are screened weekly by the same two epidemiologists, who also extract some data about included studies. These include study author, title, date published, link to paper, country of origin, vaccine studied, variant studied, population, study start and end date, and outcomes of interest.

To be eligible for inclusion in the VIEW-hub database vaccine effectiveness studies must meet minimum criteria that are appropriate for making causal inference. The studies must include both vaccinated and unvaccinated (or other control) subjects, drawn from a comparable time period, capturing the relevant endpoints in both groups, having a secure record of vaccination (not relying on recall) and free of obvious major methodological flaws. The latter judgment was not based on a strict risk of bias assessment. We are not applying any additional eligibility criteria in our study. Most of the candidate studies were performed with large routinely collected datasets, in some cases augmented by data sources established during the pandemic (e.g., laboratory PCR results).

Studies can be published or pre-prints. Our principal aim is to describe the evolution of observational vaccine effectiveness studies using real world data as these are most relevant to the evaluation of vaccine effectiveness during a constantly changing pandemic. Accordingly, we will not include randomised trials.

The impacts of vaccines on infection and transmission have been limited and transient,(16) and that diminishes the value of infection as the principal study endpoint. The decline in PCR testing and registration of antigen test results have reduced the value of test results as the basis for test negative designs.(17, 18) The nature of COVID-19 related hospitalisations

For peer review only

has changed during the pandemic with an increase in incidental findings of infection through routine testing of patients admitted for other reasons.(18) On the other hand, there has been an increasing focus on excess all-cause mortality as a measure of the success of countries in controlling the spread of the virus and mitigating its negative impacts on healthcare systems.(19, 20) We will therefore restrict our scoping review to all studies from the VIEW-hub database that examine mortality (coded as "death" in the database) as an outcome, either all cause or cause specific.

DATA EXTRACTION

We will extract data on:

1. *Study characteristics*: country, study design, publication status, protocol available, funding sources including whether the study was funded by an independent source or manufacturer, study ethics approval (or waiver), consent requirements (or waiver).
2. *Study context*: reported vaccine policies in place, reported dominant viral variant at time of study.
3. *PICO-T*: inclusion and exclusion criteria, exposure (i.e. vaccine(s)) and definition of exposed, control group, outcome definitions, outcomes collection period, time period of follow up and number of events in the dataset.
4. *Data sources and additional variables*: the types of data sources used (e.g., survey, electronic medical records, administrative data), which were linked at an individual level and which were not, baseline confounders collected, and for adjusted outcomes which variables they were adjusted for;
5. *Analytical strategies to minimise bias*: methods for minimising baseline confounding (e.g., propensity score analysis, instrumental variable analysis, covariate adjustment, self-controlled design etc) and further details of how the methods were implemented as appropriate, such as how the propensity score was implemented (matching, stratification or IPTW) which variables were included in the propensity score model. Additionally, sensitivity analysis conducted, subgroups analysed, methods used for dealing with missing data, and methods used for dealing with time varying environmental risk will be extracted.

We anticipate there will be a few data points where it will be difficult to provide an exhaustive list of potential categories for some of the variables of interest *a priori*. We will therefore take an inductive approach to categorising variables such as "data sources", "inclusion criteria", and "adjustment techniques" by entering in free text and then developing categories through group discussion.

The lead author (PS) will develop a purpose-built data-extraction form developed in SharePoint Lists and a blank copy of the form and data dictionary will be provided on our OSF site. PS will also develop a validation set using a random sample of 7 papers and verified by experts in pharmacoepidemiology (DH) and analysis (XC). A single author (CD) will

For peer review only

independently extract data on the validation set until 80% agreement is achieved, at which stage they will continue with data extraction. A second reviewer (PS) will check the accuracy of all data extractions, and a core team (DH, CD, PS, XC) will meet regularly to discuss each study, whether it meets the inclusion criteria, and the main messages that it provides. The broader study team will meet less frequently to address issues arising and ensure data is categorised in a meaningful way that helps to inform decision making.

All data will be made publicly available via our study's OSF page (https://osf.io/m4cbf/).

ASSESSMENT OF RISK OF BIAS

We aim to describe the evolution of the literature and will therefore not conduct a formal assessment of the risk of bias in the included studies. However, all included studies in the VIEW-hub database must meet a minimal set of quality criteria, and while this does not mean that they are free of bias, the process aims to ensure a baseline level of quality.

DATA SYNTHESIS

To describe the evolution of RWS of COVID-19 VE over the course of the pandemic, we will use descriptive statistics to quantify study characteristics – including evolution of study design (e.g. test-negative designs, cohorts, regression discontinuity), research questions asked (e.g.: comparisons done, effectiveness and waning effect, etc), data sources (e.g. regularly collected population data, registry data), analytic approaches (e.g. by design or form of adjustment), populations included, countries studied, how outcomes were defined, and event rates.

We will provide a temporal sequence of these characteristics overall, and where there are sufficient data within countries, and present them visually (e.g. as annotated stacked area graphs) to establish a template that enables anticipation of study questions and therefore plan for data availability in future pandemics.

We plan to develop interactive visuals as outputs so that stakeholders can interrogate the data further. All data manipulation, analysis and visualisation will occur using Python and R and we will share all code via OSF.

REVIEW TEAM AND CONSULTATION

Our review team and reference group consists of content experts in review methodology, vaccine and drug effectiveness studies, biostatistics and data science. Several have been involved directly in the conduct of VE studies during the Covid-19 pandemic and have a good working knowledge of the relevant literature. Most of the team members are actively involved in the NHMRC-funded Medicines Intelligence Centre for Research Excellence that aims to accelerate real-world evidence development to inform medicines policy decision making.(21) Our reference group comprises end users in infectious diseases and pandemic management, vaccine epidemiology, and medicines and vaccine policy.

For peer review only

All authors and advisory group members have provided comment on this protocol, and the appropriateness of the research questions and data elements. The advisory group will be consulted on how best to present the data so that it is usable and helps with decision making in their respective areas.

In addition, we anticipate that the data we collect could be used for future review automation work that could improve the efficiency of research. Our advisory group also includes an expert in review methodology and automation who will provide advice on future-proofing our dataset.

ETHICS AND DISSEMINATION

As this scoping review will only include data in the public domain ethics review is not required.

Findings of this review will be relevant to several stakeholders, including those involved in pandemic response, data infrastructure and health technology evaluation. As such we will disseminate our findings in five ways: 1) working papers for dissemination to policy makers in Australia; 2) open access publication of findings in peer reviewed journals; 3) presentation of findings at local and international infectious disease, vaccine, health systems and health management conferences. 4) online interactive visual to allow interrogation of the data; 5) open access to our data, code, and preprints via OSF.

PATIENT AND PUBLIC INVOLVEMENT

None.

CONTRIBUTION (CREDIT AUTHOR STATEMENT)

Paulina Stehlik and David Henry conceptualised the project, acquired the funding, and are acting as project supervisors. Paulina Stehlik, Caroline Dowsett, Ximena Camacho, Michael Falster, Renly Lim, Sharifa Nasreen, Nicole Pratt, Sallie-Anne Pearson, and David Henry contributed to the methodology. Paulina Stehlik developed the resources and database, and oversees database and project management. Paulina Stehlik, David Henry and Ximena Camacho piloted the database and extraction tool and developed the validation set. Caroline Dowsette is conducting the data extraction while will be checked by Paulina Stehlik. Paulina Stehlik, Ximena Camacho and Michael Falster developed the data synthesis plan. Paulina Stehlik and David Henry wrote the original draft of this manuscript, and Paulina Stehlik, Caroline Dowsett, Ximena Camacho, Michael Falster, Renly Lim, Sharifa Nasreen, Nicole Pratt, Sallie-Anne Pearson, and David Henry all edited and reviewed the draft and final revisions.

AKNOWLEDGEMENTS

For peer review only

We would like to acknowledge the advisory group and Oyungerel Byambasuren to their feedback and comments on draft versions of this document, particularly on the methodology and which variables to collect to provide meaningful information to decision makers.

FUNDING

Medicines Intelligence Centre for Research Excellence (MI-CRE) 2022 Project Incubator Grant; The MI-CRE is supported by the National Health and Medical Research Council's Centres of Research Excellence (CRE) scheme (ID 1196900)

Renly Lim is supported by a National Health and Medical Research Council (NHMRC) Early Career Fellowship (APP1156368).

Michael Falster is supported by a Future Leader Fellowship from the National Heart Foundation of Australia (ID: 105609).

Ximena Camacho is supported by a NHMRC Postgraduate Scholarship (ID: 2005259).

CONFLICTS OF INTEREST

No additional conflicts of interest to declare.

For peer review only

REFERENCES

1. Polack FP, Thomas SJ, Kitchin N, Absalon J, Gurtman A, Lockhart S, et al. Safety and Efficacy of the BNT162b2 mRNA Covid-19 Vaccine. New England Journal of Medicine. 2020;383(27):2603-15.

2. Voysey M, Clemens SAC, Madhi SA, Weckx LY, Folegatti PM, Aley PK, et al. Safety and efficacy of the ChAdOx1 nCoV-19 vaccine (AZD1222) against SARS-CoV-2: an interim analysis of four randomised controlled trials in Brazil, South Africa, and the UK. The Lancet. 2021;397(10269):99-111.

3. Xiao H, Vaidya R, Liu F, Chang X, Xia X, Unger JM. Sex, Racial, and Ethnic Representation in COVID-19 Clinical Trials: A Systematic Review and Meta-analysis. JAMA Internal Medicine. 2023;183(1):50-60.

4. Haider N, Hasan MN, Guitian J, Khan RA, McCoy D, Ntoumi F, et al. The disproportionate case–fatality ratio of COVID-19 between countries with the highest vaccination rates and the rest of the world. IJID Regions. 2023;6:159-66.

5. Booth A, Reed AB, Ponzo S, Yassaee A, Aral M, Plans D, et al. Population risk factors for severe disease and mortality in COVID-19: A global systematic review and meta-analysis. PLOS ONE. 2021;16(3):e0247461.

6. Swift B, Jain L, White C, Chandrasekaran V, Bhandari A, Hughes DA, et al. Innovation at the Intersection of Clinical Trials and Real-World Data Science to Advance Patient Care. Clinical and Translational Science. 2018;11(5):450-60.

7. Henry D, Stehlik P, Camacho X, Pearson SA. Access to routinely collected data for population health research: experiences in Canada and Australia. Australian and New Zealand Journal of Public Health. 2018;42(5):430-3.

8. Henry DA, Jones MA, Stehlik P, Glasziou PP. Effectiveness of COVID-19 vaccines: findings from real world studies. Medical Journal of Australia. 2021;215(4):149-51.e1.

9. Huang Z, Xu S, Liu J, Wu L, Qiu J, Wang N, et al. Effectiveness of inactivated and Ad5-nCoV COVID-19 vaccines against SARS-CoV-2 Omicron BA. 2 variant infection, severe illness, and death. BMC Medicine. 2022;20(1):400.

10. Prasad S, Kalafat E, Blakeway H, Townsend R, O'Brien P, Morris E, et al. Systematic review and meta-analysis of the effectiveness and perinatal outcomes of COVID-19 vaccination in pregnancy. Nature Communications. 2022;13(1):2414.

11. Rahmani K, Shavaleh R, Forouhi M, Disfani HF, Kamandi M, Oskooi RK, et al. The effectiveness of COVID-19 vaccines in reducing the incidence, hospitalization, and mortality from COVID-19: A systematic review and meta-analysis. Frontiers in Public Health. 2022;10.

12. Kow CS, Hasan SS. Real-world effectiveness of BNT162b2 mRNA vaccine: a meta-analysis of large observational studies. Inflammopharmacology. 2021;29(4):1075-90.

13. DJ Peters M, Godfrey C, McInerney P, Munn Z, Tricco AC, Khalil H. Chapter 11: Scoping Reviews. 2020. In: JBI Manual for Evidence Synthesis [Internet]. https://synthesismanual.jbi.global: JBI.

14. Tricco AC, Lillie E, Zarin W, O'Brien KK, Colquhoun H, Levac D, et al. PRISMA Extension for Scoping Reviews (PRISMA-ScR): Checklist and Explanation. Annals of Internal Medicine. 2018;169(7):467-73.

15. International Vaccine Access Center, Johns Hopkins Bloomberg School of Public Health, World Health Organization, Coalition for Epidemic Preparedness Innovation. Results of COVID-19 Vaccine Effectiveness & Impact Studies: An Ongoing Systematic Review. Methods.: International Vaccine Access Center (IVAC), Johns Hopkins Bloomberg School of Public Health. VIEW-hub.,; 2022 September 2022.

16. Tan ST, Kwan AT, Rodríguez-Barraquer I, Singer BJ, Park HJ, Lewnard JA, et al. Infectiousness of SARS-CoV-2 breakthrough infections and reinfections during the Omicron wave. Nature Medicine. 2023;29(2):358-65.

17. Shi X, Li KQ, Mukherjee B. Current Challenges With the Use of Test-Negative Designs for Modeling COVID-19 Vaccination and Outcomes. American Journal of Epidemiology. 2023;192(3):328-33.

18. UK Health Security Agency working with the Medicines & Healthcare products Regulatory Agency. COVID-19 vaccine surveillance report (2 March 2023). In: Care DoHaS, editor. gov.uk2023.

For peer review only

19. Mukherjee B. Being a Public Health Statistician During a Global Pandemic. Statistical Science. 2022;37(2):270-7, 8.

20. The true death toll of COVID-19: estimating global excess mortality: World Health Organization; [cited 2023 March 7]. Available from: https://www.who.int/data/stories/the-true-death-toll-of-covid-19-estimating-global-excess-mortality.

21. Pratt N, Camacho X, Vajdic C, Degenhardt L, Laba T-L, Hillen J, et al. The Medicines Intelligence Centre of Research Excellence: Co-creating real-world evidence to support the evidentiary needs of Australian medicines regulators and payers. International Journal of Population Data Science. 2021;6(3).

For peer review only

Preferred Reporting Items for Systematic reviews and Meta-Analyses extension for Scoping Reviews (PRISMA-ScR) Checklist

SECTION	ITEM	PRISMA-ScR CHECKLIST ITEM	REPORTED ON PAGE #
**TITLE**			
Title	1	Identify the report as a scoping review.	1
**ABSTRACT**			
Structured summary	2	Provide a structured summary that includes (as applicable): background, objectives, eligibility criteria, sources of evidence, charting methods, results, and conclusions that relate to the review questions and objectives.	3
**INTRODUCTION**			
Rationale	3	Describe the rationale for the review in the context of what is already known. Explain why the review questions/objectives lend themselves to a scoping review approach.	5
Objectives	4	Provide an explicit statement of the questions and objectives being addressed with reference to their key elements (e.g., population or participants, concepts, and context) or other relevant key elements used to conceptualize the review questions and/or objectives.	5
**METHODS**			
Protocol and registration	5	Indicate whether a review protocol exists; state if and where it can be accessed (e.g., a Web address); and if available, provide registration information, including the registration number.	6
Eligibility criteria	6	Specify characteristics of the sources of evidence used as eligibility criteria (e.g., years considered, language, and publication status), and provide a rationale.	6
Information sources*	7	Describe all information sources in the search (e.g., databases with dates of coverage and contact with authors to identify additional sources), as well as the date the most recent search was executed.	6
Search	8	Present the full electronic search strategy for at least 1 database, including any limits used, such that it could be repeated.	NA
Selection of sources of evidence†	9	State the process for selecting sources of evidence (i.e., screening and eligibility) included in the scoping review.	6-7
Data charting process‡	10	Describe the methods of charting data from the included sources of evidence (e.g., calibrated forms or forms that have been tested by the team before their use, and whether data charting was done independently or in duplicate) and any processes for obtaining and confirming data from investigators.	7-8
Data items	11	List and define all variables for which data were sought and any assumptions and simplifications made.	7-8
Critical appraisal of individual sources of evidence§	12	If done, provide a rationale for conducting a critical appraisal of included sources of evidence; describe the methods used and how this information was used in any data synthesis (if appropriate).	8
Synthesis of results	13	Describe the methods of handling and summarizing the data that were charted.	8

St. Michael's
Inspired Care.
Inspiring Science.

For peer review only - http://bmjopen.bmj.com/site/about/guidelines.xhtml

SECTION	ITEM	PRISMA-ScR CHECKLIST ITEM	REPORTED ON PAGE #
**RESULTS**			
Selection of sources of evidence	14	Give numbers of sources of evidence screened, assessed for eligibility, and included in the review, with reasons for exclusions at each stage, ideally using a flow diagram.	TBC
Characteristics of sources of evidence	15	For each source of evidence, present characteristics for which data were charted and provide the citations.	TBC
Critical appraisal within sources of evidence	16	If done, present data on critical appraisal of included sources of evidence (see item 12).	TBC
Results of individual sources of evidence	17	For each included source of evidence, present the relevant data that were charted that relate to the review questions and objectives.	TBC
Synthesis of results	18	Summarize and/or present the charting results as they relate to the review questions and objectives.	TBC
**DISCUSSION**			
Summary of evidence	19	Summarize the main results (including an overview of concepts, themes, and types of evidence available), link to the review questions and objectives, and consider the relevance to key groups.	TBC
Limitations	20	Discuss the limitations of the scoping review process.	TBC
Conclusions	21	Provide a general interpretation of the results with respect to the review questions and objectives, as well as potential implications and/or next steps.	TBC
**FUNDING**			
Funding	22	Describe sources of funding for the included sources of evidence, as well as sources of funding for the scoping review. Describe the role of the funders of the scoping review.	2 (research members only)

JBI = Joanna Briggs Institute; PRISMA-ScR = Preferred Reporting Items for Systematic reviews and Meta-Analyses extension for Scoping Reviews.
* Where *sources of evidence* (see second footnote) are compiled from, such as bibliographic databases, social media platforms, and Web sites.
† A more inclusive/heterogeneous term used to account for the different types of evidence or data sources (e.g., quantitative and/or qualitative research, expert opinion, and policy documents) that may be eligible in a scoping review as opposed to only studies. This is not to be confused with *information sources* (see first footnote).
‡ The frameworks by Arksey and O'Malley (6) and Levac and colleagues (7) and the JBI guidance (4, 5) refer to the process of data extraction in a scoping review as data charting.
§ The process of systematically examining research evidence to assess its validity, results, and relevance before using it to inform a decision. This term is used for items 12 and 19 instead of "risk of bias" (which is more applicable to systematic reviews of interventions) to include and acknowledge the various sources of evidence that may be used in a scoping review (e.g., quantitative and/or qualitative research, expert opinion, and policy document).

From: Tricco AC, Lillie E, Zarin W, O'Brien KK, Colquhoun H, Levac D, et al. PRISMA Extension for Scoping Reviews (PRISMAScR): Checklist and Explanation. Ann Intern Med. 2018;169:467–473. doi: 10.7326/M18-0850.

For peer review only - http://bmjopen.bmj.com/site/about/guidelines.xhtml

BMJ Open

EVOLution of the data and methods in real world COVID-19 Vaccine Effectiveness studies on mortality: A Scoping Review Protocol

Journal:	*BMJ Open*
Manuscript ID	bmjopen-2023-079071.R3
Article Type:	Protocol
Date Submitted by the Author:	16-Jan-2024
Complete List of Authors:	Stehlik, Paulina; Griffith University Faculty of Health, School of Pharmacy and Medical Sciences; Bond University Faculty of Health Sciences and Medicine, Institute for Evidence-Based Healthcare<br>Dowsett, Caroline; Griffith University Faculty of Health, School of Pharmacy and Medical Sciences<br>Camacho, Ximena; University of New South Wales Medicine & Health, School of Population Health<br>Falster, Michael; University of New South Wales Medicine & Health, School of Population Health<br>Lim, Renly; University of South Australia Division of Health Sciences, Quality Use of Medicines and Pharmacy Research Centre<br>Nasreen, Sharifa; SUNY Downstate Health Sciences University School of Public Health<br>Pratt, Nicole; University of South Australia Clinical & Health Sciences Academic Unit, Quality Use of Medicines and Pharmacy Research Centre<br>Pearson, Sallie-Anne; University of New South Wales, School of Population Health<br>Henry, David; Bond University Faculty of Health Sciences and Medicine, Institute for Evidence-Based Healthcare; University of New South Wales, School of Population Health
**Primary Subject Heading**:	Infectious diseases
Secondary Subject Heading:	Epidemiology, Research methods, Public health
Keywords:	COVID-19, STATISTICS & RESEARCH METHODS, Protocols & guidelines < HEALTH SERVICES ADMINISTRATION & MANAGEMENT, INFECTIOUS DISEASES, Mortality, PUBLIC HEALTH

SCHOLARONE™ Manuscripts

EVOLution of the data and methods in real world COVID-19 Vaccine Effectiveness studies on mortality: A Scoping Review Protocol

Short title: EVOLVE ScR

FORWARD

AUTHORS

Paulina Stehlik, School of Pharmacy and Medical Sciences, Griffith University; Adjunct Faculty of Health Sciences and Medicine, Bond University*

Caroline Dowsett, School of Pharmacy and Medical Sciences, Griffith University.

Ximena Camacho, School of Population Health, Faculty of Medicine and Health, University of New South Wales

Michael Falster, School of Population Health, Faculty of Medicine and Health, University of New South Wales

Renly Lim, Quality Use of Medicines and Pharmacy Research Centre, UniSA Clinical and Health Sciences, University of South Australia

Sharifa Nasreen, Epidemiology and Biostatistics, School of Public Health, SUNY Downstate Health Sciences University

Nicole Pratt, Quality Use of Medicines and Pharmacy Research Centre, UniSA Clinical and Health Sciences, University of South Australia

Sallie-Anne Pearson, School of Population Health, Faculty of Medicine and Health, University of New South Wales

David Henry, Honorary/ Adjunct, Bond University and University of New South Wales

CORRESPONDING AUTHOR

For peer review only

Dr Paulina Stehlik, School of Pharmacy and Medical Sciences, Griffith University; Adjunct Faculty of Health Sciences and Medicine, Bond University

Email: p.stehlik@griffith.edu.au

ADVISORY GROUP

Bette Liu, UNSW National Centre for Immunisation Research and Surveillance

Andrew Wilson, PBAC advisory committee chair; Director of the Menzies Centre for Health Policy, Menzies Centre for Health Policy, School of Public Health

Kylie Alcorn, A/Director Infectious Diseases, Infectious Disease Department, Gold Coast Health

Alexandra Bannach-Brown, Charité - Universitätsmedizin Berlin

ABSTRACT

Background: Early evidence on COVID-19 vaccine efficacy came from randomised trials. However, many important questions about vaccine effectiveness (VE) in vulnerable groups and against evolving viral variants have been addressed using real-world data. The results of these studies have informed most vaccination policies globally. As the questions about VE have evolved during the pandemic so have data, study design and analytical choices. This scoping review aims to characterise this evolution and provide insights for future pandemic planning – specifically, what kinds of questions are asked at different stages of a pandemic, and what data infrastructure and methods are used to answer these questions?

Methods and analysis: We will identify relevant studies in the Johns Hopkins Bloomberg School of Public Health VIEW-hub database. We will include real-world studies of COVID-19 VE that reported COVID-19-specific or all-cause mortality. We will extract information on study characteristics; study context; data sources; design and analytic methods that address confounding.

A single reviewer will extract data after achieving 80% agreement on a validation set. Variables such as data sources will be categorised using an inductive approach and each study will be discussed in a small group setting. A timeline mapping approach will be used to capture the evolution of this body of literature. Within-country activities will be documented to discern the development of data design and analytic strategies.

Dissemination: This review will provide important information on how study questions, data availability and resulting design choices of VE studies evolved through the COVID-19 pandemic. This review will help identify options for planning and VE studies and inform policy makers on the minimal data and analytic infrastructure needed to support rapid real-

For peer review only - http://bmjopen.bmj.com/site/about/guidelines.xhtml

world evaluation of VE in future pandemics. The findings will also be relevant to initiatives to rapidly evaluate effectiveness of health care strategies more broadly.

Registration: https://doi.org/10.17605/OSF.IO/ZHDKR

STRENGTHS AND LIMITATIONS

- We will use a comprehensive curated database (Johns Hopkins Bloomberg School of Public Health VIEW-hub) to identify studies for inclusion in a scoping review of the design methods and data choices used to conduct real world studies of the effectiveness of COVID-19 vaccines in preventing mortality.
- VIEW-hub is a comprehensive database compiled from weekly searches of the literature across multiple databases, preprint servers and the grey literature and defines relevant studies based on a set of quality criteria. It contains details of more than 500 vaccine effectiveness studies.
- While use of a curated database may lead to some studies being missed, this is unlikely to change the overall findings of this scoping review.
- Data will be categorised to facilitate data synthesis and mapping. To allow for data exploration we will produce interactive visuals that enable researchers and policy makers to explore the data independently, and we will make our data open access at the conclusion of our study.

For peer review only

INTRODUCTION

The COVID-19 pandemic has been unprecedented in terms of its direct health impacts and disruption of many aspects of modern society. It has also been remarkable in the speed with which scientists and industry collaborated in the production and testing of a range of vaccines.

It became apparent quickly that the COVID-19 vaccines did not stimulate sterilising immunity but provided protection against severe illness and death, most importantly in those with underlying risk factors.(1, 2) The randomised trials that formed the evidence base for the initial deployment of vaccines included few subjects who were elderly, very young, pregnant, had immunodeficiency or severe co-morbidity states.(3) Although quite large, the randomised trials documented few deaths and could not provide precise estimates of the effectiveness of the vaccines in reducing COVID-related and all-cause mortality.

The subsequent evaluation of vaccine effectiveness (VE) using controlled observational studies has been complicated by changes in the infectiousness and virulence of the SARS-CoV-2 virus, and rising background levels of vaccine-induced or naturally acquired immunity. Case fatality rates have fallen substantially, particularly in highly vaccinated countries.(4) Deaths are now concentrated in a group of older patients, those with obesity and those who have serious comorbidities or are immunocompromised.(5) This rapidly changing landscape created a need for continuous 'real-world' studies (RWS) of vaccine effectiveness in susceptible groups, against emerging viral variants and after repeated vaccine doses.(6) These studies use data collected outside of a clinical trials setting to define exposures, endpoints and relevant covariates. This is achieved by analysing data from electronic medical records, administrative records, death registries and registries established specifically to record infection status and vaccine receipt.(6)

Most VE studies of COVID-19 vaccines have employed large population-scale linked routinely collected datasets. However, countries have varied in the timeliness of their response to this major challenge. In some countries, for instance Israel and UK, collaborations between researchers, health service providers and government agencies enabled rapid analyses of large datasets using sophisticated techniques to adjust for confounding and other sources of bias. In contrast, other countries, for instance Australia and Aotearoa/New Zealand, were slow to conduct effectiveness studies, in part because of low infection rates early in the pandemic, and in Australia because of difficulties in accessing the necessary linked datasets.(7, 8)

Systematic reviews of VE studies have concentrated, appropriately, on the vaccines' ability to prevent serious illness and death.(9-12) They have been consistent in confirming that multiple doses of the available vaccines have been associated with large reductions in

For peer review only

mortality, with quite rapid waning (over months) in protection, mandating a need for repeated booster doses.

The COVID-19 pandemic has been a historic event that we must learn from. The rapid deployment of vaccines, followed by studies of their effectiveness, represents the largest and most important healthcare intervention in recent history and one that was evaluated largely using non-randomised studies. The sense of pandemic urgency led to rapid development of strategies to establish datasets, designs, and analytic approaches. This evolution of study questions, data designs and methods through the course of the pandemic provides a unique learning opportunity for policy makers and researchers alike.

We plan to conduct a scoping review of the evidence base on real world Covid-19 vaccine effectiveness to document this evolution; specifically, how policy-relevant questions changed over the course of the pandemic, how these affected the choices of data sources, designs, and analytical methods. By analysing these we hope to provide information that is useful to the following stakeholders:

1. Policy makers and health system managers: by indicating what datasets will have to be created de novo and the need for linkage to existing routinely collected data in responding to future pandemics.
2. Clinicians and laboratory scientists: by identifying the disease manifestations and clinical and demographic vulnerability factors that will inform the designs and analyses of linked datasets needed to evaluate the effectiveness of vaccines and other interventions and how these may change over the course of a future pandemic and can advocate for the appropriate datasets to be linked and made available to researchers.
3. Data scientists and methodologists: to provide guidance as to study designs, analytical and adjustment techniques that are most often used in providing rapid estimates of VE early in a future pandemic; to advocate for the data elements required to deal with confounding to be collected and available in a linked analysable form.
4. Vaccine manufacturers: to understand better the post licensing requirements for vaccines and pharmaceutical products under pandemic conditions and contribute appropriately to the necessary evaluations.
5. The pharmacoepidemiology community generally: the rapid evaluation of vaccine effectiveness during the COVID-19 pandemic provides lessons for the timely investigation of a range of pharmaceutical treatments for emerging health threats.

METHODS

We will conduct a scoping review, following the methods published by the Joanna Briggs Institute(13) and report the results according to the Preferred Reporting Items for Systematic Reviews and Meta-Analyses statement for scoping review (PRISMA-ScR).(14) This

For peer review only

scoping review is registered with the Open Science Framework (OSF; https://doi.org/10.17605/OSF.IO/ZHDKR). Data extraction has begun (25th September 2023, after protocol registration, and will continue for approximately 6 months).

INFORMATION SOURCES AND DATA SELECTION

We will retrieve relevant studies from the VIEW-hub database, maintained by Johns Hopkins Bloomberg School of Public Health. This database includes a wide range of study types including vaccine efficacy trials, vaccine effectiveness studies, impact studies and safety studies. At the time of writing there are more than 500 vaccine effectiveness studies in the database, of which more than 90 reported on Covid-19 related or all-cause mortality, or both. These will be the focus of this review. The VIEW-hub search strategy and inclusion criteria have been described in detail elsewhere.(15) The database includes both published and pre-print studies of vaccine effectiveness identified from PubMed, Embase, Scopus, Web of Science, the WHO COVID Database, MMWR, Eurosurveillance, medRxiv, bioRxiv, SSRN, Europe PMC, Research Square, and Knowledge Hub, as well as Google alerts for COVID-19 vaccine effectiveness studies. A detailed description of the search strategy and inclusion criteria are provided in the VEIW-hub methods paper.(15) Studies are screened weekly by the same two epidemiologists, who also extract some data about included studies. These include study author, title, date published, link to paper, country of origin, vaccine studied, variant studied, population, study start and end date, and outcomes of interest.

To be eligible for inclusion in the VIEW-hub database vaccine effectiveness studies must meet minimum criteria that are appropriate for making causal inference. The studies must include both vaccinated and unvaccinated (or other control) subjects, drawn from a comparable time period, capturing the relevant endpoints in both groups, having a secure record of vaccination (not relying on recall) and free of obvious major methodological flaws. The latter judgment was not based on a strict risk of bias assessment. We are not applying any additional eligibility criteria in our study. Most of the candidate studies were performed with large routinely collected datasets, in some cases augmented by data sources established during the pandemic (e.g., laboratory PCR results).

Studies can be published or pre-prints. Our principal aim is to describe the evolution of observational vaccine effectiveness studies using real world data as these are most relevant to the evaluation of vaccine effectiveness during a constantly changing pandemic. Accordingly, we will not include randomised trials.

The impacts of vaccines on infection and transmission have been limited and transient,(16) and that diminishes the value of infection as the principal study endpoint. The decline in PCR testing and registration of antigen test results have reduced the value of test results as the basis for test negative designs.(17, 18) The nature of COVID-19 related hospitalisations

For peer review only

has changed during the pandemic with an increase in incidental findings of infection through routine testing of patients admitted for other reasons.(18) On the other hand, there has been an increasing focus on excess all-cause mortality as a measure of the success of countries in controlling the spread of the virus and mitigating its negative impacts on healthcare systems.(19, 20) We will therefore restrict our scoping review to all studies from the VIEW-hub database that examine mortality (coded as "death" in the database) as an outcome, either all cause or cause specific.

DATA EXTRACTION

We will extract data on:

1. *Study characteristics*: country, study design, publication status, protocol available, funding sources including whether the study was funded by an independent source or manufacturer, study ethics approval (or waiver), consent requirements (or waiver).
2. *Study context*: reported vaccine policies in place, reported dominant viral variant at time of study.
3. *PICO-T*: inclusion and exclusion criteria, exposure (i.e. vaccine(s)) and definition of exposed, control group, outcome definitions, outcomes collection period, time period of follow up and number of events in the dataset.
4. *Data sources and additional variables*: the types of data sources used (e.g., survey, electronic medical records, administrative data), which were linked at an individual level and which were not, baseline confounders collected, and for adjusted outcomes which variables they were adjusted for;
5. *Analytical strategies to minimise bias*: methods for minimising baseline confounding (e.g., propensity score analysis, instrumental variable analysis, covariate adjustment, self-controlled design etc) and further details of how the methods were implemented as appropriate, such as how the propensity score was implemented (matching, stratification or IPTW) which variables were included in the propensity score model. Additionally, sensitivity analysis conducted, subgroups analysed, methods used for dealing with missing data, and methods used for dealing with time varying environmental risk will be extracted.

We anticipate there will be a few data points where it will be difficult to provide an exhaustive list of potential categories for some of the variables of interest *a priori*. We will therefore take an inductive approach to categorising variables such as "data sources", "inclusion criteria", and "adjustment techniques" by entering in free text and then developing categories through group discussion.

The lead author (PS) will develop a purpose-built data-extraction form developed in SharePoint Lists and a blank copy of the form and data dictionary will be provided on our OSF site. PS will also develop a validation set using a random sample of 7 papers and verified by experts in pharmacoepidemiology (DH) and analysis (XC). A single author (CD) will

For peer review only

independently extract data on the validation set until 80% agreement is achieved, at which stage they will continue with data extraction. A second reviewer (PS) will check the accuracy of all data extractions, and a core team (DH, CD, PS, XC) will meet regularly to discuss each study, whether it meets the inclusion criteria, and the main messages that it provides. The broader study team will meet less frequently to address issues arising and ensure data is categorised in a meaningful way that helps to inform decision making.

All data will be made publicly available via our study's OSF page (https://osf.io/m4cbf/).

ASSESSMENT OF RISK OF BIAS

We aim to describe the evolution of the literature and will therefore not conduct a formal assessment of the risk of bias in the included studies. However, all included studies in the VIEW-hub database must meet a minimal set of quality criteria, and while this does not mean that they are free of bias, the process aims to ensure a baseline level of quality.

DATA SYNTHESIS

To describe the evolution of RWS of COVID-19 VE over the course of the pandemic, we will use descriptive statistics to quantify study characteristics – including evolution of study design (e.g. test-negative designs, cohorts, regression discontinuity), research questions asked (e.g.: comparisons done, effectiveness and waning effect, etc), data sources (e.g. regularly collected population data, registry data), analytic approaches (e.g. by design or form of adjustment), populations included, countries studied, how outcomes were defined, and event rates.

We will provide a temporal sequence of these characteristics overall, and where there are sufficient data within countries, and present them visually (e.g. as annotated stacked area graphs) to establish a template that enables anticipation of study questions and therefore plan for data availability in future pandemics.

We plan to develop interactive visuals as outputs so that stakeholders can interrogate the data further. All data manipulation, analysis and visualisation will occur using Python and R and we will share all code via OSF.

REVIEW TEAM AND CONSULTATION

Our review team and reference group consists of content experts in review methodology, vaccine and drug effectiveness studies, biostatistics and data science. Several have been involved directly in the conduct of VE studies during the Covid-19 pandemic and have a good working knowledge of the relevant literature. Most of the team members are actively involved in the NHMRC-funded Medicines Intelligence Centre for Research Excellence that aims to accelerate real-world evidence development to inform medicines policy decision making.(21) Our reference group comprises end users in infectious diseases and pandemic management, vaccine epidemiology, and medicines and vaccine policy.

For peer review only

All authors and advisory group members have provided comment on this protocol, and the appropriateness of the research questions and data elements. The advisory group will be consulted on how best to present the data so that it is usable and helps with decision making in their respective areas.

In addition, we anticipate that the data we collect could be used for future review automation work that could improve the efficiency of research. Our advisory group also includes an expert in review methodology and automation who will provide advice on future-proofing our dataset.

ETHICS AND DISSEMINATION

As this scoping review will only include data in the public domain ethics review is not required.

Findings of this review will be relevant to several stakeholders, including those involved in pandemic response, data infrastructure and health technology evaluation. As such we will disseminate our findings in five ways: 1) working papers for dissemination to policy makers in Australia; 2) open access publication of findings in peer reviewed journals; 3) presentation of findings at local and international infectious disease, vaccine, health systems and health management conferences. 4) online interactive visual to allow interrogation of the data; 5) open access to our data, code, and preprints via OSF.

PATIENT AND PUBLIC INVOLVEMENT

None.

CONTRIBUTION (CREDIT AUTHOR STATEMENT)

Paulina Stehlik and David Henry conceptualised the project, acquired the funding, and are acting as project supervisors. Paulina Stehlik, Caroline Dowsett, Ximena Camacho, Michael Falster, Renly Lim, Sharifa Nasreen, Nicole Pratt, Sallie-Anne Pearson, and David Henry contributed to the methodology. Paulina Stehlik developed the resources and database, and oversees database and project management. Paulina Stehlik, David Henry and Ximena Camacho piloted the database and extraction tool and developed the validation set. Caroline Dowsette is conducting the data extraction while will be checked by Paulina Stehlik. Paulina Stehlik, Ximena Camacho and Michael Falster developed the data synthesis plan. Paulina Stehlik and David Henry wrote the original draft of this manuscript, and Paulina Stehlik, Caroline Dowsett, Ximena Camacho, Michael Falster, Renly Lim, Sharifa Nasreen, Nicole Pratt, Sallie-Anne Pearson, and David Henry all edited and reviewed the draft and final revisions.

AKNOWLEDGEMENTS

For peer review only

We would like to acknowledge the advisory group and Oyungerel Byambasuren to their feedback and comments on draft versions of this document, particularly on the methodology and which variables to collect to provide meaningful information to decision makers.

FUNDING

Medicines Intelligence Centre for Research Excellence (MI-CRE) 2022 Project Incubator Grant; The MI-CRE is supported by the National Health and Medical Research Council's Centres of Research Excellence (CRE) scheme (ID 1196900)

Renly Lim is supported by a National Health and Medical Research Council (NHMRC) Early Career Fellowship (APP1156368).

Michael Falster is supported by a Future Leader Fellowship from the National Heart Foundation of Australia (ID: 105609).

Ximena Camacho is supported by a NHMRC Postgraduate Scholarship (ID: 2005259).

CONFLICTS OF INTEREST

No additional conflicts of interest to declare.

For peer review only

REFERENCES

1. Polack FP, Thomas SJ, Kitchin N, Absalon J, Gurtman A, Lockhart S, et al. Safety and Efficacy of the BNT162b2 mRNA Covid-19 Vaccine. New England Journal of Medicine. 2020;383(27):2603-15.

2. Voysey M, Clemens SAC, Madhi SA, Weckx LY, Folegatti PM, Aley PK, et al. Safety and efficacy of the ChAdOx1 nCoV-19 vaccine (AZD1222) against SARS-CoV-2: an interim analysis of four randomised controlled trials in Brazil, South Africa, and the UK. The Lancet. 2021;397(10269):99-111.

3. Xiao H, Vaidya R, Liu F, Chang X, Xia X, Unger JM. Sex, Racial, and Ethnic Representation in COVID-19 Clinical Trials: A Systematic Review and Meta-analysis. JAMA Internal Medicine. 2023;183(1):50-60.

4. Haider N, Hasan MN, Guitian J, Khan RA, McCoy D, Ntoumi F, et al. The disproportionate case–fatality ratio of COVID-19 between countries with the highest vaccination rates and the rest of the world. IJID Regions. 2023;6:159-66.

5. Booth A, Reed AB, Ponzo S, Yassaee A, Aral M, Plans D, et al. Population risk factors for severe disease and mortality in COVID-19: A global systematic review and meta-analysis. PLOS ONE. 2021;16(3):e0247461.

6. Swift B, Jain L, White C, Chandrasekaran V, Bhandari A, Hughes DA, et al. Innovation at the Intersection of Clinical Trials and Real-World Data Science to Advance Patient Care. Clinical and Translational Science. 2018;11(5):450-60.

7. Henry D, Stehlik P, Camacho X, Pearson SA. Access to routinely collected data for population health research: experiences in Canada and Australia. Australian and New Zealand Journal of Public Health. 2018;42(5):430-3.

8. Henry DA, Jones MA, Stehlik P, Glasziou PP. Effectiveness of COVID-19 vaccines: findings from real world studies. Medical Journal of Australia. 2021;215(4):149-51.e1.

9. Huang Z, Xu S, Liu J, Wu L, Qiu J, Wang N, et al. Effectiveness of inactivated and Ad5-nCoV COVID-19 vaccines against SARS-CoV-2 Omicron BA. 2 variant infection, severe illness, and death. BMC Medicine. 2022;20(1):400.

10. Prasad S, Kalafat E, Blakeway H, Townsend R, O'Brien P, Morris E, et al. Systematic review and meta-analysis of the effectiveness and perinatal outcomes of COVID-19 vaccination in pregnancy. Nature Communications. 2022;13(1):2414.

11. Rahmani K, Shavaleh R, Forouhi M, Disfani HF, Kamandi M, Oskooi RK, et al. The effectiveness of COVID-19 vaccines in reducing the incidence, hospitalization, and mortality from COVID-19: A systematic review and meta-analysis. Frontiers in Public Health. 2022;10.

12. Kow CS, Hasan SS. Real-world effectiveness of BNT162b2 mRNA vaccine: a meta-analysis of large observational studies. Inflammopharmacology. 2021;29(4):1075-90.

13. DJ Peters M, Godfrey C, McInerney P, Munn Z, Tricco AC, Khalil H. Chapter 11: Scoping Reviews. 2020. In: JBI Manual for Evidence Synthesis [Internet]. https://synthesismanual.jbi.global: JBI.

14. Tricco AC, Lillie E, Zarin W, O'Brien KK, Colquhoun H, Levac D, et al. PRISMA Extension for Scoping Reviews (PRISMA-ScR): Checklist and Explanation. Annals of Internal Medicine. 2018;169(7):467-73.

15. International Vaccine Access Center, Johns Hopkins Bloomberg School of Public Health, World Health Organization, Coalition for Epidemic Preparedness Innovation. Results of COVID-19 Vaccine Effectiveness & Impact Studies: An Ongoing Systematic Review. Methods.: International Vaccine Access Center (IVAC), Johns Hopkins Bloomberg School of Public Health. VIEW-hub.,; 2022 September 2022.

16. Tan ST, Kwan AT, Rodríguez-Barraquer I, Singer BJ, Park HJ, Lewnard JA, et al. Infectiousness of SARS-CoV-2 breakthrough infections and reinfections during the Omicron wave. Nature Medicine. 2023;29(2):358-65.

17. Shi X, Li KQ, Mukherjee B. Current Challenges With the Use of Test-Negative Designs for Modeling COVID-19 Vaccination and Outcomes. American Journal of Epidemiology. 2023;192(3):328-33.

18. UK Health Security Agency working with the Medicines & Healthcare products Regulatory Agency. COVID-19 vaccine surveillance report (2 March 2023). In: Care DoHaS, editor. gov.uk2023.

For peer review only

19. Mukherjee B. Being a Public Health Statistician During a Global Pandemic. Statistical Science. 2022;37(2):270-7, 8.

20. The true death toll of COVID-19: estimating global excess mortality: World Health Organization; [cited 2023 March 7]. Available from: https://www.who.int/data/stories/the-true-death-toll-of-covid-19-estimating-global-excess-mortality.

21. Pratt N, Camacho X, Vajdic C, Degenhardt L, Laba T-L, Hillen J, et al. The Medicines Intelligence Centre of Research Excellence: Co-creating real-world evidence to support the evidentiary needs of Australian medicines regulators and payers. International Journal of Population Data Science. 2021;6(3).

For peer review only

Preferred Reporting Items for Systematic reviews and Meta-Analyses extension for Scoping Reviews (PRISMA-ScR) Checklist

SECTION	ITEM	PRISMA-ScR CHECKLIST ITEM	REPORTED ON PAGE #
**TITLE**			
Title	1	Identify the report as a scoping review.	1
**ABSTRACT**			
Structured summary	2	Provide a structured summary that includes (as applicable): background, objectives, eligibility criteria, sources of evidence, charting methods, results, and conclusions that relate to the review questions and objectives.	3
**INTRODUCTION**			
Rationale	3	Describe the rationale for the review in the context of what is already known. Explain why the review questions/objectives lend themselves to a scoping review approach.	5
Objectives	4	Provide an explicit statement of the questions and objectives being addressed with reference to their key elements (e.g., population or participants, concepts, and context) or other relevant key elements used to conceptualize the review questions and/or objectives.	5
**METHODS**			
Protocol and registration	5	Indicate whether a review protocol exists; state if and where it can be accessed (e.g., a Web address); and if available, provide registration information, including the registration number.	6
Eligibility criteria	6	Specify characteristics of the sources of evidence used as eligibility criteria (e.g., years considered, language, and publication status), and provide a rationale.	6
Information sources*	7	Describe all information sources in the search (e.g., databases with dates of coverage and contact with authors to identify additional sources), as well as the date the most recent search was executed.	6
Search	8	Present the full electronic search strategy for at least 1 database, including any limits used, such that it could be repeated.	NA
Selection of sources of evidence†	9	State the process for selecting sources of evidence (i.e., screening and eligibility) included in the scoping review.	6-7
Data charting process‡	10	Describe the methods of charting data from the included sources of evidence (e.g., calibrated forms or forms that have been tested by the team before their use, and whether data charting was done independently or in duplicate) and any processes for obtaining and confirming data from investigators.	7-8
Data items	11	List and define all variables for which data were sought and any assumptions and simplifications made.	7-8
Critical appraisal of individual sources of evidence§	12	If done, provide a rationale for conducting a critical appraisal of included sources of evidence; describe the methods used and how this information was used in any data synthesis (if appropriate).	8
Synthesis of results	13	Describe the methods of handling and summarizing the data that were charted.	8

St. Michael's
Inspired Care.
Inspiring Science.

For peer review only - http://bmjopen.bmj.com/site/about/guidelines.xhtml

SECTION	ITEM	PRISMA-ScR CHECKLIST ITEM	REPORTED ON PAGE #
**RESULTS**			
Selection of sources of evidence	14	Give numbers of sources of evidence screened, assessed for eligibility, and included in the review, with reasons for exclusions at each stage, ideally using a flow diagram.	TBC
Characteristics of sources of evidence	15	For each source of evidence, present characteristics for which data were charted and provide the citations.	TBC
Critical appraisal within sources of evidence	16	If done, present data on critical appraisal of included sources of evidence (see item 12).	TBC
Results of individual sources of evidence	17	For each included source of evidence, present the relevant data that were charted that relate to the review questions and objectives.	TBC
Synthesis of results	18	Summarize and/or present the charting results as they relate to the review questions and objectives.	TBC
**DISCUSSION**			
Summary of evidence	19	Summarize the main results (including an overview of concepts, themes, and types of evidence available), link to the review questions and objectives, and consider the relevance to key groups.	TBC
Limitations	20	Discuss the limitations of the scoping review process.	TBC
Conclusions	21	Provide a general interpretation of the results with respect to the review questions and objectives, as well as potential implications and/or next steps.	TBC
**FUNDING**			
Funding	22	Describe sources of funding for the included sources of evidence, as well as sources of funding for the scoping review. Describe the role of the funders of the scoping review.	2 (research members only)

JBI = Joanna Briggs Institute; PRISMA-ScR = Preferred Reporting Items for Systematic reviews and Meta-Analyses extension for Scoping Reviews.
* Where *sources of evidence* (see second footnote) are compiled from, such as bibliographic databases, social media platforms, and Web sites.
† A more inclusive/heterogeneous term used to account for the different types of evidence or data sources (e.g., quantitative and/or qualitative research, expert opinion, and policy documents) that may be eligible in a scoping review as opposed to only studies. This is not to be confused with *information sources* (see first footnote).
‡ The frameworks by Arksey and O'Malley (6) and Levac and colleagues (7) and the JBI guidance (4, 5) refer to the process of data extraction in a scoping review as data charting.
§ The process of systematically examining research evidence to assess its validity, results, and relevance before using it to inform a decision. This term is used for items 12 and 19 instead of "risk of bias" (which is more applicable to systematic reviews of interventions) to include and acknowledge the various sources of evidence that may be used in a scoping review (e.g., quantitative and/or qualitative research, expert opinion, and policy document).

From: Tricco AC, Lillie E, Zarin W, O'Brien KK, Colquhoun H, Levac D, et al. PRISMA Extension for Scoping Reviews (PRISMAScR): Checklist and Explanation. Ann Intern Med. 2018;169:467–473. doi: 10.7326/M18-0850.

For peer review only - http://bmjopen.bmj.com/site/about/guidelines.xhtml

BMJ Open

EVOLution of the data and methods in real world COVID-19 Vaccine Effectiveness studies on mortality: A Scoping Review Protocol

Journal:	*BMJ Open*
Manuscript ID	bmjopen-2023-079071.R4
Article Type:	Protocol
Date Submitted by the Author:	08-Feb-2024

Complete List of Authors:	Stehlik, Paulina; Griffith University Faculty of Health, School of Pharmacy and Medical Sciences; Bond University Faculty of Health Sciences and Medicine, Institute for Evidence-Based Healthcare<br>Dowsett, Caroline; Griffith University Faculty of Health, School of Pharmacy and Medical Sciences<br>Camacho, Ximena; University of New South Wales Medicine & Health, School of Population Health<br>Falster, Michael; University of New South Wales Medicine & Health, School of Population Health<br>Lim, Renly; University of South Australia Division of Health Sciences, Quality Use of Medicines and Pharmacy Research Centre<br>Nasreen, Sharifa; SUNY Downstate Health Sciences University School of Public Health<br>Pratt, Nicole; University of South Australia Clinical & Health Sciences Academic Unit, Quality Use of Medicines and Pharmacy Research Centre<br>Pearson, Sallie-Anne; University of New South Wales, School of Population Health<br>Henry, David; Bond University Faculty of Health Sciences and Medicine, Institute for Evidence-Based Healthcare; University of New South Wales, School of Population Health

**Primary Subject Heading**:	Infectious diseases
Secondary Subject Heading:	Epidemiology, Research methods, Public health
Keywords:	COVID-19, STATISTICS & RESEARCH METHODS, Protocols & guidelines < HEALTH SERVICES ADMINISTRATION & MANAGEMENT, INFECTIOUS DISEASES, Mortality, PUBLIC HEALTH

SCHOLARONE™
Manuscripts

EVOLution of the data and methods in real world COVID-19 Vaccine Effectiveness studies on mortality: A Scoping Review Protocol

Short title: EVOLVE ScR

FORWARD

AUTHORS

Paulina Stehlik, School of Pharmacy and Medical Sciences, Griffith University; Adjunct Faculty of Health Sciences and Medicine, Bond University*

Caroline Dowsett, School of Pharmacy and Medical Sciences, Griffith University.

Ximena Camacho, School of Population Health, Faculty of Medicine and Health, University of New South Wales

Michael Falster, School of Population Health, Faculty of Medicine and Health, University of New South Wales

Renly Lim, Quality Use of Medicines and Pharmacy Research Centre, UniSA Clinical and Health Sciences, University of South Australia

Sharifa Nasreen, Epidemiology and Biostatistics, School of Public Health, SUNY Downstate Health Sciences University

Nicole Pratt, Quality Use of Medicines and Pharmacy Research Centre, UniSA Clinical and Health Sciences, University of South Australia

Sallie-Anne Pearson, School of Population Health, Faculty of Medicine and Health, University of New South Wales

David Henry, Honorary/ Adjunct, Bond University and University of New South Wales

CORRESPONDING AUTHOR

For peer review only

Dr Paulina Stehlik, School of Pharmacy and Medical Sciences, Griffith University; Adjunct Faculty of Health Sciences and Medicine, Bond University

Email: p.stehlik@griffith.edu.au

ADVISORY GROUP

Bette Liu, UNSW National Centre for Immunisation Research and Surveillance

Andrew Wilson, PBAC advisory committee chair; Director of the Menzies Centre for Health Policy, Menzies Centre for Health Policy, School of Public Health

Kylie Alcorn, A/Director Infectious Diseases, Infectious Disease Department, Gold Coast Health

Alexandra Bannach-Brown, Charité - Universitätsmedizin Berlin

ABSTRACT

Background: Early evidence on COVID-19 vaccine efficacy came from randomised trials. Many important questions subsequently about vaccine effectiveness (VE) have been addressed using real-world data and have informed most vaccination policies globally. As the questions about VE have evolved during the pandemic so have data, study design and analytical choices. This scoping review aims to characterise this evolution and provide insights for future pandemic planning – specifically, what kinds of questions are asked at different stages of a pandemic, and what data infrastructure and methods are used?

Methods and analysis: We will identify relevant studies in the Johns Hopkins Bloomberg School of Public Health VIEW-hub database, which curates both published and pre-print VE studies identified from PubMed, Embase, Scopus, Web of Science, the WHO COVID Database, MMWR, Eurosurveillance, medRxiv, bioRxiv, SSRN, Europe PMC, Research Square, and Knowledge Hub, and Google. We will include real-world studies of COVID-19 VE that reported COVID-19-specific or all-cause mortality (coded as “death” in the “effectiveness studies” dataset).

Information on study characteristics; study context; data sources; design and analytic methods that address confounding will be extracted by single reviewer and checked for accuracy. In addition, each study will be discussed in a small group setting by methodological and analytic experts. A timeline mapping approach will be used to capture the evolution of this body of literature.

Ethics and dissemination: This review will provide important information on how study questions, data availability and resulting design choices of VE studies evolved through the COVID-19 pandemic. It will help identify options for planning VE studies and inform policy makers on the minimal data and analytic infrastructure needed to support rapid real-world

For peer review only

evaluation of VE in future pandemics. The findings will also be relevant to initiatives to rapidly evaluate effectiveness of health care strategies more broadly.

Registration: https://doi.org/10.17605/OSF.IO/ZHDKR

STRENGTHS AND LIMITATIONS

- We will use a comprehensive curated database (Johns Hopkins Bloomberg School of Public Health VIEW-hub that compiles relevant studies on a weekly basis from multiple databases, preprint servers and the grey literature.
- While use of a curated database may lead to some studies being missed, this is unlikely to change the overall findings of this scoping review.
- All extraction will be conducted by a single author to ensure consistency in extraction and checked by a second author to ensure accuracy.
- Weekly group discussions about the individual studies and coding of data will strengthen data integrity.
- End users have been involved in the design of this study and will continue to be consulted throughout its conduct.

For peer review only

INTRODUCTION

The COVID-19 pandemic has been unprecedented in terms of its direct health impacts and disruption of many aspects of modern society. It has also been remarkable in the speed with which scientists and industry collaborated in the production and testing of a range of vaccines.

It became apparent quickly that the COVID-19 vaccines did not stimulate sterilising immunity but provided protection against severe illness and death, most importantly in those with underlying risk factors.(1, 2) The randomised trials that formed the evidence base for the initial deployment of vaccines included few subjects who were elderly, very young, pregnant, had immunodeficiency or severe co-morbidity states.(3) Although quite large, the randomised trials documented few deaths and could not provide precise estimates of the effectiveness of the vaccines in reducing COVID-related and all-cause mortality.

The subsequent evaluation of vaccine effectiveness (VE) using controlled observational studies has been complicated by changes in the infectiousness and virulence of the SARS-CoV-2 virus, and rising background levels of vaccine-induced or naturally acquired immunity. Case fatality rates have fallen substantially, particularly in highly vaccinated countries.(4) Deaths are now concentrated in a group of older patients, those with obesity and those who have serious comorbidities or are immunocompromised.(5) This rapidly changing landscape created a need for continuous ‘real-world’ studies (RWS) of vaccine effectiveness in susceptible groups, against emerging viral variants and after repeated vaccine doses.(6) These studies use data collected outside of clinical trial settings to define exposures, endpoints and relevant covariates. This is achieved by analysing data from electronic medical records, administrative records, death registries and registries established specifically to record infection status and vaccine receipt.(6)

Most VE studies of COVID-19 vaccines have employed large population-scale linked routinely collected datasets. However, countries have varied in the timeliness of their response to this major challenge. In some countries, for instance Israel and UK, collaborations between researchers, health service providers and government agencies enabled rapid analyses of large datasets using sophisticated techniques to adjust for confounding and other sources of bias. In contrast, other countries, for instance Australia and Aotearoa/New Zealand, were slow to conduct effectiveness studies, in part because of low infection rates early in the pandemic, and in Australia because of difficulties in accessing the necessary linked datasets.(7, 8)

Systematic reviews of VE studies have concentrated, appropriately, on the vaccines’ ability to prevent serious illness and death.(9-12) They have been consistent in confirming that multiple doses of the available vaccines have been associated with large reductions in mortality, with quite rapid waning (over months) in protection, mandating a need for

For peer review only

repeated booster doses. As the impacts of vaccines on infection and transmission have been limited and transient,(13) it diminishes the value of infection as the principal study endpoint. The decline in PCR testing and registration of antigen test results have reduced the value of test results as the basis for test negative designs.(14, 15) The nature of COVID-19 related hospitalisations has changed during the pandemic with an increase in incidental findings of infection through routine testing of patients admitted for other reasons.(15) On the other hand, there has been an increasing focus on excess all-cause mortality as a measure of the success of countries in controlling the spread of the virus and mitigating its negative impacts on healthcare systems.(16, 17)

The COVID-19 pandemic has been a historic event that we must learn from. The rapid deployment of vaccines, followed by studies of their effectiveness, represents the largest and most important healthcare intervention in recent history and one that was evaluated largely using non-randomised studies. The sense of pandemic urgency led to rapid development of strategies to establish datasets, designs, and analytic approaches. This evolution of study questions, data designs and methods through the course of the pandemic provides a unique learning opportunity for policy makers and researchers alike.

We plan to conduct a scoping review of the evidence base on real world Covid-19 vaccine effectiveness, focusing on studies that report on death as an outcome, to document this evolution. Specifically we will explore: how policy-relevant questions changed over the course of the pandemic, and how these affected the choices of data sources, designs, and analytical methods. By analysing these we hope to provide information that is useful to the following stakeholders:

1. Policy makers and health system managers: by indicating what datasets will have to be created de novo and the need for linkage to existing routinely collected data in responding to future pandemics.
2. Clinicians and laboratory scientists: by identifying the disease manifestations and clinical and demographic vulnerability factors that will be required to inform the designs and analyses needed to evaluate the effectiveness of vaccines and other interventions, how these may change over the course of a future pandemic, and how the clinical community can advocate for the appropriate data elements to be linked and made available to researchers.
3. Data scientists and methodologists: to provide guidance as to study designs, analytical and adjustment techniques that are most often used in providing rapid estimates of VE early in a future pandemic; to advocate for the data elements required to deal with confounding to be collected and available in a linked analysable form.
4. Vaccine manufacturers: to understand better the post licensing requirements for vaccines and pharmaceutical products under pandemic conditions and contribute appropriately to the necessary evaluations.

For peer review only

5. The pharmacoepidemiology community generally: the rapid evaluation of vaccine effectiveness during the COVID-19 pandemic provides lessons for the timely investigation of a range of pharmaceutical treatments for emerging health threats.

METHODS

We will conduct a scoping review, following the methods published by the Joanna Briggs Institute(18) and report the results according to the Preferred Reporting Items for Systematic Reviews and Meta-Analyses statement for scoping review (PRISMA-ScR).(19) This scoping review is registered with the Open Science Framework (OSF; https://doi.org/10.17605/OSF.IO/ZHDKR). Data extraction has begun (25th September 2023, after protocol registration), and will continue for approximately 6 months.

INFORMATION SOURCES AND DATA SELECTION

We will retrieve relevant studies from the VIEW-hub database,(20) maintained by Johns Hopkins Bloomberg School of Public Health. This database includes a wide range of study types including vaccine efficacy trials, vaccine effectiveness studies, impact studies and safety studies. As our principal aim is to describe the evolution of observational vaccine effectiveness studies using real world data, we used the VIEW-hub "effectiveness studies" dataset.

The VIEW-hub search strategy and inclusion criteria for this dataset have been described in detail elsewhere. (see Supplementary File)(21) Briefly, the "effectiveness studies" dataset includes both published and pre-print studies of vaccine effectiveness identified from PubMed, Embase, Scopus, Web of Science, the WHO COVID Database, MMWR, Eurosurveillance, medRxiv, bioRxiv, SSRN, Europe PMC, Research Square, and Knowledge Hub, as well as Google alerts for COVID-19 vaccine effectiveness studies. Studies are screened weekly by the same two epidemiologists at Johns Hopkins Bloomberg School of Public Health, and the following data elements are extracted for studies included in the dataset: study author, title, date published, link to paper, country of origin, vaccine studied, variant studied, population, study start and end date, and outcomes of interest. Studies in the dataset can be filtered by the vaccine, variant, outcomes, study population and region variables through drop-down menus.

Studies must also meet minimum criteria for causal inference studies using real-world data. The studies must include both vaccinated and unvaccinated (or other control) subjects, drawn from a comparable time period, capturing the relevant endpoints in both groups, having a secure record of vaccination (not relying on recall) and be free of obvious major methodological flaws. The latter judgment was not based on a strict risk of bias assessment.

For peer review only

To identify studies in the VIEW-hub's "effectiveness studies" dataset that examine mortality (either all-cause or cause-specific) we will use the drop-down menu feature to select study outcomes coded as "death". No additional eligibility criteria will be applied.

At the time of writing this protocol (1 Aug 2023) the VIEW-hub database lists 495 observational studies of vaccine effectiveness from 50 countries, and 92 (~19%) list "death" as an endpoint.

DATA EXTRACTION

We will extract data on:

1. *Study characteristics*: country, study design, publication status, protocol available, funding sources (including whether the study was funded by an independent source or manufacturer), study ethics approval (or waiver), consent requirements (or waiver).
2. *Study context*: reported vaccine policies in place, reported dominant viral variant at time of study.
3. *PICO-T*: inclusion and exclusion criteria, exposure (i.e. vaccine(s)) and definition of exposure, control group, outcome definitions, outcomes collection period duration of follow up and number of deaths.
4. *Data sources and additional variables*: the types of data sources used (e.g., survey, electronic medical records, administrative data), which were linked at an individual level and which were not, baseline confounders collected, and for adjusted outcomes which variables they were adjusted for;
5. *Analytical strategies to minimise bias*: methods for minimising baseline confounding (e.g., propensity score analysis, instrumental variable analysis, covariate adjustment, self-controlled designs, etc.) and further details of how the methods were implemented as appropriate, such as how the propensity score was implemented (matching, stratification or inverse probability of treatment weights) and which variables were included in the propensity score model. Additionally, we will extract details on whether a sensitivity analyses was conducted, subgroups analysed, methods used for dealing with missing data, and methods used for dealing with time varying environmental risk.

We anticipate there will be a few data points where it will be difficult to provide an exhaustive list of potential categories for some of the variables of interest *a priori*. We will therefore take an inductive approach to categorising variables such as "data sources", "inclusion criteria", and "adjustment techniques" by entering them in free text and then developing categories through group discussion.

The lead author (PS) will develop a purpose-built data-extraction form in SharePoint Lists and a blank copy of the form and data dictionary will be provided on our OSF site. PS will also develop a validation set using a random sample of 7 papers and verified by experts in pharmacoepidemiology (DH) and analysis (XC). A single author (CD) will independently

For peer review only

extract data on the validation set until 80% agreement is achieved, at which stage they will continue with data extraction. A second reviewer (PS) will check the accuracy of all data extractions, and a core team (DH, CD, PS, XC) will meet regularly to discuss each study, ensure it meets the inclusion criteria, and the main messages that it provides. The broader study team will meet less frequently to address issues arising and ensure data is categorised in a meaningful way that helps to inform decision making.

All data will be made publicly available via our study's OSF page (https://osf.io/m4cbf/).

ASSESSMENT OF RISK OF BIAS

We aim to describe the evolution of the literature and will therefore not conduct a formal assessment of the risk of bias in the included studies. However, all included studies in the VIEW-hub database must meet a minimal set of quality criteria, and while this does not mean that they are free of bias, the process aims to ensure a baseline level of quality.

DATA SYNTHESIS

To describe the evolution of RWS of COVID-19 VE over the course of the pandemic, we will use descriptive statistics to quantify study characteristics – including evolution of study designs (e.g., test-negative designs, cohorts, regression discontinuity), research questions asked (e.g., comparisons of two doses vs. boosters, effectiveness and waning effect), data sources (e.g., regularly collected population data, registry data), analytic approaches (e.g., by design or form of adjustment), populations included, countries studied, outcome definitions, and event rates.

We will provide a temporal sequence of these characteristics overall, and where there are sufficient data within countries, present them visually (e.g., as annotated stacked area graphs) to establish a template that enables anticipation of study questions and therefore supports planning for data availability in future pandemics.

We plan to develop interactive visuals as outputs so that stakeholders can interrogate the data further. All data manipulation, analysis and visualisation will occur using Python and R and we will share all code via OSF.

REVIEW TEAM AND CONSULTATION

Our review team and reference group consist of content experts in review methodology, vaccine and drug effectiveness studies, biostatistics, and data science. Several have been involved directly in the conduct of VE studies during the Covid-19 pandemic and have a good working knowledge of the relevant literature. Most of the team members are actively involved in the National Health and Medical Research Council (NHMRC)-funded Centre for Research Excellence in Medicines Intelligence, which aims to accelerate real-world evidence development to inform medicines policy decision making.(22) Our reference group also

For peer review only

comprises end users in infectious diseases and pandemic management, vaccine epidemiology, and medicines and vaccine policy.

All authors and advisory group members have provided comment on this protocol, and the appropriateness of the research questions and data elements. The advisory group will be consulted on how best to present the data so that it is usable and helps with decision making in each member's respective area.

In addition, we anticipate that the data we collect can be used for future review automation work and improve the efficiency of research. Our advisory group also includes an expert in review methodology and automation who will provide advice on future-proofing our dataset.

ETHICS AND DISSEMINATION

As this scoping review will only include data in the public domain, ethics review is not required.

Findings of this review will be relevant to several stakeholders, including those involved in pandemic response, data infrastructure and health technology evaluation. As such, we will disseminate our findings in five ways: 1) working papers for policy makers in Australia; 2) open access publication of findings in peer reviewed journals; 3) presentation of findings at local and international infectious disease, vaccine, health systems and health management conferences; 4) online interactive visual to allow interrogation of the extracted data; and 5) open access to our data, code, and preprints via OSF.

PATIENT AND PUBLIC INVOLVEMENT

None.

CONTRIBUTION (CREDIT AUTHOR STATEMENT)

Paulina Stehlik and David Henry conceptualised the project, acquired the funding, and are acting as project supervisors. Paulina Stehlik, Caroline Dowsett, Ximena Camacho, Michael Falster, Renly Lim, Sharifa Nasreen, Nicole Pratt, Sallie-Anne Pearson, and David Henry contributed to the methodology. Paulina Stehlik developed the resources and database and will oversee database and project management. Paulina Stehlik, David Henry and Ximena Camacho piloted the database and extraction tool and developed the validation set. Caroline Dowsett is conducting the data extraction which will be checked by Paulina Stehlik. Paulina Stehlik, Ximena Camacho and Michael Falster developed the data synthesis plan. Paulina Stehlik and David Henry wrote the original draft of this manuscript, and Paulina Stehlik, Caroline Dowsett, Ximena Camacho, Michael Falster, Renly Lim, Sharifa Nasreen, Nicole Pratt, Sallie-Anne Pearson, and David Henry all edited and reviewed the draft and final revisions.

For peer review only

AKNOWLEDGEMENTS

We would like to acknowledge the advisory group and Oyungerel Byambasuren to their feedback and comments on draft versions of this document, particularly on the methodology and which variables to collect to provide meaningful information to decision makers.

FUNDING

Medicines Intelligence Centre for Research Excellence (MI-CRE) 2022 Project Incubator Grant; The MI-CRE is supported by the National Health and Medical Research Council's Centres of Research Excellence (CRE) scheme (ID 1196900)

Renly Lim is supported by a National Health and Medical Research Council (NHMRC) Early Career Fellowship (APP1156368).

Michael Falster is supported by a Future Leader Fellowship from the National Heart Foundation of Australia (ID: 105609).

Ximena Camacho is supported by a NHMRC Postgraduate Scholarship (ID: 2005259).

CONFLICTS OF INTEREST

No additional conflicts of interest to declare.

REFERENCES

1. Polack FP, Thomas SJ, Kitchin N, Absalon J, Gurtman A, Lockhart S, et al. Safety and Efficacy of the BNT162b2 mRNA Covid-19 Vaccine. New England Journal of Medicine. 2020;383(27):2603-15.

2. Voysey M, Clemens SAC, Madhi SA, Weckx LY, Folegatti PM, Aley PK, et al. Safety and efficacy of the ChAdOx1 nCoV-19 vaccine (AZD1222) against SARS-CoV-2: an interim analysis of four randomised controlled trials in Brazil, South Africa, and the UK. The Lancet. 2021;397(10269):99-111.

3. Xiao H, Vaidya R, Liu F, Chang X, Xia X, Unger JM. Sex, Racial, and Ethnic Representation in COVID-19 Clinical Trials: A Systematic Review and Meta-analysis. JAMA Internal Medicine. 2023;183(1):50-60.

4. Haider N, Hasan MN, Guitian J, Khan RA, McCoy D, Ntoumi F, et al. The disproportionate case–fatality ratio of COVID-19 between countries with the highest vaccination rates and the rest of the world. IJID Regions. 2023;6:159-66.

5. Booth A, Reed AB, Ponzo S, Yassaee A, Aral M, Plans D, et al. Population risk factors for severe disease and mortality in COVID-19: A global systematic review and meta-analysis. PLOS ONE. 2021;16(3):e0247461.

6. Swift B, Jain L, White C, Chandrasekaran V, Bhandari A, Hughes DA, et al. Innovation at the Intersection of Clinical Trials and Real-World Data Science to Advance Patient Care. Clinical and Translational Science. 2018;11(5):450-60.

7. Henry D, Stehlik P, Camacho X, Pearson SA. Access to routinely collected data for population health research: experiences in Canada and Australia. Australian and New Zealand Journal of Public Health. 2018;42(5):430-3.

8. Henry DA, Jones MA, Stehlik P, Glasziou PP. Effectiveness of COVID-19 vaccines: findings from real world studies. Medical Journal of Australia. 2021;215(4):149-51.e1.

For peer review only

9. Huang Z, Xu S, Liu J, Wu L, Qiu J, Wang N, et al. Effectiveness of inactivated and Ad5-nCoV COVID-19 vaccines against SARS-CoV-2 Omicron BA. 2 variant infection, severe illness, and death. BMC Medicine. 2022;20(1):400.

10. Prasad S, Kalafat E, Blakeway H, Townsend R, O'Brien P, Morris E, et al. Systematic review and meta-analysis of the effectiveness and perinatal outcomes of COVID-19 vaccination in pregnancy. Nature Communications. 2022;13(1):2414.

11. Rahmani K, Shavaleh R, Forouhi M, Disfani HF, Kamandi M, Oskooi RK, et al. The effectiveness of COVID-19 vaccines in reducing the incidence, hospitalization, and mortality from COVID-19: A systematic review and meta-analysis. Frontiers in Public Health. 2022;10.

12. Kow CS, Hasan SS. Real-world effectiveness of BNT162b2 mRNA vaccine: a meta-analysis of large observational studies. Inflammopharmacology. 2021;29(4):1075-90.

13. Tan ST, Kwan AT, Rodríguez-Barraquer I, Singer BJ, Park HJ, Lewnard JA, et al. Infectiousness of SARS-CoV-2 breakthrough infections and reinfections during the Omicron wave. Nature Medicine. 2023;29(2):358-65.

14. Shi X, Li KQ, Mukherjee B. Current Challenges With the Use of Test-Negative Designs for Modeling COVID-19 Vaccination and Outcomes. American Journal of Epidemiology. 2023;192(3):328-33.

15. UK Health Security Agency working with the Medicines & Healthcare products Regulatory Agency. COVID-19 vaccine surveillance report (2 March 2023). In: Care DoHaS, editor. gov.uk2023.

16. Mukherjee B. Being a Public Health Statistician During a Global Pandemic. Statistical Science. 2022;37(2):270-7, 8.

17. The true death toll of COVID-19: estimating global excess mortality: World Health Organization; [cited 2023 March 7]. Available from: https://www.who.int/data/stories/the-true-death-toll-of-covid-19-estimating-global-excess-mortality.

18. DJ Peters M, Godfrey C, McInerney P, Munn Z, Tricco AC, Khalil H. Chapter 11: Scoping Reviews. 2020. In: JBI Manual for Evidence Synthesis [Internet]. https://synthesismanual.jbi.global: JBI.

19. Tricco AC, Lillie E, Zarin W, O'Brien KK, Colquhoun H, Levac D, et al. PRISMA Extension for Scoping Reviews (PRISMA-ScR): Checklist and Explanation. Annals of Internal Medicine. 2018;169(7):467-73.

20. International Vaccine Access Center (IVAC), Johns Hopkins Bloomberg School of Public Health. VIEW-hub. 2023.

21. International Vaccine Access Center, Johns Hopkins Bloomberg School of Public Health, World Health Organization, Coalition for Epidemic Preparedness Innovation. Results of COVID-19 Vaccine Effectiveness & Impact Studies: An Ongoing Systematic Review. Methods.: International Vaccine Access Center (IVAC), Johns Hopkins Bloomberg School of Public Health. VIEW-hub., ; 2022 [updated 10 September 2022. Available from: https://view-hub.org/sites/default/files/2022-09/COVID19_VE_Lit_Review_Methods.pdf.

22. Pratt N, Camacho X, Vajdic C, Degenhardt L, Laba T-L, Hillen J, et al. The Medicines Intelligence Centre of Research Excellence: Co-creating real-world evidence to support the evidentiary needs of Australian medicines regulators and payers. International Journal of Population Data Science. 2021;6(3).

For peer review only

Results of COVID-19 Vaccine Effectiveness & Impact Studies: An Ongoing Systematic Review

Methods

Updated September 10, 2022

For peer review only

Prepared by:

International Vaccine Access Center,
Johns Hopkins Bloomberg School of Public Health

and

World Health Organization

and

Coalition for Epidemic Preparedness Innovations

For comments or questions, please contact: Melissa Higdon at mhigdon@jhu.edu.

Methods for Vaccine Effectiveness (VE) Literature Presented on VIEW-hub and in the Weekly Summary Tables, Visualizations, and Summaries of Policy Gaps

Literature Search

A search of the preprint, and published literature for COVID-19 Vaccine Effectiveness studies is conducted weekly. See Appendix for literature search criteria.

Inclusion Criteria for data abstraction

Title and abstract review are conducted to identify relevant studies for full-text review. During full-text review, a study must contain at least one vaccine effectiveness estimate that meets all the following criteria to be included. This is done to ensure a baseline level of quality and/or comparability of VE estimates, though this does not imply that all studies are Grade A/have minimal risk of bias nor that all excluded studies are of poor quality.

- Published or preprint studies or reports with adequate scientific details. The information cannot come just from a press release, presentations, nor media.
- VE estimates must have confidence intervals around the estimate, except in those cases where it is unable to be calculated.
- All studies must include persons with and without the clinical outcome under investigation and with and without vaccination. Thus, this excludes case only studies, such as impact studies, or those evaluating risk of progression are excluded. This criterion does not apply to transmission studies which evaluate vaccine effectiveness against secondary infection from vaccinated and unvaccinated SARS-CoV-2 cases only, or to booster dose VE studies in which the reference group is persons having completed primary series vaccination.
- The study cannot have a modeled comparison group nor compare to a historical cohort.
- Due to the effect of confounders, the study design should account for confounding and/or the VE estimate should be adjusted or state adjustment made no difference.
- All outcomes must be lab confirmed. As COVID-19 does not have a specific syndrome, studies with syndromic outcomes are excluded.
- At least 90% of participants must have a confirmed vaccination status, rather than relying on recall.
- The study must provide a VE estimate for one vaccine, not for multiple vaccines combined. The exceptions are for 1) studies assessing the combined VE of BNT162b2 (Pfizer) and mRNA-1273 (Moderna) vaccines, 2) studies of heterologous schedules but all participants included in a VE estimate should receive the same brands of vaccines in the same order, and 3) studies of vaccine effectiveness against transmission (due to the scarcity of transmission studies).
- No significant bias that likely affects results
- Cannot include day 0-12 in unvaccinated definition
- Cannot compare to early post vaccination to calculate VE (e.g. day 0-12 vs day 12-21)

A summary table of the main results of studies meeting inclusion criteria can be found on the VIEW-hub Resources page (https://view-hub.org/resources).

For peer review only

Inclusion Criteria for Forest Plots Posed on VIEW-Hub

The VE estimates from eligible studies are plotted in figures. The estimates plotted are a subset of the estimates abstracted from the systematic literature review of those studies meeting additional eligibility criteria. Because a single study can include many VE estimates where the same data appear in more than one VE estimate (e.g., all ages and also separately by age group), criteria are applied to prioritize which to plot in an effort to not overrepresent the amount of evidence that exists for each vaccine. The following criteria are used to determine which VE estimates are displayed in the summary forest plots located on the VIEW-hub resources page (https://view-hub.org/resources):

- Complete vaccination is defined as ≥7 days post final dose; partial vaccination is defined as ≥14 days post first dose of a 2-dose vaccine (current forest plots display VE estimates for complete primary series, first booster dose, and second booster dose; partial vaccination is no longer shown).
- If a study reports results for the same outcome for both combined and individual vaccines, only individual vaccine VE estimates are displayed. This criterion only apples to studies evaluating VE of BNT162b2 (Pfizer) mRNA-1273 (Moderna) vaccines.
- If a study reports results from 2 different evaluation designs (e.g. test-negative design and cohort design) on the same population, VE estimates from the primary analysis only are displayed.
- If a study reports VE estimates for the same disease outcome for different populations, the general population VE estimate is displayed when available. If a general population estimate is not available, the VE from each population is displayed (exception is if there are estimates for similar age groups in which case the more stable VE estimate will be displayed).
- If a study reports VE estimates on more than one 'severe' disease outcome (e.g. 'severe disease', 'hospitalization', and 'ICU admission'), the more inclusive disease outcome including a larger population is displayed. These different types of severe outcomes are labeled as 'severe disease' in the plots, however it is important to keep in mind that the definition of severe disease varies and may explain some differences in VE estimates for severe disease outcomes.
- If a study reports VE estimates for a specific regimen and population at different time intervals since vaccination, the earliest interval of peak VE is selected for the vaccine-specific forest plots (with an exception for the plots on duration of vaccine effectiveness in which multiple time points are plotted). Studies that report only one VE estimate for a specific regimen and population (i.e. at ≥ 14 days post final dose) are included in the vaccine-specific plots and denoted with a '+' after the reference id if the time interval post-vaccination over which VE is measured extends beyond 4 months.

Additional notes

Estimates from mutually exclusive populations in a study can be displayed in the same plot resulting in instances when more than one estimate from a study is plotted (e.g., a study includes VE estimates from two distinct age groups or estimates for different variants).

For studies that report adjusted odds ratios, risk ratios, or rate ratios instead of vaccine effectiveness estimates, VE is calculated as 1 minus the reported effect estimate and multiplying by 100.

For peer review only - http://bmjopen.bmj.com/site/about/guidelines.xhtml

Reference numbers are included for each VE estimate displayed so users can identify when a study is represented more than once within a plot. More information on each reference can be found in the weekly literature review summary table located on VIEW-HUB (https://view-hub.org/resources).

Vaccine Effectiveness Studies Database

See accompanying PDF ('CEPI_COVID19VaccineEffectiveness.pdf') of detailed data collection forms for COVID-19 vaccine effectiveness studies. The complete vaccine effectiveness studies database will be made available to CEPI at anytime upon request. In addition, a summary PDF file of all abstracted VE estimates is available on the VIEW-hub Resources page. The same information is also available in a downloadable filterable Excel file ('COVID-19 Vaccine Effectiveness Results Dataset'). These materials are available to the public and updated weekly.

Planned and Ongoing Studies presented on VIEW-hub and summaries of policy gaps

In order to gather information on planned and ongoing studies, a survey was shared with persons conducting and/or funding studies. Data was requested specifically on studies that have completed protocol development to help obtain higher quality data as studies that are still in protocol development are subject to more changes. This data has been compiled by WHO and some key information and summaries of what is planned/ongoing are provided on View Hub and WHO's website.

For peer review only

Appendix: Literature Search Terms

PubMed:

("COVID-19"[tw] OR "COVID 19"[tw] OR "COVID19"[tw] OR "COVID2019"[tw] OR "COVID 2019"[tw] OR "COVID-2019"[tw] OR "novel coronavirus"[tw] OR "new coronavirus"[tw] OR "novel corona virus"[tw] OR "new corona virus"[tw] OR "SARS-CoV-2"[tw] OR "SARSCoV2"[tw] OR "SARS-CoV2"[tw] OR "2019nCoV"[tw] OR "2019-nCoV"[tw] OR "2019 coronavirus"[tw] OR "2019 corona virus"[tw] OR "coronavirus disease 2019"[tw] OR "severe acute respiratory syndrome coronavirus 2"[nm] OR "severe acute respiratory syndrome coronavirus 2"[tw] OR "sars-coronavirus-2"[tw] OR "coronavirus disease 2019"[tw] OR "corona virus disease 2019"[tw])

AND

("COVID-19 Vaccines"[Mesh] OR “COVID-19 vaccine”[tiab] OR "mRNA-1273 vaccine" [Supplementary Concept] OR “mRNA-1273 vaccine”[tiab] OR “mRNA vaccine”[tiab] OR “mRNA COVID-19 vaccines”[tiab] OR "ChAdOx1 COVID-19 vaccine" [Supplementary Concept] OR "Ad5-nCoV vaccine" [Supplementary Concept] OR “Ad5-nCoV”[tiab] OR "Covid-19 aAPC vaccine" [Supplementary Concept] OR "Ad26.COV2.S vaccine" [Supplementary Concept] OR "Ad26.COV2.S vaccine"[tiab] OR “adenoviral vector vaccine”[tiab] OR "BNT162 vaccine" [Supplementary Concept] OR “BNT162b2”[tiab] OR “BNT162”[tiab] OR “CoronaVac” [tiab] OR “vaccin*”[tiab])

AND

("Clinical Trial, Phase IV" [Publication Type] OR "Controlled Clinical Trial" [Publication Type] OR "Randomized Controlled Trial" [Publication Type] OR "Case-Control Studies"[Mesh] OR "Retrospective Studies"[Mesh] OR “Retrospective”[tiab] OR "Cohort Studies"[Mesh] OR "Prospective Studies"[Mesh] OR “Prospective”[tiab] OR "Longitudinal Studies"[Mesh] OR "Follow-Up Studies"[Mesh] OR “Follow-up studies”[tiab] OR “cohort”[tiab] OR "test negative"[tiab] OR “Observational cohort”[tiab] OR “Test-negative design”[tiab] OR"RCT"[tiab] OR "randomized"[tiab] OR "randomised"[tiab] OR "randomly allocated"[tiab] OR “case-control”[tiab] OR “real-world effectiveness”[tiab] OR “effectiveness”[tiab] OR “association”[tiab] OR “impact”[tiab] OR “vaccine impact”[tiab]) NOT ("Clinical Trial, Phase I" [Publication Type] OR "Clinical Trial, Phase II" [Publication Type])
NOT (“animals”[mesh] NOT (“animals”[mesh] AND “humans”[mesh]))

Embase:

('COVID-19' OR 'COVID 19' OR 'COVID19' OR 'COVID2019' OR 'COVID 2019' OR 'COVID-2019' OR 'novel coronavirus' OR 'new coronavirus' OR 'novel corona virus' OR 'new corona virus' OR 'SARS-CoV-2' OR 'SARSCoV2' OR 'SARS-CoV2' OR '2019nCoV' OR '2019-nCoV' OR '2019 coronavirus' OR '2019 corona virus' OR 'coronavirus disease 2019' OR 'severe acute respiratory

For peer review only

syndrome coronavirus 2'/exp OR 'severe acute respiratory syndrome coronavirus 2' OR 'sars-coronavirus-2' OR 'coronavirus disease 2019'/exp OR 'coronavirus disease 2019' OR 'corona virus disease 2019')

AND

('SARS-CoV-2 vaccine'/exp OR 'COVID-19 vaccine':ti,ab OR 'mRNA-1273 vaccine'/exp OR 'mRNA-1273 vaccine':ti,ab OR 'mRNA vaccine':ti,ab OR 'mRNA COVID-19 vaccines':ti,ab OR 'ChAdOx1 ncov 19'/exp OR 'Ad5 nCoV vaccine'/exp OR 'Ad5-nCoV':ti,ab OR 'Covid-19 aAPC vaccine':ti,ab OR 'Ad26.COV2.S vaccine'/exp OR 'Ad26.COV2.S vaccine':ti,ab OR 'adenoviral vector vaccine':ti,ab OR 'BNT 162 vaccine'/exp OR 'BNT162b2':ti,ab OR 'BNT162':ti,ab OR 'CoronaVac'/exp OR 'coronavac':ti,ab OR 'vaccin*':ti,ab)

AND

('phase 4 clinical trial'/exp OR 'Controlled Clinical Trial'/exp OR 'Randomized Controlled Trial'/exp OR 'Case Control Study'/exp OR 'Retrospective Study'/exp OR 'Retrospective':ti,ab OR 'Cohort analysis'/exp OR 'Prospective Study'/exp OR 'Prospective':ti,ab OR 'Longitudinal Study'/exp OR 'Follow Up'/exp OR 'Follow-up study':ti,ab OR 'cohort':ti,ab OR 'test negative':ti,ab OR 'Observational cohort':ti,ab OR 'postmarketing surveillance'/exp OR 'postmarketing surveillance':ti,ab OR 'Test-negative design':ti,ab OR 'RCT':ti,ab OR 'randomized':ti,ab OR 'randomised':ti,ab OR 'randomly allocated':ti,ab OR 'case-control':ti,ab OR 'real-world effectiveness':ti,ab OR 'effectiveness':ti,ab OR 'association':ti,ab) NOT ('phase 1 clinical trial'/exp OR 'phase 2 clinical trial'/exp)
NOT ('animal'/exp NOT ('animal'/exp AND 'human'/exp))
NOT 'conference abstract'/it

<u>WHO COVID database:</u>

("COVID-19 Vaccines" OR "COVID-19 vaccine" OR "mRNA-1273 vaccine" OR "mRNA vaccine" OR "mRNA COVID-19 vaccines" OR "ChAdOx1 COVID-19 vaccine" OR "Ad5-nCoV" OR "Covid-19 aAPC vaccine" OR "Ad26.COV2.S vaccine" OR "adenoviral vector vaccine" OR "BNT162b2" OR "BNT162" OR "CoronaVac" OR vaccin*)

AND

("Phase IV" OR "Controlled Clinical Trial" OR "Randomized Controlled Trial" OR "Case-Control Studies" OR "Retrospective" OR "Cohort Studies" OR "Prospective" OR "Longitudinal Studies" OR "Follow-Up Studies" OR "Follow-up study" OR "cohort" OR "test negative" OR "Observational cohort" OR "Test-negative design" OR "RCT" OR "randomized" OR "randomised" OR "randomly allocated" OR "case-control" OR "real-world effectiveness" OR "effectiveness" OR "association") AND NOT ("Phase I" OR "Phase II")

<u>SCOPUS:</u>

For peer review only

INTERNATIONAL VACCINE ACCESS CENTER

World Health Organization

CEPI

TITLE-ABS-KEY(“novel coronavir*” OR “novel corona virus*” OR “2019 coronavirus” OR betacoronavir* OR covid19 OR "covid 19" OR ncov OR "CoV 2" OR cov2 OR sarscov2 OR sars-cov OR sarscov OR 2019ncov OR 2019-nCoV OR "novel CoV" OR “coronavirus infections”) AND TITLE-ABS-KEY(Vaccin* AND (effectiveness OR efficacy OR protection*) AND (postmarketing OR approved OR (post* W/5 approval) OR "real world" OR "phase IV" OR "phase 4" OR observational OR longitudinal OR spread OR transmission OR (rate* W/5 infection*) OR (reduc* W/5 infection*) OR "general population"))

Web of Science:

(TI=(covid-19 vaccine effectiveness)) OR AB=(covid-19 vaccine effectiveness)

medRxiv, bioRxiv, SSRN, Europe PMC, Research Square, Knowledge Hub:

“COVID-19 vaccine effectiveness” OR “COVID-19 vaccine efficacy”

In addition to the above databases, MMWR, and Eurosurveillance are hand-searched weekly for new studies meeting eligibility criteria.

For peer review only

Preferred Reporting Items for Systematic reviews and Meta-Analyses extension for Scoping Reviews (PRISMA-ScR) Checklist

SECTION	ITEM	PRISMA-ScR CHECKLIST ITEM	REPORTED ON PAGE #
**TITLE**			
Title	1	Identify the report as a scoping review.	1
**ABSTRACT**			
Structured summary	2	Provide a structured summary that includes (as applicable): background, objectives, eligibility criteria, sources of evidence, charting methods, results, and conclusions that relate to the review questions and objectives.	2
**INTRODUCTION**			
Rationale	3	Describe the rationale for the review in the context of what is already known. Explain why the review questions/objectives lend themselves to a scoping review approach.	4-5
Objectives	4	Provide an explicit statement of the questions and objectives being addressed with reference to their key elements (e.g., population or participants, concepts, and context) or other relevant key elements used to conceptualize the review questions and/or objectives.	5
**METHODS**			
Protocol and registration	5	Indicate whether a review protocol exists; state if and where it can be accessed (e.g., a Web address); and if available, provide registration information, including the registration number.	2
Eligibility criteria	6	Specify characteristics of the sources of evidence used as eligibility criteria (e.g., years considered, language, and publication status), and provide a rationale.	6-7
Information sources*	7	Describe all information sources in the search (e.g., databases with dates of coverage and contact with authors to identify additional sources), as well as the date the most recent search was executed.	6
Search	8	Present the full electronic search strategy for at least 1 database, including any limits used, such that it could be repeated.	Supp 1
Selection of sources of evidence†	9	State the process for selecting sources of evidence (i.e., screening and eligibility) included in the scoping review.	7-8
Data charting process‡	10	Describe the methods of charting data from the included sources of evidence (e.g., calibrated forms or forms that have been tested by the team before their use, and whether data charting was done independently or in duplicate) and any processes for obtaining and confirming data from investigators.	7-8
Data items	11	List and define all variables for which data were sought and any assumptions and simplifications made.	7-8
Critical appraisal of individual sources of evidence§	12	If done, provide a rationale for conducting a critical appraisal of included sources of evidence; describe the methods used and how this information was used in any data synthesis (if appropriate).	8

For peer review only

SECTION	ITEM	PRISMA-ScR CHECKLIST ITEM	REPORTED ON PAGE #
Synthesis of results	13	Describe the methods of handling and summarizing the data that were charted.	8
**RESULTS**			
Selection of sources of evidence	14	Give numbers of sources of evidence screened, assessed for eligibility, and included in the review, with reasons for exclusions at each stage, ideally using a flow diagram.	TBC
Characteristics of sources of evidence	15	For each source of evidence, present characteristics for which data were charted and provide the citations.	TBC
Critical appraisal within sources of evidence	16	If done, present data on critical appraisal of included sources of evidence (see item 12).	TBC
Results of individual sources of evidence	17	For each included source of evidence, present the relevant data that were charted that relate to the review questions and objectives.	TBC
Synthesis of results	18	Summarize and/or present the charting results as they relate to the review questions and objectives.	TBC
**DISCUSSION**			
Summary of evidence	19	Summarize the main results (including an overview of concepts, themes, and types of evidence available), link to the review questions and objectives, and consider the relevance to key groups.	TBC
Limitations	20	Discuss the limitations of the scoping review process.	TBC
Conclusions	21	Provide a general interpretation of the results with respect to the review questions and objectives, as well as potential implications and/or next steps.	TBC
**FUNDING**			
Funding	22	Describe sources of funding for the included sources of evidence, as well as sources of funding for the scoping review. Describe the role of the funders of the scoping review.	10

JBI = Joanna Briggs Institute; PRISMA-ScR = Preferred Reporting Items for Systematic reviews and Meta-Analyses extension for Scoping Reviews.

* Where *sources of evidence* (see second footnote) are compiled from, such as bibliographic databases, social media platforms, and Web sites.

† A more inclusive/heterogeneous term used to account for the different types of evidence or data sources (e.g., quantitative and/or qualitative research, expert opinion, and policy documents) that may be eligible in a scoping review as opposed to only studies. This is not to be confused with *information sources* (see first footnote).

‡ The frameworks by Arksey and O'Malley (6) and Levac and colleagues (7) and the JBI guidance (4, 5) refer to the process of data extraction in a scoping review as data charting.

§ The process of systematically examining research evidence to assess its validity, results, and relevance before using it to inform a decision. This term is used for items 12 and 19 instead of "risk of bias" (which is more applicable to systematic reviews of interventions) to include and acknowledge the various sources of evidence that may be used in a scoping review (e.g., quantitative and/or qualitative research, expert opinion, and policy document).

From: Tricco AC, Lillie E, Zarin W, O'Brien KK, Colquhoun H, Levac D, et al. PRISMA Extension for Scoping Reviews (PRISMAScR): Checklist and Explanation. Ann Intern Med. 2018;169:467–473. doi: 10.7326/M18-0850.

For peer review only - http://bmjopen.bmj.com/site/about/guidelines.xhtml

EVOLution of the data and methods in real world COVID-19 Vaccine Effectiveness studies on mortality: A Scoping Review Protocol

Journal:	*BMJ Open*
Manuscript ID	bmjopen-2023-079071.R5
Article Type:	Protocol
Date Submitted by the Author:	22-Feb-2024
Complete List of Authors:	Stehlik, Paulina; Griffith University Faculty of Health, School of Pharmacy and Medical Sciences; Bond University Faculty of Health Sciences and Medicine, Institute for Evidence-Based Healthcare<br>Dowsett, Caroline; Griffith University Faculty of Health, School of Pharmacy and Medical Sciences<br>Camacho, Ximena; University of New South Wales Medicine & Health, School of Population Health<br>Falster, Michael; University of New South Wales Medicine & Health, School of Population Health<br>Lim, Renly; University of South Australia Division of Health Sciences, Quality Use of Medicines and Pharmacy Research Centre<br>Nasreen, Sharifa; SUNY Downstate Health Sciences University School of Public Health<br>Pratt, Nicole; University of South Australia Clinical & Health Sciences Academic Unit, Quality Use of Medicines and Pharmacy Research Centre<br>Pearson, Sallie-Anne; University of New South Wales, School of Population Health<br>Henry, David; Bond University Faculty of Health Sciences and Medicine, Institute for Evidence-Based Healthcare; University of New South Wales, School of Population Health
**Primary Subject Heading**:	Infectious diseases
Secondary Subject Heading:	Epidemiology, Research methods, Public health
Keywords:	COVID-19, STATISTICS & RESEARCH METHODS, Protocols & guidelines < HEALTH SERVICES ADMINISTRATION & MANAGEMENT, INFECTIOUS DISEASES, Mortality, PUBLIC HEALTH

SCHOLARONE™ Manuscripts

EVOLution of the data and methods in real world COVID-19 Vaccine Effectiveness studies on mortality: A Scoping Review Protocol

Short title: EVOLVE ScR

FORWARD

AUTHORS

Paulina Stehlik, School of Pharmacy and Medical Sciences, Griffith University; Adjunct Faculty of Health Sciences and Medicine, Bond University*

Caroline Dowsett, School of Pharmacy and Medical Sciences, Griffith University.

Ximena Camacho, School of Population Health, Faculty of Medicine and Health, University of New South Wales

Michael Falster, School of Population Health, Faculty of Medicine and Health, University of New South Wales

Renly Lim, Quality Use of Medicines and Pharmacy Research Centre, UniSA Clinical and Health Sciences, University of South Australia

Sharifa Nasreen, Epidemiology and Biostatistics, School of Public Health, SUNY Downstate Health Sciences University

Nicole Pratt, Quality Use of Medicines and Pharmacy Research Centre, UniSA Clinical and Health Sciences, University of South Australia

Sallie-Anne Pearson, School of Population Health, Faculty of Medicine and Health, University of New South Wales

David Henry, Honorary/ Adjunct, Bond University and University of New South Wales

CORRESPONDING AUTHOR

For peer review only

Dr Paulina Stehlik, School of Pharmacy and Medical Sciences, Griffith University; Adjunct Faculty of Health Sciences and Medicine, Bond University

Email: p.stehlik@griffith.edu.au

ADVISORY GROUP

Bette Liu, UNSW National Centre for Immunisation Research and Surveillance

Andrew Wilson, PBAC advisory committee chair; Director of the Menzies Centre for Health Policy, Menzies Centre for Health Policy, School of Public Health

Kylie Alcorn, A/Director Infectious Diseases, Infectious Disease Department, Gold Coast Health

Alexandra Bannach-Brown, Charité - Universitätsmedizin Berlin

ABSTRACT

Background: Early evidence on COVID-19 vaccine efficacy came from randomised trials. Many important questions subsequently about vaccine effectiveness (VE) have been addressed using real-world studies (RWS) and have informed most vaccination policies globally. As the questions about VE have evolved during the pandemic so have data, study design and analytical choices. This scoping review aims to characterise this evolution and provide insights for future pandemic planning – specifically, what kinds of questions are asked at different stages of a pandemic, and what data infrastructure and methods are used?

Methods and analysis: We will identify relevant studies in the Johns Hopkins Bloomberg School of Public Health VIEW-hub database, which curates both published and pre-print VE RWS identified from PubMed, Embase, Scopus, Web of Science, the WHO COVID Database, MMWR, Eurosurveillance, medRxiv, bioRxiv, SSRN, Europe PMC, Research Square, and Knowledge Hub, and Google. We will include RWS of COVID-19 VE that reported COVID-19-specific or all-cause mortality (coded as "death" in the "effectiveness studies" dataset).

Information on study characteristics; study context; data sources; design and analytic methods that address confounding will be extracted by single reviewer and checked for accuracy and discussed in a small group setting by methodological and analytic experts. A timeline mapping approach will be used to capture the evolution of this body of literature.

By describing the evolution of RWS of VE through the COVID-19 pandemic, we will help identify options for VE studies and inform policy makers on the minimal data and analytic infrastructure needed to support rapid RWS of VE in future pandemics and of health care strategies more broadly.

For peer review only - http://bmjopen.bmj.com/site/about/guidelines.xhtml

Ethics and dissemination: As data is in the public domain, ethical approval is not required. Findings of this study will be disseminated through peer-reviewed publications, conference presentations, and working-papers to policy makers.

Registration: https://doi.org/10.17605/OSF.IO/ZHDKR

STRENGTHS AND LIMITATIONS

- We will use a comprehensive curated database (Johns Hopkins Bloomberg School of Public Health VIEW-hub that compiles relevant studies on a weekly basis from multiple databases, preprint servers and the grey literature.
- While use of a curated database may lead to some studies being missed, this is unlikely to change the overall findings of this scoping review.
- All extraction will be conducted by a single author to ensure consistency in extraction and checked by a second author to ensure accuracy.
- Weekly group discussions about the individual studies and coding of data will strengthen data integrity.
- End users have been involved in the design of this study and will continue to be consulted throughout its conduct.

For peer review only

INTRODUCTION

The COVID-19 pandemic has been unprecedented in terms of its direct health impacts and disruption of many aspects of modern society. It has also been remarkable in the speed with which scientists and industry collaborated in the production and testing of a range of vaccines.

It became apparent quickly that the COVID-19 vaccines did not stimulate sterilising immunity but provided protection against severe illness and death, most importantly in those with underlying risk factors.(1, 2) The randomised trials that formed the evidence base for the initial deployment of vaccines included few subjects who were elderly, very young, pregnant, had immunodeficiency or severe co-morbidity states.(3) Although quite large, the randomised trials documented few deaths and could not provide precise estimates of the effectiveness of the vaccines in reducing COVID-related and all-cause mortality.

The subsequent evaluation of vaccine effectiveness (VE) using controlled observational studies has been complicated by changes in the infectiousness and virulence of the SARS-CoV-2 virus, and rising background levels of vaccine-induced or naturally acquired immunity. Case fatality rates have fallen substantially, particularly in highly vaccinated countries.(4) Deaths are now concentrated in a group of older patients, those with obesity and those who have serious comorbidities or are immunocompromised.(5) This rapidly changing landscape created a need for continuous 'real-world' studies (RWS) of vaccine effectiveness in susceptible groups, against emerging viral variants and after repeated vaccine doses.(6) These studies use data collected outside of clinical trial settings to define exposures, endpoints and relevant covariates. This is achieved by analysing data from electronic medical records, administrative records, death registries and registries established specifically to record infection status and vaccine receipt.(6)

Most VE studies of COVID-19 vaccines have employed large population-scale linked routinely collected datasets. However, countries have varied in the timeliness of their response to this major challenge. In some countries, for instance Israel and UK, collaborations between researchers, health service providers and government agencies enabled rapid analyses of large datasets using sophisticated techniques to adjust for confounding and other sources of bias. In contrast, other countries, for instance Australia and Aotearoa/New Zealand, were slow to conduct effectiveness studies, in part because of low infection rates early in the pandemic, and in Australia because of difficulties in accessing the necessary linked datasets.(7, 8)

Systematic reviews of VE studies have concentrated, appropriately, on the vaccines' ability to prevent serious illness and death.(9-12) They have been consistent in confirming that multiple doses of the available vaccines have been associated with large reductions in mortality, with quite rapid waning (over months) in protection, mandating a need for

For peer review only

repeated booster doses. As the impacts of vaccines on infection and transmission have been limited and transient,(13) it diminishes the value of infection as the principal study endpoint. The decline in PCR testing and registration of antigen test results have reduced the value of test results as the basis for test negative designs.(14, 15) The nature of COVID-19 related hospitalisations has changed during the pandemic with an increase in incidental findings of infection through routine testing of patients admitted for other reasons.(15) On the other hand, there has been an increasing focus on excess all-cause mortality as a measure of the success of countries in controlling the spread of the virus and mitigating its negative impacts on healthcare systems.(16, 17)

The COVID-19 pandemic has been a historic event that we must learn from. The rapid deployment of vaccines, followed by studies of their effectiveness, represents the largest and most important healthcare intervention in recent history and one that was evaluated largely using non-randomised studies. The sense of pandemic urgency led to rapid development of strategies to establish datasets, designs, and analytic approaches. This evolution of study questions, data designs and methods through the course of the pandemic provides a unique learning opportunity for policy makers and researchers alike.

We plan to conduct a scoping review of the evidence base on real world Covid-19 vaccine effectiveness, focusing on studies that report on death as an outcome, to document this evolution. Specifically we will explore: how policy-relevant questions changed over the course of the pandemic, and how these affected the choices of data sources, designs, and analytical methods. By analysing these we hope to provide information that is useful to the following stakeholders:

1. Policy makers and health system managers: by indicating what datasets will have to be created de novo and the need for linkage to existing routinely collected data in responding to future pandemics.
2. Clinicians and laboratory scientists: by identifying the disease manifestations and clinical and demographic vulnerability factors that will be required to inform the designs and analyses needed to evaluate the effectiveness of vaccines and other interventions, how these may change over the course of a future pandemic, and how the clinical community can advocate for the appropriate data elements to be linked and made available to researchers.
3. Data scientists and methodologists: to provide guidance as to study designs, analytical and adjustment techniques that are most often used in providing rapid estimates of VE early in a future pandemic; to advocate for the data elements required to deal with confounding to be collected and available in a linked analysable form.
4. Vaccine manufacturers: to understand better the post licensing requirements for vaccines and pharmaceutical products under pandemic conditions and contribute appropriately to the necessary evaluations.

For peer review only

5. The pharmacoepidemiology community generally: the rapid evaluation of vaccine effectiveness during the COVID-19 pandemic provides lessons for the timely investigation of a range of pharmaceutical treatments for emerging health threats.

METHODS

We will conduct a scoping review, following the methods published by the Joanna Briggs Institute(18) and report the results according to the Preferred Reporting Items for Systematic Reviews and Meta-Analyses statement for scoping review (PRISMA-ScR).(19) This scoping review is registered with the Open Science Framework (OSF; https://doi.org/10.17605/OSF.IO/ZHDKR). Data extraction has begun (25th September 2023, after protocol registration), and will continue for approximately 6 months.

INFORMATION SOURCES AND DATA SELECTION

We will retrieve relevant studies from the VIEW-hub database,(20) maintained by Johns Hopkins Bloomberg School of Public Health. This database includes a wide range of study types including vaccine efficacy trials, vaccine effectiveness studies, impact studies and safety studies. As our principal aim is to describe the evolution of observational vaccine effectiveness studies using real world data, we used the VIEW-hub "effectiveness studies" dataset.

The VIEW-hub search strategy and inclusion criteria for this dataset have been described in detail elsewhere. (see Supplementary File)(21) Briefly, the "effectiveness studies" dataset includes both published and pre-print studies of vaccine effectiveness identified from PubMed, Embase, Scopus, Web of Science, the WHO COVID Database, MMWR, Eurosurveillance, medRxiv, bioRxiv, SSRN, Europe PMC, Research Square, and Knowledge Hub, as well as Google alerts for COVID-19 vaccine effectiveness studies. Studies are screened weekly by the same two epidemiologists at Johns Hopkins Bloomberg School of Public Health, and the following data elements are extracted for studies included in the dataset: study author, title, date published, link to paper, country of origin, vaccine studied, variant studied, population, study start and end date, and outcomes of interest. Studies in the dataset can be filtered by the vaccine, variant, outcomes, study population and region variables through drop-down menus.

Studies must also meet minimum criteria for causal inference studies using real-world data. The studies must include both vaccinated and unvaccinated (or other control) subjects, drawn from a comparable time period, capturing the relevant endpoints in both groups, having a secure record of vaccination (not relying on recall) and be free of obvious major methodological flaws. The latter judgment was not based on a strict risk of bias assessment.

For peer review only

To identify studies in the VIEW-hub's "effectiveness studies" dataset that examine mortality (either all-cause or cause-specific) we will use the drop-down menu feature to select study outcomes coded as "death". No additional eligibility criteria will be applied.

At the time of writing this protocol (1 Aug 2023) the VIEW-hub database lists 495 observational studies of vaccine effectiveness from 50 countries, and 92 (~19%) list "death" as an endpoint.

DATA EXTRACTION

We will extract data on:

1. *Study characteristics*: country, study design, publication status, protocol available, funding sources (including whether the study was funded by an independent source or manufacturer), study ethics approval (or waiver), consent requirements (or waiver).
2. *Study context*: reported vaccine policies in place, reported dominant viral variant at time of study.
3. *PICO-T*: inclusion and exclusion criteria, exposure (i.e. vaccine(s)) and definition of exposure, control group, outcome definitions, outcomes collection period duration of follow up and number of deaths.
4. *Data sources and additional variables*: the types of data sources used (e.g., survey, electronic medical records, administrative data), which were linked at an individual level and which were not, baseline confounders collected, and for adjusted outcomes which variables they were adjusted for;
5. *Analytical strategies to minimise bias*: methods for minimising baseline confounding (e.g., propensity score analysis, instrumental variable analysis, covariate adjustment, self-controlled designs, etc.) and further details of how the methods were implemented as appropriate, such as how the propensity score was implemented (matching, stratification or inverse probability of treatment weights) and which variables were included in the propensity score model. Additionally, we will extract details on whether a sensitivity analyses was conducted, subgroups analysed, methods used for dealing with missing data, and methods used for dealing with time varying environmental risk.

We anticipate there will be a few data points where it will be difficult to provide an exhaustive list of potential categories for some of the variables of interest *a priori*. We will therefore take an inductive approach to categorising variables such as "data sources", "inclusion criteria", and "adjustment techniques" by entering them in free text and then developing categories through group discussion.

The lead author (PS) will develop a purpose-built data-extraction form in SharePoint Lists and a blank copy of the form and data dictionary will be provided on our OSF site. PS will also develop a validation set using a random sample of 7 papers and verified by experts in pharmacoepidemiology (DH) and analysis (XC). A single author (CD) will independently

For peer review only

extract data on the validation set until 80% agreement is achieved, at which stage they will continue with data extraction. A second reviewer (PS) will check the accuracy of all data extractions, and a core team (DH, CD, PS, XC) will meet regularly to discuss each study, ensure it meets the inclusion criteria, and the main messages that it provides. The broader study team will meet less frequently to address issues arising and ensure data is categorised in a meaningful way that helps to inform decision making.

All data will be made publicly available via our study's OSF page (https://osf.io/m4cbf/).

ASSESSMENT OF RISK OF BIAS

We aim to describe the evolution of the literature and will therefore not conduct a formal assessment of the risk of bias in the included studies. However, all included studies in the VIEW-hub database must meet a minimal set of quality criteria, and while this does not mean that they are free of bias, the process aims to ensure a baseline level of quality.

DATA SYNTHESIS

To describe the evolution of RWS of COVID-19 VE over the course of the pandemic, we will use descriptive statistics to quantify study characteristics – including evolution of study designs (e.g., test-negative designs, cohorts, regression discontinuity), research questions asked (e.g., comparisons of two doses vs. boosters, effectiveness and waning effect), data sources (e.g., regularly collected population data, registry data), analytic approaches (e.g., by design or form of adjustment), populations included, countries studied, outcome definitions, and event rates.

We will provide a temporal sequence of these characteristics overall, and where there are sufficient data within countries, present them visually (e.g., as annotated stacked area graphs) to establish a template that enables anticipation of study questions and therefore supports planning for data availability in future pandemics.

We plan to develop interactive visuals as outputs so that stakeholders can interrogate the data further. All data manipulation, analysis and visualisation will occur using Python and R and we will share all code via OSF.

REVIEW TEAM AND CONSULTATION

Our review team and reference group consist of content experts in review methodology, vaccine and drug effectiveness studies, biostatistics, and data science. Several have been involved directly in the conduct of VE studies during the Covid-19 pandemic and have a good working knowledge of the relevant literature. Most of the team members are actively involved in the National Health and Medical Research Council (NHMRC)-funded Centre for Research Excellence in Medicines Intelligence, which aims to accelerate real-world evidence development to inform medicines policy decision making.(22) Our reference group also

For peer review only

comprises end users in infectious diseases and pandemic management, vaccine epidemiology, and medicines and vaccine policy.

All authors and advisory group members have provided comment on this protocol, and the appropriateness of the research questions and data elements. The advisory group will be consulted on how best to present the data so that it is usable and helps with decision making in each member's respective area.

In addition, we anticipate that the data we collect can be used for future review automation work and improve the efficiency of research. Our advisory group also includes an expert in review methodology and automation who will provide advice on future-proofing our dataset.

ETHICS AND DISSEMINATION

As this scoping review will only include data in the public domain, ethics review is not required.

Findings of this review will be relevant to several stakeholders, including those involved in pandemic response, data infrastructure and health technology evaluation. As such, we will disseminate our findings in five ways: 1) working papers for policy makers in Australia; 2) open access publication of findings in peer reviewed journals; 3) presentation of findings at local and international infectious disease, vaccine, health systems and health management conferences; 4) online interactive visual to allow interrogation of the extracted data; and 5) open access to our data, code, and preprints via OSF.

PATIENT AND PUBLIC INVOLVEMENT

None.

CONTRIBUTION (CREDIT AUTHOR STATEMENT)

Paulina Stehlik and David Henry conceptualised the project, acquired the funding, and are acting as project supervisors. Paulina Stehlik, Caroline Dowsett, Ximena Camacho, Michael Falster, Renly Lim, Sharifa Nasreen, Nicole Pratt, Sallie-Anne Pearson, and David Henry contributed to the methodology. Paulina Stehlik developed the resources and database and will oversee database and project management. Paulina Stehlik, David Henry and Ximena Camacho piloted the database and extraction tool and developed the validation set. Caroline Dowsett is conducting the data extraction which will be checked by Paulina Stehlik. Paulina Stehlik, Ximena Camacho and Michael Falster developed the data synthesis plan. Paulina Stehlik and David Henry wrote the original draft of this manuscript, and Paulina Stehlik, Caroline Dowsett, Ximena Camacho, Michael Falster, Renly Lim, Sharifa Nasreen, Nicole Pratt, Sallie-Anne Pearson, and David Henry all edited and reviewed the draft and final revisions.

For peer review only

AKNOWLEDGEMENTS

We would like to acknowledge the advisory group and Oyungerel Byambasuren to their feedback and comments on draft versions of this document, particularly on the methodology and which variables to collect to provide meaningful information to decision makers.

FUNDING

Medicines Intelligence Centre for Research Excellence (MI-CRE) 2022 Project Incubator Grant; The MI-CRE is supported by the National Health and Medical Research Council's Centres of Research Excellence (CRE) scheme (ID 1196900)

Renly Lim is supported by a National Health and Medical Research Council (NHMRC) Early Career Fellowship (APP1156368).

Michael Falster is supported by a Future Leader Fellowship from the National Heart Foundation of Australia (ID: 105609).

Ximena Camacho is supported by a NHMRC Postgraduate Scholarship (ID: 2005259).

CONFLICTS OF INTEREST

No additional conflicts of interest to declare.

For peer review only

REFERENCES

1. Polack FP, Thomas SJ, Kitchin N, Absalon J, Gurtman A, Lockhart S, et al. Safety and Efficacy of the BNT162b2 mRNA Covid-19 Vaccine. New England Journal of Medicine. 2020;383(27):2603-15.

2. Voysey M, Clemens SAC, Madhi SA, Weckx LY, Folegatti PM, Aley PK, et al. Safety and efficacy of the ChAdOx1 nCoV-19 vaccine (AZD1222) against SARS-CoV-2: an interim analysis of four randomised controlled trials in Brazil, South Africa, and the UK. The Lancet. 2021;397(10269):99-111.

3. Xiao H, Vaidya R, Liu F, Chang X, Xia X, Unger JM. Sex, Racial, and Ethnic Representation in COVID-19 Clinical Trials: A Systematic Review and Meta-analysis. JAMA Internal Medicine. 2023;183(1):50-60.

4. Haider N, Hasan MN, Guitian J, Khan RA, McCoy D, Ntoumi F, et al. The disproportionate case–fatality ratio of COVID-19 between countries with the highest vaccination rates and the rest of the world. IJID Regions. 2023;6:159-66.

5. Booth A, Reed AB, Ponzo S, Yassaee A, Aral M, Plans D, et al. Population risk factors for severe disease and mortality in COVID-19: A global systematic review and meta-analysis. PLOS ONE. 2021;16(3):e0247461.

6. Swift B, Jain L, White C, Chandrasekaran V, Bhandari A, Hughes DA, et al. Innovation at the Intersection of Clinical Trials and Real-World Data Science to Advance Patient Care. Clinical and Translational Science. 2018;11(5):450-60.

7. Henry D, Stehlik P, Camacho X, Pearson SA. Access to routinely collected data for population health research: experiences in Canada and Australia. Australian and New Zealand Journal of Public Health. 2018;42(5):430-3.

8. Henry DA, Jones MA, Stehlik P, Glasziou PP. Effectiveness of COVID-19 vaccines: findings from real world studies. Medical Journal of Australia. 2021;215(4):149-51.e1.

9. Huang Z, Xu S, Liu J, Wu L, Qiu J, Wang N, et al. Effectiveness of inactivated and Ad5-nCoV COVID-19 vaccines against SARS-CoV-2 Omicron BA. 2 variant infection, severe illness, and death. BMC Medicine. 2022;20(1):400.

10. Prasad S, Kalafat E, Blakeway H, Townsend R, O'Brien P, Morris E, et al. Systematic review and meta-analysis of the effectiveness and perinatal outcomes of COVID-19 vaccination in pregnancy. Nature Communications. 2022;13(1):2414.

11. Rahmani K, Shavaleh R, Forouhi M, Disfani HF, Kamandi M, Oskooi RK, et al. The effectiveness of COVID-19 vaccines in reducing the incidence, hospitalization, and mortality from COVID-19: A systematic review and meta-analysis. Frontiers in Public Health. 2022;10.

12. Kow CS, Hasan SS. Real-world effectiveness of BNT162b2 mRNA vaccine: a meta-analysis of large observational studies. Inflammopharmacology. 2021;29(4):1075-90.

13. Tan ST, Kwan AT, Rodríguez-Barraquer I, Singer BJ, Park HJ, Lewnard JA, et al. Infectiousness of SARS-CoV-2 breakthrough infections and reinfections during the Omicron wave. Nature Medicine. 2023;29(2):358-65.

14. Shi X, Li KQ, Mukherjee B. Current Challenges With the Use of Test-Negative Designs for Modeling COVID-19 Vaccination and Outcomes. American Journal of Epidemiology. 2023;192(3):328-33.

15. UK Health Security Agency working with the Medicines & Healthcare products Regulatory Agency. COVID-19 vaccine surveillance report (2 March 2023). In: Care DoHaS, editor. gov.uk2023.

16. Mukherjee B. Being a Public Health Statistician During a Global Pandemic. Statistical Science. 2022;37(2):270-7, 8.

17. The true death toll of COVID-19: estimating global excess mortality: World Health Organization; [cited 2023 March 7]. Available from: https://www.who.int/data/stories/the-true-death-toll-of-covid-19-estimating-global-excess-mortality.

18. DJ Peters M, Godfrey C, McInerney P, Munn Z, Tricco AC, Khalil H. Chapter 11: Scoping Reviews. 2020. In: JBI Manual for Evidence Synthesis [Internet]. https://synthesismanual.jbi.global: JBI.

19. Tricco AC, Lillie E, Zarin W, O'Brien KK, Colquhoun H, Levac D, et al. PRISMA Extension for Scoping Reviews (PRISMA-ScR): Checklist and Explanation. Annals of Internal Medicine. 2018;169(7):467-73.

20. International Vaccine Access Center (IVAC), Johns Hopkins Bloomberg School of Public Health. VIEW-hub. 2023.

21. International Vaccine Access Center, Johns Hopkins Bloomberg School of Public Health, World Health Organization, Coalition for Epidemic Preparedness Innovation. Results of COVID-19 Vaccine Effectiveness & Impact Studies: An Ongoing Systematic Review. Methods.: International Vaccine Access Center (IVAC), Johns Hopkins Bloomberg School of Public Health. VIEW-hub., ; 2022 [updated 10 September 2022. Available from: https://view-hub.org/sites/default/files/2022-09/COVID19_VE_Lit_Review_Methods.pdf.

22. Pratt N, Camacho X, Vajdic C, Degenhardt L, Laba T-L, Hillen J, et al. The Medicines Intelligence Centre of Research Excellence: Co-creating real-world evidence to support the evidentiary needs of Australian medicines regulators and payers. International Journal of Population Data Science. 2021;6(3).

For peer review only

Results of COVID-19 Vaccine Effectiveness & Impact Studies: An Ongoing Systematic Review

Methods

Updated September 10, 2022

For peer review only

Prepared by:

International Vaccine Access Center,
Johns Hopkins Bloomberg School of Public Health

and

World Health Organization

and

Coalition for Epidemic Preparedness Innovations

For comments or questions, please contact: Melissa Higdon at mhigdon@jhu.edu.

Methods for Vaccine Effectiveness (VE) Literature Presented on VIEW-hub and in the Weekly Summary Tables, Visualizations, and Summaries of Policy Gaps

Literature Search

A search of the preprint, and published literature for COVID-19 Vaccine Effectiveness studies is conducted weekly. See Appendix for literature search criteria.

Inclusion Criteria for data abstraction

Title and abstract review are conducted to identify relevant studies for full-text review. During full-text review, a study must contain at least one vaccine effectiveness estimate that meets all the following criteria to be included. This is done to ensure a baseline level of quality and/or comparability of VE estimates, though this does not imply that all studies are Grade A/have minimal risk of bias nor that all excluded studies are of poor quality.

- Published or preprint studies or reports with adequate scientific details. The information cannot come just from a press release, presentations, nor media.
- VE estimates must have confidence intervals around the estimate, except in those cases where it is unable to be calculated.
- All studies must include persons with and without the clinical outcome under investigation and with and without vaccination. Thus, this excludes case only studies, such as impact studies, or those evaluating risk of progression are excluded. This criterion does not apply to transmission studies which evaluate vaccine effectiveness against secondary infection from vaccinated and unvaccinated SARS-CoV-2 cases only, or to booster dose VE studies in which the reference group is persons having completed primary series vaccination.
- The study cannot have a modeled comparison group nor compare to a historical cohort.
- Due to the effect of confounders, the study design should account for confounding and/or the VE estimate should be adjusted or state adjustment made no difference.
- All outcomes must be lab confirmed. As COVID-19 does not have a specific syndrome, studies with syndromic outcomes are excluded.
- At least 90% of participants must have a confirmed vaccination status, rather than relying on recall.
- The study must provide a VE estimate for one vaccine, not for multiple vaccines combined. The exceptions are for 1) studies assessing the combined VE of BNT162b2 (Pfizer) and mRNA-1273 (Moderna) vaccines, 2) studies of heterologous schedules but all participants included in a VE estimate should receive the same brands of vaccines in the same order, and 3) studies of vaccine effectiveness against transmission (due to the scarcity of transmission studies).
- No significant bias that likely affects results
- Cannot include day 0-12 in unvaccinated definition
- Cannot compare to early post vaccination to calculate VE (e.g. day 0-12 vs day 12-21)

A summary table of the main results of studies meeting inclusion criteria can be found on the VIEW-hub Resources page (https://view-hub.org/resources).

For peer review only

Inclusion Criteria for Forest Plots Posed on VIEW-Hub

The VE estimates from eligible studies are plotted in figures. The estimates plotted are a subset of the estimates abstracted from the systematic literature review of those studies meeting additional eligibility criteria. Because a single study can include many VE estimates where the same data appear in more than one VE estimate (e.g., all ages and also separately by age group), criteria are applied to prioritize which to plot in an effort to not overrepresent the amount of evidence that exists for each vaccine. The following criteria are used to determine which VE estimates are displayed in the summary forest plots located on the VIEW-hub resources page (https://view-hub.org/resources):

- Complete vaccination is defined as ≥7 days post final dose; partial vaccination is defined as ≥14 days post first dose of a 2-dose vaccine (current forest plots display VE estimates for complete primary series, first booster dose, and second booster dose; partial vaccination is no longer shown).
- If a study reports results for the same outcome for both combined and individual vaccines, only individual vaccine VE estimates are displayed. This criterion only apples to studies evaluating VE of BNT162b2 (Pfizer) mRNA-1273 (Moderna) vaccines.
- If a study reports results from 2 different evaluation designs (e.g. test-negative design and cohort design) on the same population, VE estimates from the primary analysis only are displayed.
- If a study reports VE estimates for the same disease outcome for different populations, the general population VE estimate is displayed when available. If a general population estimate is not available, the VE from each population is displayed (exception is if there are estimates for similar age groups in which case the more stable VE estimate will be displayed).
- If a study reports VE estimates on more than one 'severe' disease outcome (e.g. 'severe disease', 'hospitalization', and 'ICU admission'), the more inclusive disease outcome including a larger population is displayed. These different types of severe outcomes are labeled as 'severe disease' in the plots, however it is important to keep in mind that the definition of severe disease varies and may explain some differences in VE estimates for severe disease outcomes.
- If a study reports VE estimates for a specific regimen and population at different time intervals since vaccination, the earliest interval of peak VE is selected for the vaccine-specific forest plots (with an exception for the plots on duration of vaccine effectiveness in which multiple time points are plotted). Studies that report only one VE estimate for a specific regimen and population (i.e. at ≥ 14 days post final dose) are included in the vaccine-specific plots and denoted with a '+' after the reference id if the time interval post-vaccination over which VE is measured extends beyond 4 months.

Additional notes

Estimates from mutually exclusive populations in a study can be displayed in the same plot resulting in instances when more than one estimate from a study is plotted (e.g., a study includes VE estimates from two distinct age groups or estimates for different variants).

For studies that report adjusted odds ratios, risk ratios, or rate ratios instead of vaccine effectiveness estimates, VE is calculated as 1 minus the reported effect estimate and multiplying by 100.

Reference numbers are included for each VE estimate displayed so users can identify when a study is represented more than once within a plot. More information on each reference can be found in the weekly literature review summary table located on VIEW-HUB (https://view-hub.org/resources).

Vaccine Effectiveness Studies Database

See accompanying PDF ('CEPI_COVID19VaccineEffectiveness.pdf') of detailed data collection forms for COVID-19 vaccine effectiveness studies. The complete vaccine effectiveness studies database will be made available to CEPI at anytime upon request. In addition, a summary PDF file of all abstracted VE estimates is available on the VIEW-hub Resources page. The same information is also available in a downloadable filterable Excel file ('COVID-19 Vaccine Effectiveness Results Dataset'). These materials are available to the public and updated weekly.

Planned and Ongoing Studies presented on VIEW-hub and summaries of policy gaps

In order to gather information on planned and ongoing studies, a survey was shared with persons conducting and/or funding studies. Data was requested specifically on studies that have completed protocol development to help obtain higher quality data as studies that are still in protocol development are subject to more changes. This data has been compiled by WHO and some key information and summaries of what is planned/ongoing are provided on View Hub and WHO's website.

For peer review only

Appendix: Literature Search Terms

PubMed:

("COVID-19"[tw] OR "COVID 19"[tw] OR "COVID19"[tw] OR "COVID2019"[tw] OR "COVID 2019"[tw] OR "COVID-2019"[tw] OR "novel coronavirus"[tw] OR "new coronavirus"[tw] OR "novel corona virus"[tw] OR "new corona virus"[tw] OR "SARS-CoV-2"[tw] OR "SARSCoV2"[tw] OR "SARS-CoV2"[tw] OR "2019nCoV"[tw] OR "2019-nCoV"[tw] OR "2019 coronavirus"[tw] OR "2019 corona virus"[tw] OR "coronavirus disease 2019"[tw] OR "severe acute respiratory syndrome coronavirus 2"[nm] OR "severe acute respiratory syndrome coronavirus 2"[tw] OR "sars-coronavirus-2"[tw] OR "coronavirus disease 2019"[tw] OR "corona virus disease 2019"[tw])

AND

("COVID-19 Vaccines"[Mesh] OR “COVID-19 vaccine”[tiab] OR "mRNA-1273 vaccine" [Supplementary Concept] OR “mRNA-1273 vaccine”[tiab] OR “mRNA vaccine”[tiab] OR “mRNA COVID-19 vaccines”[tiab] OR "ChAdOx1 COVID-19 vaccine" [Supplementary Concept] OR "Ad5-nCoV vaccine" [Supplementary Concept] OR “Ad5-nCoV”[tiab] OR "Covid-19 aAPC vaccine" [Supplementary Concept] OR "Ad26.COV2.S vaccine" [Supplementary Concept] OR "Ad26.COV2.S vaccine"[tiab] OR “adenoviral vector vaccine”[tiab] OR "BNT162 vaccine" [Supplementary Concept] OR “BNT162b2”[tiab] OR “BNT162”[tiab] OR “CoronaVac” [tiab] OR “vaccin*”[tiab])

AND

("Clinical Trial, Phase IV" [Publication Type] OR "Controlled Clinical Trial" [Publication Type] OR "Randomized Controlled Trial" [Publication Type] OR "Case-Control Studies"[Mesh] OR "Retrospective Studies"[Mesh] OR “Retrospective”[tiab] OR "Cohort Studies"[Mesh] OR "Prospective Studies"[Mesh] OR “Prospective”[tiab] OR "Longitudinal Studies"[Mesh] OR "Follow-Up Studies"[Mesh] OR “Follow-up studies”[tiab] OR “cohort”[tiab] OR "test negative"[tiab] OR “Observational cohort”[tiab] OR “Test-negative design”[tiab] OR"RCT"[tiab] OR "randomized"[tiab] OR "randomised"[tiab] OR "randomly allocated"[tiab] OR “case-control”[tiab] OR “real-world effectiveness”[tiab] OR “effectiveness”[tiab] OR “association”[tiab] OR “impact”[tiab] OR “vaccine impact”[tiab]) NOT ("Clinical Trial, Phase I" [Publication Type] OR "Clinical Trial, Phase II" [Publication Type])
NOT (“animals”[mesh] NOT (“animals”[mesh] AND “humans”[mesh]))

Embase:

('COVID-19' OR 'COVID 19' OR 'COVID19' OR 'COVID2019' OR 'COVID 2019' OR 'COVID-2019' OR 'novel coronavirus' OR 'new coronavirus' OR 'novel corona virus' OR 'new corona virus' OR 'SARS-CoV-2' OR 'SARSCoV2' OR 'SARS-CoV2' OR '2019nCoV' OR '2019-nCoV' OR '2019 coronavirus' OR '2019 corona virus' OR 'coronavirus disease 2019' OR 'severe acute respiratory

For peer review only

syndrome coronavirus 2'/exp OR 'severe acute respiratory syndrome coronavirus 2' OR 'sars-coronavirus-2' OR 'coronavirus disease 2019'/exp OR 'coronavirus disease 2019' OR 'corona virus disease 2019')

AND

('SARS-CoV-2 vaccine'/exp OR 'COVID-19 vaccine':ti,ab OR 'mRNA-1273 vaccine'/exp OR 'mRNA-1273 vaccine':ti,ab OR 'mRNA vaccine':ti,ab OR 'mRNA COVID-19 vaccines':ti,ab OR 'ChAdOx1 ncov 19'/exp OR 'Ad5 nCoV vaccine'/exp OR 'Ad5-nCoV':ti,ab OR 'Covid-19 aAPC vaccine':ti,ab OR 'Ad26.COV2.S vaccine'/exp OR 'Ad26.COV2.S vaccine':ti,ab OR 'adenoviral vector vaccine':ti,ab OR 'BNT 162 vaccine'/exp OR 'BNT162b2':ti,ab OR 'BNT162':ti,ab OR 'CoronaVac'/exp OR 'coronavac':ti,ab OR 'vaccin*':ti,ab)

AND

('phase 4 clinical trial'/exp OR 'Controlled Clinical Trial'/exp OR 'Randomized Controlled Trial'/exp OR 'Case Control Study'/exp OR 'Retrospective Study'/exp OR 'Retrospective':ti,ab OR 'Cohort analysis'/exp OR 'Prospective Study'/exp OR 'Prospective':ti,ab OR 'Longitudinal Study'/exp OR 'Follow Up'/exp OR 'Follow-up study':ti,ab OR 'cohort':ti,ab OR 'test negative':ti,ab OR 'Observational cohort':ti,ab OR 'postmarketing surveillance'/exp OR 'postmarketing surveillance':ti,ab OR 'Test-negative design':ti,ab OR 'RCT':ti,ab OR 'randomized':ti,ab OR 'randomised':ti,ab OR 'randomly allocated':ti,ab OR 'case-control':ti,ab OR 'real-world effectiveness':ti,ab OR 'effectiveness':ti,ab OR 'association':ti,ab) NOT ('phase 1 clinical trial'/exp OR 'phase 2 clinical trial'/exp)
NOT ('animal'/exp NOT ('animal'/exp AND 'human'/exp))
NOT 'conference abstract'/it

WHO COVID database:

("COVID-19 Vaccines" OR "COVID-19 vaccine" OR "mRNA-1273 vaccine" OR "mRNA vaccine" OR "mRNA COVID-19 vaccines" OR "ChAdOx1 COVID-19 vaccine" OR "Ad5-nCoV" OR "Covid-19 aAPC vaccine" OR "Ad26.COV2.S vaccine" OR "adenoviral vector vaccine" OR "BNT162b2" OR "BNT162" OR "CoronaVac" OR vaccin*)

AND

("Phase IV" OR "Controlled Clinical Trial" OR "Randomized Controlled Trial" OR "Case-Control Studies" OR "Retrospective" OR "Cohort Studies" OR "Prospective" OR "Longitudinal Studies" OR "Follow-Up Studies" OR "Follow-up study" OR "cohort" OR "test negative" OR "Observational cohort" OR "Test-negative design" OR "RCT" OR "randomized" OR "randomised" OR "randomly allocated" OR "case-control" OR "real-world effectiveness" OR "effectiveness" OR "association") AND NOT ("Phase I" OR "Phase II")

SCOPUS:

For peer review only

TITLE-ABS-KEY(“novel coronavir*” OR “novel corona virus*” OR “2019 coronavirus” OR betacoronavir* OR covid19 OR "covid 19" OR ncov OR "CoV 2" OR cov2 OR sarscov2 OR sars-cov OR sarscov OR 2019ncov OR 2019-nCoV OR "novel CoV" OR “coronavirus infections”) AND TITLE-ABS-KEY(Vaccin* AND (effectiveness OR efficacy OR protection*) AND (postmarketing OR approved OR (post* W/5 approval) OR "real world" OR "phase IV" OR "phase 4" OR observational OR longitudinal OR spread OR transmission OR (rate* W/5 infection*) OR (reduc* W/5 infection*) OR "general population"))

Web of Science:

(TI=(covid-19 vaccine effectiveness)) OR AB=(covid-19 vaccine effectiveness)

medRxiv, bioRxiv, SSRN, Europe PMC, Research Square, Knowledge Hub:

“COVID-19 vaccine effectiveness” OR “COVID-19 vaccine efficacy”

In addition to the above databases, MMWR, and Eurosurveillance are hand-searched weekly for new studies meeting eligibility criteria.

For peer review only

Preferred Reporting Items for Systematic reviews and Meta-Analyses extension for Scoping Reviews (PRISMA-ScR) Checklist

SECTION	ITEM	PRISMA-ScR CHECKLIST ITEM	REPORTED ON PAGE #
**TITLE**			
Title	1	Identify the report as a scoping review.	1
**ABSTRACT**			
Structured summary	2	Provide a structured summary that includes (as applicable): background, objectives, eligibility criteria, sources of evidence, charting methods, results, and conclusions that relate to the review questions and objectives.	2
**INTRODUCTION**			
Rationale	3	Describe the rationale for the review in the context of what is already known. Explain why the review questions/objectives lend themselves to a scoping review approach.	4-5
Objectives	4	Provide an explicit statement of the questions and objectives being addressed with reference to their key elements (e.g., population or participants, concepts, and context) or other relevant key elements used to conceptualize the review questions and/or objectives.	5
**METHODS**			
Protocol and registration	5	Indicate whether a review protocol exists; state if and where it can be accessed (e.g., a Web address); and if available, provide registration information, including the registration number.	2
Eligibility criteria	6	Specify characteristics of the sources of evidence used as eligibility criteria (e.g., years considered, language, and publication status), and provide a rationale.	6-7
Information sources*	7	Describe all information sources in the search (e.g., databases with dates of coverage and contact with authors to identify additional sources), as well as the date the most recent search was executed.	6
Search	8	Present the full electronic search strategy for at least 1 database, including any limits used, such that it could be repeated.	Supp 1
Selection of sources of evidence†	9	State the process for selecting sources of evidence (i.e., screening and eligibility) included in the scoping review.	7-8
Data charting process‡	10	Describe the methods of charting data from the included sources of evidence (e.g., calibrated forms or forms that have been tested by the team before their use, and whether data charting was done independently or in duplicate) and any processes for obtaining and confirming data from investigators.	7-8
Data items	11	List and define all variables for which data were sought and any assumptions and simplifications made.	7-8
Critical appraisal of individual sources of evidence§	12	If done, provide a rationale for conducting a critical appraisal of included sources of evidence; describe the methods used and how this information was used in any data synthesis (if appropriate).	8

St. Michael's
Inspired Care.
Inspiring Science.

For peer review only - http://bmjopen.bmj.com/site/about/guidelines.xhtml

SECTION	ITEM	PRISMA-ScR CHECKLIST ITEM	REPORTED ON PAGE #
Synthesis of results	13	Describe the methods of handling and summarizing the data that were charted.	8
**RESULTS**			
Selection of sources of evidence	14	Give numbers of sources of evidence screened, assessed for eligibility, and included in the review, with reasons for exclusions at each stage, ideally using a flow diagram.	TBC
Characteristics of sources of evidence	15	For each source of evidence, present characteristics for which data were charted and provide the citations.	TBC
Critical appraisal within sources of evidence	16	If done, present data on critical appraisal of included sources of evidence (see item 12).	TBC
Results of individual sources of evidence	17	For each included source of evidence, present the relevant data that were charted that relate to the review questions and objectives.	TBC
Synthesis of results	18	Summarize and/or present the charting results as they relate to the review questions and objectives.	TBC
**DISCUSSION**			
Summary of evidence	19	Summarize the main results (including an overview of concepts, themes, and types of evidence available), link to the review questions and objectives, and consider the relevance to key groups.	TBC
Limitations	20	Discuss the limitations of the scoping review process.	TBC
Conclusions	21	Provide a general interpretation of the results with respect to the review questions and objectives, as well as potential implications and/or next steps.	TBC
**FUNDING**			
Funding	22	Describe sources of funding for the included sources of evidence, as well as sources of funding for the scoping review. Describe the role of the funders of the scoping review.	10

JBI = Joanna Briggs Institute; PRISMA-ScR = Preferred Reporting Items for Systematic reviews and Meta-Analyses extension for Scoping Reviews.
* Where *sources of evidence* (see second footnote) are compiled from, such as bibliographic databases, social media platforms, and Web sites.
† A more inclusive/heterogeneous term used to account for the different types of evidence or data sources (e.g., quantitative and/or qualitative research, expert opinion, and policy documents) that may be eligible in a scoping review as opposed to only studies. This is not to be confused with *information sources* (see first footnote).
‡ The frameworks by Arksey and O'Malley (6) and Levac and colleagues (7) and the JBI guidance (4, 5) refer to the process of data extraction in a scoping review as data charting.
§ The process of systematically examining research evidence to assess its validity, results, and relevance before using it to inform a decision. This term is used for items 12 and 19 instead of "risk of bias" (which is more applicable to systematic reviews of interventions) to include and acknowledge the various sources of evidence that may be used in a scoping review (e.g., quantitative and/or qualitative research, expert opinion, and policy document).

From: Tricco AC, Lillie E, Zarin W, O'Brien KK, Colquhoun H, Levac D, et al. PRISMA Extension for Scoping Reviews (PRISMAScR): Checklist and Explanation. Ann Intern Med. 2018;169:467–473. doi: 10.7326/M18-0850.

For peer review only - http://bmjopen.bmj.com/site/about/guidelines.xhtml
